# Stereoselective Synthesis, Configurational Assignment and Biological Evaluations of the Lipid Mediator RvD2_n‐3 DPA_

**DOI:** 10.1002/chem.202103857

**Published:** 2021-12-28

**Authors:** Amalie F. Reinertsen, Karoline G. Primdahl, Roberta De Matteis, Jesmond Dalli, Trond V. Hansen

**Affiliations:** ^1^ Department of Pharmacy Section for Pharmaceutical Chemistry University of Oslo P.O. Box 1068 0316 Oslo Norway; ^2^ Lipid Mediator Unit Center for Biochemical Pharmacology William Harvey Research Institute Barts and The London School of Medicine Queen Mary University of London Charterhouse Square London EC1M 6BQ United Kingdom

**Keywords:** n-3 docosapentaenoic acid, resolution of bacterial infection and inflammation, resolvins, RvD2_n-3 DPA_, specialized pro-resolving mediators

## Abstract

Herein we report the first total synthesis of RvD2_n‐3 DPA_, an endogenously formed mediator biosynthesized from the omega‐3 fatty acid n‐3 docosapentaenoic acid. The key steps are the Midland Alpine borane reduction, Sonogashira cross‐coupling reactions, and a *Z*‐selective alkyne reduction protocol, yielding RvD2_n‐3 DPA_ methyl ester in 13 % yield over 12 steps (longest linear sequence). The physical property data (UV chromophore, chromatography and MS/MS fragmentation) of the synthetic lipid mediator matched those obtained from biologically produced material. Moreover, synthetic RvD2_n‐3 DPA_ also carried the potent biological activities of enhancing macrophage uptake of *Staphylococcus aureus* and zymosan A bioparticles.

## Graphical abstract

**RvD2_n‐3 DPA_, a specialized pro‐resolving mediator**, is synthesized for the first time based on the retrosynthetic outline depicted, enabling its structural elucidation and biological evaluations against *S. aureus* and zymosan A induced inflammation.

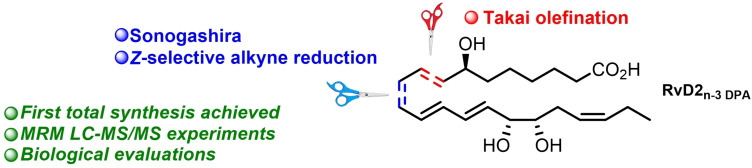

## Introduction

The inflammatory process is an essential part of the normal protective response to tissue injury and infection by invading pathogens and is divided into acute and chronic inflammation.^[1]^ Although the primary goal of the inflammation phase is to regain homeostasis,^[2]^ if kept uncontrolled, it may result in the development of a highly unappreciated outcome, namely a chronic state of inflammation. Chronic inflammation is associated with diseases such as rheumatoid arthritis, cardiovascular disorders, Parkinson and Alzheimer's diseases.^[3]^ However, the acute inflammatory response is normally self‐limited and resolves smoothly. This self‐contained process is divided into two distinct phases: the initiation and the resolution phase. The resolution of the inflammatory process is now considered to be a dynamic, programmed response, and not just a means of passive dilution of chemoattractants, as previously thought.^[4]^ Further, evidence for this emerged with the discovery of oxygenated lipid mediators possessing pro‐resolving abilities biosynthesized from the ω‐3 polyunsaturated fatty acids (PUFAs) docosahexaenoic acid (DHA), eicosapentaenoic acid (EPA), and n‐3 docosapentaenoic acid (n‐3 DPA).[^5^, ^6^, ^7^]

**Figure 1:**
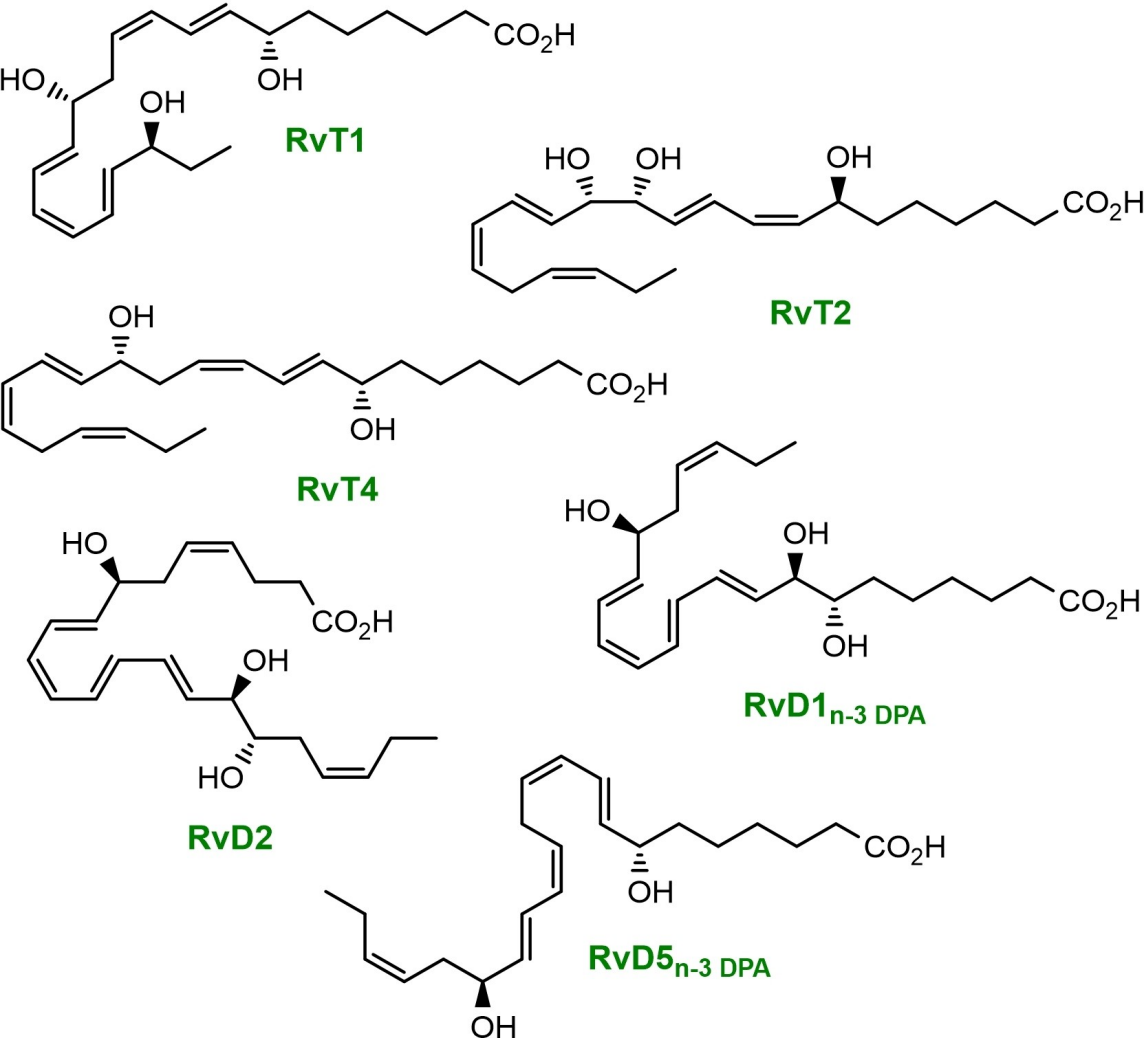
Chemical structures of some resolvins derived from n‐3 DPA and the DHA‐derived RvD2.

New drugs that can resolve inflammation without undesirable side effects are of great interest in drug discovery programs,^[8]^ and are therefore attractive targets for total synthesis.^[9]^ The pro‐resolving endogenously formed resolvins are excellent candidates displaying these actions in vivo^[10]^ and belong to a superfamily called specialized pro‐resolving mediators (SPMs). SPMs are derived from ω‐3 PUFAs and interact with G‐protein coupled receptors (GPCRs) on the cell surface, hence limiting the infiltration of polymorph nuclear neutrophils (PMNs) and enhance the clearance of apoptotic cells by phagocytosis^[11]^ – two important features in controlling inflammation. More in‐depth insight into the importance of SPMs in the resolution phase of an inflammatory process may lead to the development of new treatments of inflammatory driven diseases.^[4]^ Thus, the resolution of inflammation controlled by SPMs is considered a biomedical paradigm shift.^[12]^ The chemical structures of some resolvins are depicted in Figure 1.

## Results and Discussion

**Scheme 1:**
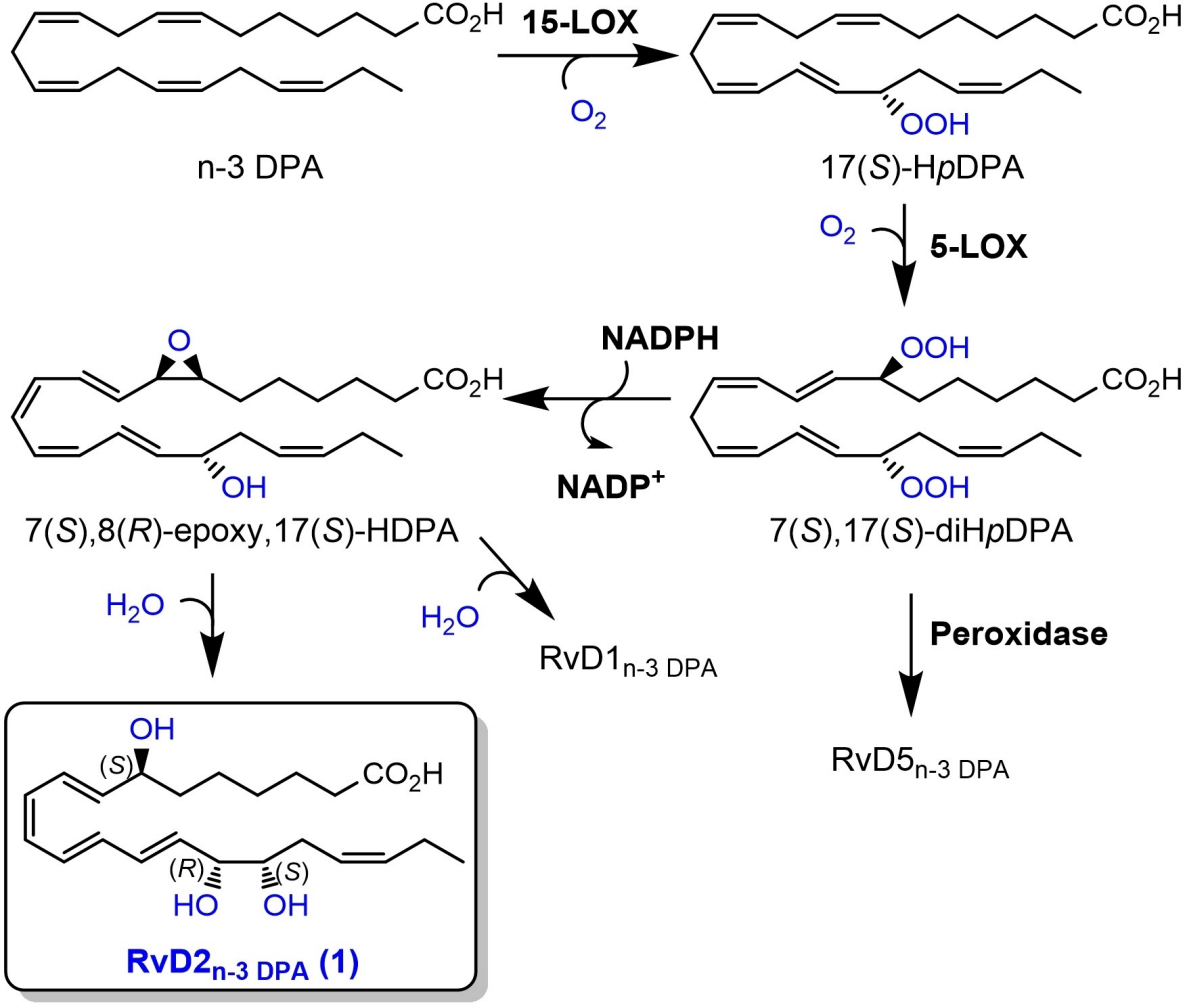
Proposed biosynthesis of RvD2_n‐3 DPA_ (**1**), RvD1_n‐3 DPA_, and RvD5_n‐3 DPA_ from n‐3 DPA.

The DHA‐derived SPM resolvin D2 (RvD2, see Figure 1) was isolated from inflammatory exudates and its structure was later established by LC‐MS/MS experiments^[13]^ and total synthesis.[^14^, ^15^] RvD2 reduces excessive neutrophilic trafficking to inflammatory loci and has been reported to decrease leukocyte‐endothelial interactions in vivo by endothelial‐dependent nitric oxide production and by direct modulations of leukocyte adhesion receptor expression[^10^, ^11^] Additionally, RvD2 decrease both local and systemic bacterial burden, excessive cytokine production and neutrophil recruitment,^[16a]^ while increasing peritoneal mononuclear cells and macrophage phagocytosis.^[16b]^ These initial reports spurred an interest in evaluating other biological properties of this SPM.[^16b^, ^16c^, ^16d^] The congener of RvD2, RvD2_n‐3 DPA_ (**1**), was reported in 2013 and its structure elucidated based on UV and LC‐MS/MS data.^[5]^ This SPM is derived from n‐3 DPA and formed after two consecutive lipoxygenation reactions and epoxide formation, followed by ring opening of the epoxide by a hydrolytic enzyme (Scheme 1). In the anticipated biogenetic formation of **1**, the first biosynthetic step in the presence of 15‐LOX forms 17(*S*)‐H*p*DPA, while the second lipoxygenation step is catalyzed by 5‐LOX. The product herein, 7(*S*),17(*S*)‐diH*p*DPA, is then subjected to enzymatic conversion to an epoxide intermediate that is next enzymatically hydrolyzed to either RvD1_n‐3 DPA_ (Figure 1) or RvD2_n‐3 DPA_ (**1**), see Scheme 1. Alternatively, direct reduction of the hydroperoxide intermediate 7(*S*),17(*S*)‐diH*p*DPA, catalyzed by peroxidase enzymes, produces RvD5_n‐3 DPA_ (see Figure 1 and Scheme 1).

**Figure 2:**
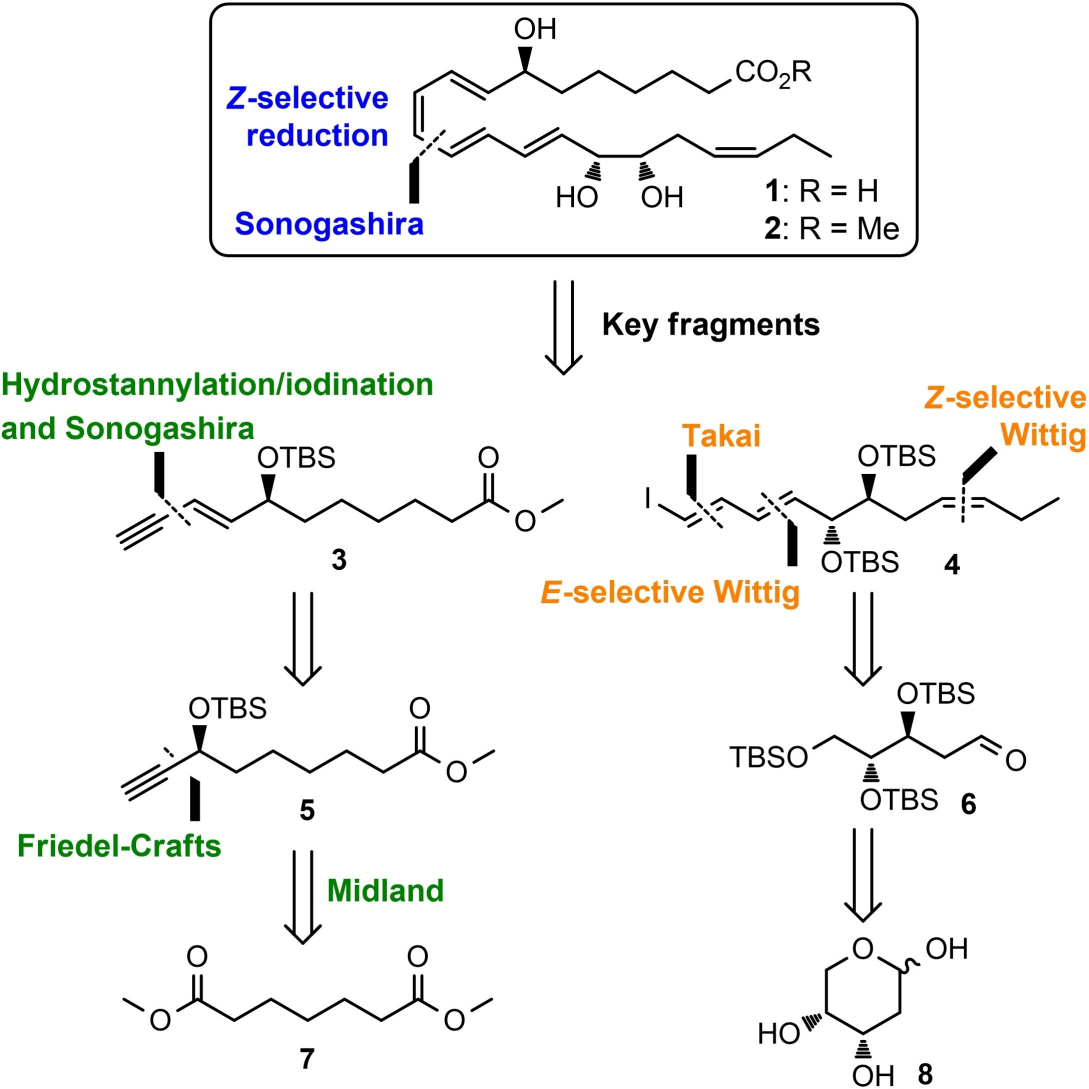
Overview of the retrosynthetic analysis of RvD2_n‐3 DPA_ (**1**).

Biosynthetic considerations, LC–MS/MS data, and physical properties (MS‐ and UV‐Vis data) of the isolated endogenously produced material gave evidence for the proposed structure of **1** (Figure 2) with a highly sensitive *E*,*Z*,*E*,*E*‐tetraene embedded by two chiral allylic alcohols. An overview of the retrosynthetic proposal applied to the tentatively assigned structure of RvD2_n‐3 DPA_ (**1**) suggest a disconnection based on the Sonogashira cross‐coupling reaction^[17]^ followed by *Z*‐selective reduction. The analysis identified two key fragments, **3** and **4**, to be convergently assembled in the synthesis.

Fragment **3** was disconnected back to alkyne **5**, to be prepared from commercially available dimethyl pimelate (**7**) with an aliphatic Friedel‐Crafts acylation,^[18]^ Midland Alpine borane reduction,^[19]^ and Sonogashira cross‐coupling. Two subsequent Wittig reactions and a Takai olefination^[20]^ were chosen as the key transformations for finishing fragment **4** from 2‐deoxy‐β‐d‐ribopyranose (**8**).

**Scheme 2:**
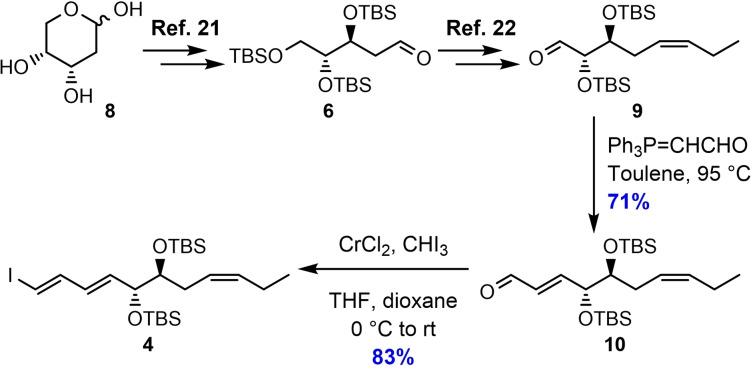
Synthesis of vinylic iodide **4**.

The project commenced with the construction of compound **4**, starting from commercially available and affordable **8**, as shown in Scheme 2. Aldehyde **9** was prepared according to the literature,[^21^, ^22^] and was further reacted in an *E*‐selective Wittig reaction with the stabilized ylide (triphenylphosphoranylidene)‐acetaldehyde at elevated temperature, to give the α,β‐unsaturated aldehyde **10**. A Takai olefination protocol was then performed to complete the synthesis of vinyl iodide **4** in 59 % yield over two steps as one geometrical isomer after purification using column flash chromatography (Supporting Information).

**Scheme 3:**
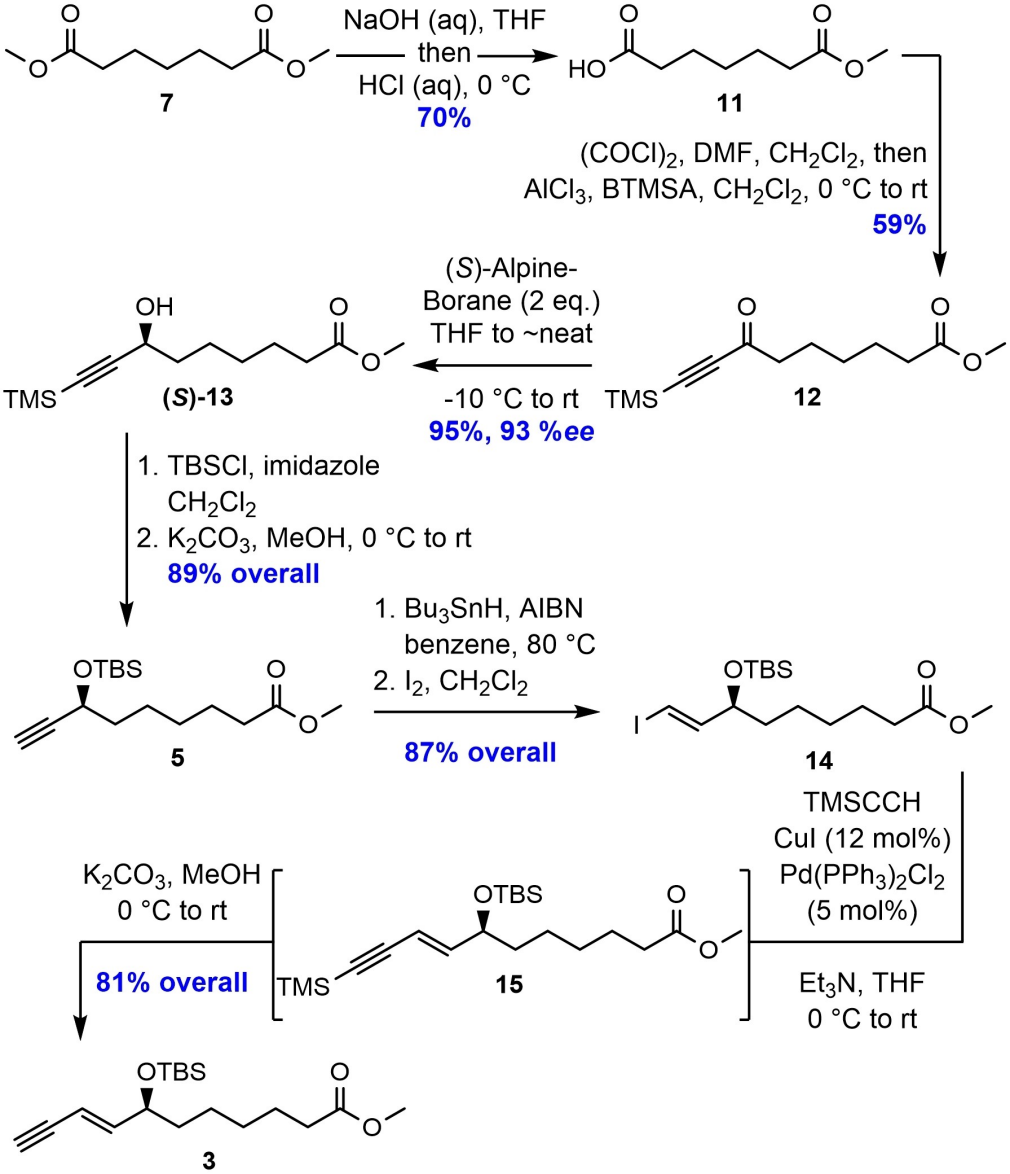
Preparation of the alkyne ester **3**.

For the synthesis of alkyne ester **3**, a selective monohydrolysis of dimethyl pimelate (**7**) using aqueous NaOH in THF, followed by acidic work‐up, was performed to give carboxylic acid **11** in 70 % yield (see Scheme 3).^[23]^ Next, the corresponding acid chloride of **11** was prepared in situ and submitted to an aliphatic Friedel‐Crafts acylation, giving ketone **12** in 59 % yield. The asymmetric reduction of the alkynyl ketone was achieved by the addition of the Midland (*S*)‐Alpine borane reagent in THF at −10 °C, followed by swift removal of the solvent to essentially neat conditions, yielding the desired propargylic alcohol (*S*)‐**13** in 93 % enantiomeric excess (*ee*) and 95 % yield after workup and purification (Supporting Information). The *ee*‐value was determined by HPLC‐analyses of the 2‐naphthoate derivative of **13** (Supporting Information).

The secondary alcohol (*S*)‐**13** was then treated with TBS chloride and imidazole in dichloromethane, followed by TMS‐deprotection using potassium carbonate in methanol to yield **5** in 89 % over the two steps. At this point, it was necessary to convert the terminal acetylene **5** into the corresponding *E*‐vinyl iodide **14**. This was achieved by first treating the compound with a catalytic amount of azobisisobutyronitrile (AIBN) and excess tributyltin hydride at elevated temperature, followed by iododestannylation, to give **14** in 87 % yield over the two steps. The first of the two planned palladium catalyzed reactions was accomplished using catalytic amounts of Pd(PPh_3_)_2_Cl_2_/CuI in THF under basic conditions, which united vinyl iodide **14** with commercially available trimethylsilylacetylene. Direct protodesilylation of the crude compound **15** was achieved after treatment with potassium carbonate in methanol, affording the terminal alkyne **3** in 81 % yield.

**Scheme 4:**
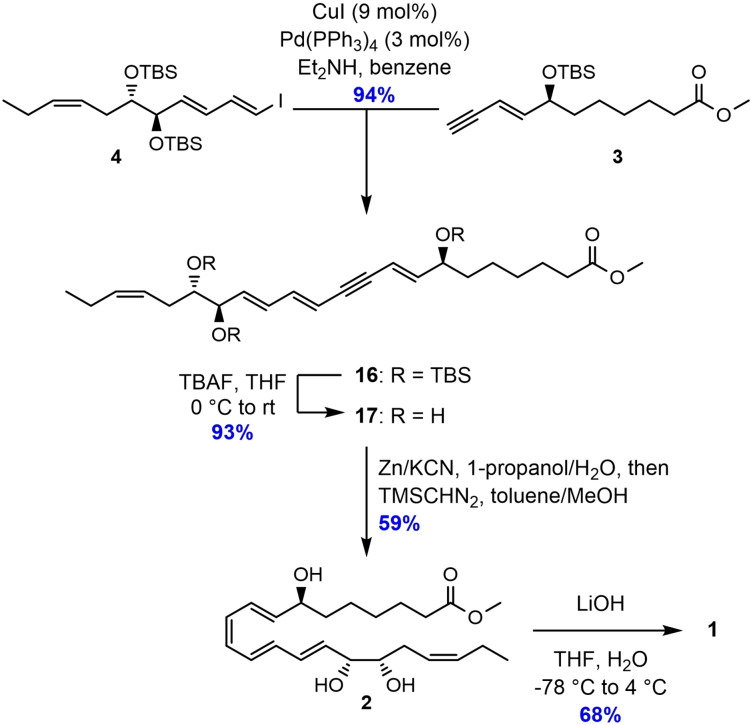
Sonogashira cross‐coupling reaction and *Z*‐selective hydrogenation to complete the synthesis of RvD2_n‐3 DPA_ methyl ester (**2**).

For the assembly of the two key fragments, **3** and **4**, the Sonogashira cross‐coupling reaction using Pd(PPh_3_)_4_/CuI and diethylamine in benzene produced **16** in excellent yields (Scheme 4). Removal of the three TBS‐groups in **16** was performed by adding tetra‐*n*‐butylammonium fluoride (TBAF) in THF to give the triol **17** in 93 % yield. However, for the *Z*‐selective reduction of the internal alkyne in **17**, several different protocols were attempted. Firstly, a Zn(Cu/Ag) mediated reduction protocol,^[24]^ reported to be highly *Z*‐selective for conjugated systems, was tested, but the conversion was rather poor. Additionally, elimination of the (7*S*)‐alcohol moiety was observed. Next, a Lindlar hydrogenation protocol^[25]^ using a mixture of EtOAc/pyridine/1‐octene as solvent system was attempted, but no product formation was observed. Then, a hydrosilylation protocol^[26]^ using the Karstedt catalyst was tried, which was successfully employed in the preparation of RvD1_n‐3 DPA_.^[21]^ Unfortunately, major byproduct formation and difficulties in the purification step was observed. Finally, a reaction using zinc powder and potassium cyanide in a mixture of 1‐propanol/H_2_O,^[27]^ followed by a solvent switch to toluene/methanol and addition of TMS‐diazomethane, gave RvD2_n‐3 DPA_ methyl ester (**2**) in 59 % yield and with chemical purity >96 % after purification by column chromatography (Supporting Information). The NMR‐ (^1^H, ^13^C, and COSY), MS‐, and UV‐data were all in accordance with the structure of **2** (Supporting Information).

MRM LC‐MS/MS matching experiments were performed to assure that our synthetic material was identical to that of authentic RvD2_n‐3 DPA_ (**1**). For SPMs, direct NMR analyses for structural elucidation and configurational assignments are not possible, since their biosynthetic formation yields nano‐ to picogram amounts.^[9a]^ As previously reported, SPMs are chemically labile compounds.^[28]^ Hence, hydrolysis of the methyl ester **2** to the free acid **1** was performed just prior to the LC‐MS/MS experiments,^[5]^ since the *E*,*Z*,*E*,*E*‐tetraene in SPMs easily undergo conversion to other geometrical isomers.^[1]^

**Figure 3:**
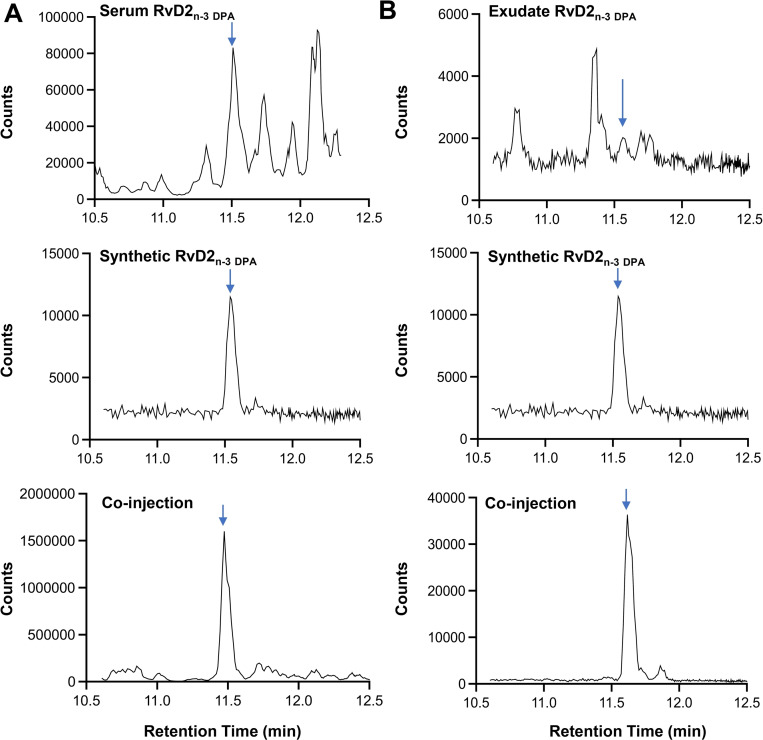
Synthetic RvD2_n‐3 DPA_ (**1**) elutes at the same retention time as endogenous RvD2_n‐3 DPA_ (**1**) in human serum and mouse inflammatory exudates. (A) Human serum was obtained from commercial sources and (B) inflammatory exudates were collected from mice 2 h after inoculation of *E. coli* (10^5^ CFU) via peritoneal lavage. Results are from n=3 determinations for A and n=4 mice for B. See Supporting Information for details.

Injection of biological material obtained from human peripheral blood and mouse infectious exudate with synthetic materials gave a single sharp peak (R_T_=11.5 min). Co‐elution of the synthetic material with biological material was further corroborated by co‐injecting the synthetic material with biological material (see Figure 3). In addition, the UV‐spectrum of **1** (λ_max_ (EtOH)=301 nm, shoulders at 288 and 315 nm, Figure S‐1, Supporting Information), is in agreement with earlier reported data of RvD2_n‐3 DPA_ (**1**)^[5]^ and RvD2.[^7^, ^14^]

Additionally, the MS/MS spectra of biologically and synthetically produced material of **1** (Figure S‐2, Supporting Information), confirmed that the synthetically produced compound matched the data of the biogenic material. Key diagnostic fragments were identified in MS/MS spectra from biological and synthetic material, including *m/z* 143, 197, 233 and 249 (Figure S‐2, Supporting Information).

**Figure 4:**
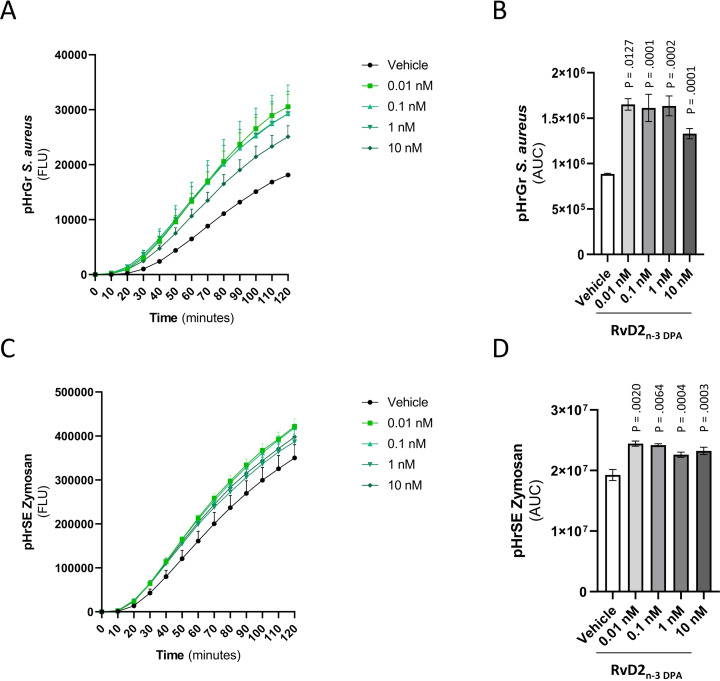
RvD2_n‐3 DPA_ (**1**) increases bone marrow‐derived macrophages uptake of *S. aureus* and zymosan A bioparticles. Results in A and C are expressed as change in signal intensity recorded at baseline (0 min), (mean ± s.e.m., N=3). Results in B and D are expressed as AUC values (mean ± s.e.m., N=3). See Supporting Information for details.

In order to investigate the biological properties of the synthetic material, murine macrophages were differentiated from bone marrows in vitro. Macrophages were incubated with either vehicle or RvD2_n‐3 DPA_ (**1**) for 15 minutes before adding pHrodo‐labelled *Staphylococcus aureus* or zymosan A bioparticles, as previously described.^[29]^ Here we observed that synthetic RvD2_n‐3 DPA_ (**1**) potently increased the uptake of both *S. aureus* (Figure 4 A–B) and zymosan A bioparticles (Figure 4 C–D) in a dose dependent matter. *S. aureus* is a bacterium commonly located on the skin and mucosa of healthy individuals that can cause serious infections if in contact with internal tissues or by dissemination through the bloodstream.^[29]^ Zymosan A is a macromolecule derived from the yeast wall of *Saccharomyces cerevisiae*, commonly used to induce sterile inflammation. Our findings demonstrate that RvD2_n‐3 DPA_ (**1**) not only promotes the uptake of bacterial and fungal particles, but also their degradation since the dye employed is a pH‐sensitive dye and thus indicates that this mediator also promotes the phagolysosome acidification, a key step in microbial killing. Clearing of such pathogens and inflammatory molecules by tissue‐infiltrating macrophages is a fundamental step in the resolution of inflammation that, when impaired, can lead to increased tissue damage and systemic inflammation.^[29]^ Notably, SPMs have been demonstrated to effectively increase the clearance of pathogens in several inflammatory settings.[^30^, ^31^]

## Conclusion

In summary, the first total synthesis of the methyl ester **2** of the specialized pro‐resolving mediator RvD2_n‐3 DPA_ (**1**) has been stereoselectively obtained in 13 % overall yield over 12 steps (longest linear sequence). The synthesis featured the rarely used synthetic method of *Z*‐selective alkyne hydrogenation using potassium cyanide and zinc. Our synthesis differs significantly to earlier reported synthesis of the congener RvD2,[^14^, ^15^] and produced multi milligrams of **2**. The stereoselective synthesis of RvD2_n‐3 DPA_ (**1**) and data from LC‐MS/MS matching experiments enabled the configurational assignment of this oxygenated natural product as (7*S*,8*E*,10*Z*,12*E*,14*E*,16*R*,17*S*,19*Z*)‐7,16,17‐trihydroxydocosa‐8,10,12,14,19‐pentaenoic acid. In addition, using synthetic material of **1** in the 0.01 to 10 nM range, experiments showed that **1** potently increases bone marrow‐derived macrophage uptake of *S. aureus* and zymosan A bioparticles. Such bioactions are of interest towards developing new immunoresolvents[^8^, ^30^, ^31^] targeting development of new anti‐bacterial drugs.^[32]^

## Conflict of interest

The authors declare no conflict of interest.

1

## Supporting information

As a service to our authors and readers, this journal provides supporting information supplied by the authors. Such materials are peer reviewed and may be re‐organized for online delivery, but are not copy‐edited or typeset. Technical support issues arising from supporting information (other than missing files) should be addressed to the authors.

Supporting Information

## Data Availability

The data that support the findings of this study are available in the supplementary material of this article.
