# Supplementary material for: Stereoselective Synthesis, Configurational Assignment and Biological Evaluations of the Lipid Mediator RvD2n‐3 DPA
Source: Chemistry. 2021 Dec 28;28(7):e202103857. doi: 10.1002/chem.202103857 (PMC9305452; doi:10.1002/chem.202103857)
Supplement: Supplementary file 1 — Supporting Information [file CHEM-28-0-s001.pdf]

Chemistry–A European Journal

Supporting Information

Stereoselective Synthesis, Configurational Assignment and Biological Evaluations of the Lipid Mediator RvD2$_{n\text{-}3\ DPA}$

Amalie F. Reinertsen, Karoline G. Primdahl, Roberta De Matteis, Jesmond Dalli, and Trond V. Hansen*

Author contributions

A. F. R. Data curation: Lead; Formal analysis: Lead; Investigation: Lead; Validation: Lead; Visualization: Lead; Writing – review & editing: Equal

K. G. P. Data curation: Supporting; Formal analysis: Supporting; Writing – review & editing: Supporting

R. De M.: Data curation: Supporting; Formal analysis: Equal; Investigation: Supporting; Writing – review & editing: Supporting

J. D. Funding acquisition: Supporting; Formal analysis: Supporting; Investigation: Supporting; Supervision: Supporting; Writing – review & editing: Supporting

T. V. H. Conceptualization: Lead; Formal analysis: Supporting; Funding acquisition: Lead; Investigation: Supporting; Project administration: Lead; Resources: Lead; Visualization: Supporting; Writing – review & editing: Equal

Supporting information

Contents

General Information.....2

Experimental Details.....3

UV and MS experiments of authentic RvD2 $_{n\text{-}3\ DPA}$ (**1**).....15

Evaluations of bone marrow-derived macrophage differentiation.....17

^{1}H- and ^{13}C{^{1}H} NMR Spectra.....19

HPLC Chromatograms.....41

UV-Vis Chromatograms.....44

HRMS Chromatograms.....45

References.....59

General Information

Unless otherwise stated, all commercially available reagents and solvents were used in the form they were supplied without any further purification. The stated yields are based on the isolated material. All sensitive reactions were performed under an argon atmosphere using Schlenk techniques. Reaction flasks were covered with aluminum foil during sensitive reactions and storage to minimize exposure to light. Thin layer chromatography was performed on silica gel 60 F_{254} aluminum-backed plates fabricated by Merck. Flash column chromatography was performed on silica gel 60 (40 - 63 µm) fabricated by Merck. NMR spectra were recorded on a Bruker AVII 400 spectrometer at 400 MHz for ^{1}H NMR and at 101 MHz for ^{13}C NMR. Coupling constants (*J*) are reported in hertz and chemical shifts are reported in parts per million (δ) relative to the central residual protium solvent resonance in ^{1}H NMR ($CDCl_3$ = δ 7.27, CD_3OD = δ 3.31, and C_6D_6 = δ 7.16) and the central carbon solvent resonance in ^{13}C NMR ($CDCl_3$ = δ 77.0 ppm, CD_3OD = δ 49.0, and C_6D_6 = δ 128.1). High-resolution mass spectra were recorded at 70 eV on a Micromass Prospec Q or Micromass QTOF 2W spectrometer using ESI as the method of ionization. Optical rotations were measured using a 0.7 mL cell with a 1.0 dm path length on a PerkinElmer 341 polarimeter. HPLC-analyses were performed using an AD-H stationary phase (CHIRALPAK, 4.6 x 250 mm, particle size 5 µm, from Daicel Corporation) or a C_{18} stationary phase (Eclipse XDBC_{18}, 4.6 x 250 mm, particle size 5 µm, from Agilent Technologies), applying the conditions stated. The UV-Vis spectrum was recorded using an Agilent Technologies Cary 8485 UV-Vis spectrophotometer using quartz cuvettes.

Experimental Details

(2*R*,3*S*)-5,5-*Bis*(ethylthio)pentane-1,2,3-triol (18)

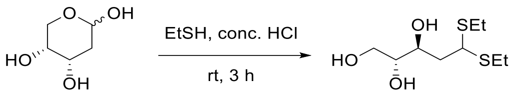

Compound **8** (5.01 g, 37.4 mmol, 1.00 equiv.) was dissolved in concentrated hydrochloric acid (6.50 mL), followed by dropwise addition of ethanethiol (6.50 mL, 89.5 mmol, 2.40 equiv.). As this was added, the reaction mixture changed to a dark brown solution. The reaction mixture was stirred for 3 h at room temperature before it was neutralized by the addition of sat. aq. K_2CO_3 (30 mL). Then, the phases were separated, and the aq. phase was extracted with CH_2Cl_2 (3 × 50 mL). The combined organic phase was dried ($MgSO_4$), filtrated and the solvent removed in vacuo. The crude product was purified by flash chromatography (SiO_2, gradient elution, 2.5 – 5% MeOH in CH_2Cl_2) to afford the desired thioacetal **18** (6.29 g, 26.2 mmol, 70%) as a clear yellow oil. All spectroscopic and physical data were in agreement with those reported in the literature.[1] R_f (10% MeOH in CH_2Cl_2, visualized by $KMnO_4$ stain) = 0.57; $[\alpha]_D^{20}$ = -19.0 (*c* 1.0, MeOH) [Lit.[1] $[\alpha]_D^{20}$ = -19.2 (*c* 1.0, MeOH)]; ^{1}H NMR (400 MHz, $CDCl_3$): δ 4.09 – 4.04 (m, 2H), 3.83 – 3.70 (m, 2H), 3.61 (td, *J* = 5.2, 3.8 Hz, 1H), 2.78 – 2.56 (m, 4H), 2.29 (s, 2H), 2.22 (s, 1H), 2.03 – 1.99 (m, 2H), 1.30 – 1.24 (m, 6H); ^{13}C{^{1}H} NMR (100 MHz, $CDCl_3$): δ 73.8, 72.2, 63.6, 48.6, 38.9, 24.5, 24.2, 14.6 (2C).

(5*S*,6*R*)-5-(2,2-*Bis*(ethylthio)ethyl)-6-((*tert*-butyldimethylsilyl)oxy)-2,2,3,3,9,9,10,10-octamethyl-4,8-dioxa-3,9-disilaundecane (19)

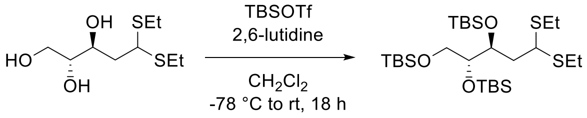

Triol **18** (5.09 g, 21.2 mmol, 1.00 equiv.) was dissolved in CH_2Cl_2 (96 mL) and cooled to -78 °C. The solution was stirred for 10 min before 2,6-lutidine (19.6 mL, 169 mmol, 8.00 equiv.) was added, followed by TBSOTf (19.4 mL, 84.5 mmol, 4.00 equiv.). The reaction mixture was allowed to slowly warm up to room temperature and stirred for 18 h. Sat. aq. NH_4Cl (65 mL) was added, the phases were separated, and the aqueous phase was extracted with CH_2Cl_2 (3 × 40 mL). The combined organic layer was washed with brine (30 mL), dried ($MgSO_4$), filtrated, and the solvent removed in vacuo. TBS-triol **19** (11.7 g, 20.8 mmol, 95%) was obtained after purification by flash chromatography (SiO_2, 1% EtOAc in heptane) as a clear, colorless oil. All spectroscopic and physical data were in agreement with those reported in the literature.[1] R_f (2.5% EtOAc in heptane, visualized by $KMnO_4$ stain) = 0.61; $[\alpha]_D^{20}$ = -9.4 (*c* 1.0, MeOH) [Lit.[1] $[\alpha]_D^{20}$ = -8.9 (c = 1.0, MeOH)]; ^{1}H NMR (400 MHz, $CDCl_3$): δ 4.17 (ddd, *J* = 9.5, 2.6, 1.4 Hz, 1H), 3.93 (dd, *J* = 11.0, 3.7 Hz, 1H), 3.69 (ddd, *J* = 7.2, 5.8, 1.4 Hz, 1H), 3.53 – 3.42 (m, 2H), 2.72 – 2.54 (m, 4H), 2.06 (ddd, *J* = 14.6, 9.4, 3.7 Hz, 1H), 1.79 (ddd, *J* = 14.6, 11.0, 2.6 Hz, 1H), 1.24 (td, *J* = 7.5, 6.3 Hz, 6H), 0.90 – 0.89 (m, 27H), 0.11 – 0.05 (m, 18H); ^{13}C{^{1}H} NMR (100 MHz, $CDCl_3$): δ 77.8, 72.2, 64.5, 48.2, 39.0, 26.2 (3C), 26.1 (3C), 26.1 (3C), 24.6, 23.2, 18.4, 18.4, 18.3, 14.8, 14.5, -2.8, -3.5, -4.4, -4.7, -5.3, -5.3.

(3*S*,4*R*)-3,4,5-Tris((*tert*-butyldimethylsilyl)oxy)pentanal (6)

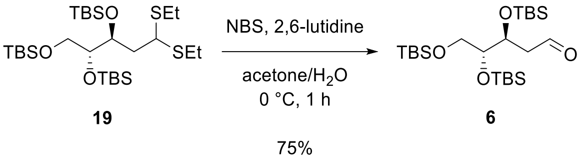

Compound **19** (5.48 g, 9.40 mmol, 1.00 equiv.) was dissolved in a mixture of acetone/water (64 mL/22 mL) and cooled to 0 °C, followed by addition of 2,6-lutidine (8.70 mL, 75.2 mmol, 8.00 equiv.) and NBS (13.4 g, 75.2 mmol, 8.00 equiv.). The reaction was stirred at 0 °C until completion as deemed by thin-layer chromatography. Then, the reaction mixture was added sat. aq. $Na_2S_2O_3$ (50 mL) and diluted with Et_2O (50 mL). The layers were separated, and the aq. phase was extracted with Et_2O (3 × 50 mL). The combined organic phase was washed with HCl (1.0 M, 20 mL), sat. aq. $NaHCO_3$ (20 mL) and brine (20 mL), dried ($MgSO_4$), filtrated and the solvent removed in vacuo. Aldehyde **6** (3.36 g, 7.05 mmol, 75%) was obtained after purification by flash chromatography (SiO_2, 1% EtOAc in hexane) as a clear, colorless oil. All spectroscopic and physical data were in agreement with those reported in the literature.[1] R_f (1% EtOAc in hexane, visualized by $KMnO_4$ stain) = 0.20; $[\alpha]_D^{20}$ = -6.0 (*c* 1.0, MeOH) [Lit.[1] $[\alpha]_D^{20}$ = -5.9 (*c* 1.0, MeOH)]; ^1H NMR (400 MHz, $CDCl_3$): δ 9.85 (dd, *J* = 3.4, 1.7 Hz, 1H), 4.35 (td, *J* = 5.2, 2.2 Hz, 1H), 3.77 (ddd, *J* = 7.7, 5.2, 2.2 Hz, 1H), 3.51 – 3.47 (m, 1H), 3.43 – 3.39 (m, 1H), 2.59 (ddd, *J* = 16.3, 5.5, 3.4 Hz, 1H), 2.46 (ddd, *J* = 16.3, 4.9, 1.7 Hz, 1H), 0.89 (m, 18H), 0.87 (s, 9H), 0.09 (s, 6H), 0.07 (s, 6H), 0.05 (s, 6H); $^{13}C\{^1H\}$ NMR (100 MHz, $CDCl_3$): δ 202.8, 77.3, 69.7, 64.5, 45.8, 26.0 (6C), 26.0 (3C), 18.4, 18.3, 18.2, -4.2, -4.4, -4.5, -4.8, -5.3, -5.4.

(5*S*,6*R*)-6-((*Tert*-butyldimethylsilyl)oxy)-2,2,3,3,9,9,10,10-octamethyl-5-((*Z*)-pent-2-en-1-yl)-4,8-dioxa-3,9-disilaundecane (21)

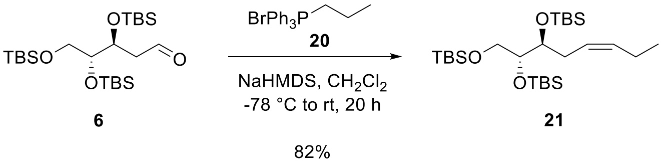

Wittig salt **20** (1.14 g, 2.95 mmol, 1.20 equiv.) was dissolved in dry CH_2Cl_2 (18 mL) and cooled to -78 °C in a dry ice/acetone bath. NaHMDS (0.6 M in toluene, 4.92 mL, 2.95 mmol, 1.20 equiv.) was added in a dropwise manner. Aldehyde **6** (1.17 g, 2.46 mmol, 1.00 equiv.) was dissolved in CH_2Cl_2 (3 mL) and added dropwise to the orange solution. As this was added, the reaction mixture turned yellow. The reaction was stirred for 6 h at -78 °C, and then allowed to slowly warm up to room temperature overnight. After completion, the reaction was added phosphate buffer (25 mL, pH = 7) and diluted with Et_2O (30 mL). The layers were separated, and the aqueous phase was extracted with Et_2O (3 × 30 mL). The combined organic phase was dried (Na_2SO_4), filtrated and the solvent removed in vacuo. The desired *Z*-alkene **21** (1.02 g, 2.09 mmol, 82%, *Z*/*E* >98:2) was obtained after purification by flash chromatography (SiO_2, hexane) as a clear, colorless oil. All spectroscopic and physical data were in agreement with those reported in the literature.[2] R_f (hexane, visualized by $KMnO_4$ stain) = 0.42; $[\alpha]_D^{25}$ = -5.3 (*c* 0.2, $CHCl_3$) [Lit.[2] $[\alpha]_D^{20}$ = -5.3 (*c* 0.2, $CHCl_3$)]; ^1H NMR (400 MHz, $CDCl_3$): δ 5.47 – 5.35 (m, 2H), 3.77 (ddd, *J* = 7.0, 5.5, 2.2 Hz, 1H), 3.69 (td, *J* = 6.0, 2.2 Hz, 1H), 3.62 (dd, *J* = 10.1, 6.2 Hz, 1H), 3.47 (dd, *J* = 10.1, 5.9 Hz, 1H), 2.32 – 2.17 (m, 2H), 2.08 – 1.99 (m, 2H), 0.95 (t, *J* = 7.5 Hz, 3H), 0.89 (s, 9H), 0.89 (s, 9H), 0.88 (s, 9H), 0.07 (s, 3H), 0.07 (s, 3H), 0.06 (s, 3H), 0.05 (s, 3H), 0.04 (s, 3H), 0.04 (s, 3H); $^{13}C\{^1H\}$ NMR (100 MHz, $CDCl_3$): δ 133.1, 126.3, 77.3, 74.7, 64.9, 30.8, 26.1 (6C), 26.1 (3C), 20.8, 18.5, 18.4, 18.3, 14.3, -4.1, -4.3, -4.4, -4.4, -5.2, -5.3.

(2*R*,3*S*,*Z*)-2,3-Bis((*tert*-butyldimethylsilyl)oxy)oct-5-en-1-ol (22)

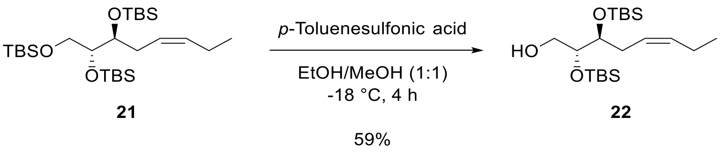

TBS-triol **21** (2.27 g, 4.52 mmol, 1.00 equiv.) was dissolved in a mixture of MeOH/EtOH (52 mL, 1:1) and cooled to -18 °C. *p*-Toluenesulfonic acid (PTSA, 860 mg, 4.52 mmol, 1.00 equiv.) was added, and the resulting reaction mixture was stirred for 4 h at the same temperature. Then the reaction was added a sat. aq. solution of $NaHCO_3$ (40 mL) and diluted with EtOAc (30 mL). The layers were separated, and the aqueous phase was extracted with EtOAc (3 × 30 mL). The combined organic phase was washed with brine (30 mL), dried ($MgSO_4$), and the solvent removed in vacuo. Alcohol **22** (1.04 g, 2.67 mmol, 59%, 90% brsm) was obtained after purification by flash chromatography (SiO_2, 5% EtOAc in hexane), and unreacted starting material was reisolated. All spectroscopic and physical data were in agreement with those reported in the literature.[2] R_f (5% EtOA in hexane, visualized by $KMnO_4$ stain) = 0.28; $[\alpha]_D^{25}$ = +2.8 (c = 0.1, MeOH) [Lit.[2] $[\alpha]_D^{20}$ = +2.9 (*c* 0.1, MeOH)]; ^{1}H NMR (400 MHz, $CDCl_3$): δ 5.55 – 5.34 (m, 2H), 3.80 – 3.71 (m, 2H), 3.65 – 3.58 (m, 2H), 2.36 – 2.22 (m, 2H), 2.08 – 2.00 (m, 2H), 0.96 (t, *J* = 7.6 Hz, 3H), 0.91 (s, 9H), 0.89 (s, 9H), 0.10 (s, 3H), 0.09 – 0.08 (m, 6H), 0.07 (s, 3H); ^{13}C{^{1}H} NMR (100 MHz, $CDCl_3$): δ 133.8, 124.7, 75.4, 74.8, 63.9, 32.1, 26.1 (3C), 26.0 (3C), 21.0, 18.3, 18.2, 14.2, -4.3, -4.3, -4.4, -4.5.

(2*S*,3*S*,*Z*)-2,3-Bis((*tert*-butyldimethylsilyl)oxy)oct-5-enal (9)

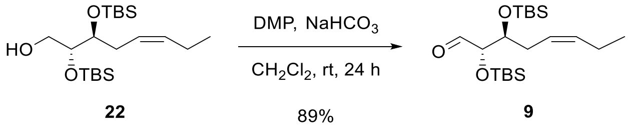

$NaHCO_3$ (1.02 g, 12.2 mmol, 5.70 equiv.) and Dess-Martin periodinane (DMP, 1.63 mg, 3.85 mmol, 1.80 equiv.) was added to a stirred solution of alcohol **22** (830 mg, 2.14 mmol, 1.00 equiv.) in CH_2Cl_2 (65.0 mL) at ambient temperature. The resulting suspension was stirred overnight and quenched by the addition of sat. aq. $Na_2S_2O_3$ (30 mL). The layers were separated, and the aqueous layer was extracted with CH_2Cl_2 (3 × 30 mL). The combined organic phase was washed with brine, dried (Na_2SO_4), filtrated, and the solvent removed in vacuo. The crude product was purified by flash chromatography (SiO_2, 2.5% EtOAc in hexane) to afford the desired aldehyde **9** (735 mg, 1.90 mmol, 89%) as a clear oil. All spectroscopic and physical data were in agreement with those reported in the literature.[2] R_f (5% EtOAc in hexane, visualized by $KMnO_4$ stain) = 0.48; $[\alpha]_D^{25}$ = +3.0 (*c* 0.5, $CHCl_3$) [Lit.[2] $[\alpha]_D^{20}$ = +3.0 (0.5, $CHCl_3$)]; ^{1}H NMR (400 MHz, $CDCl_3$): δ 9.56 – 9.55 (m, 1H), 5.53 – 5.43 (m, 1H), 5.29 – 5.21 (m, 1H), 3.95 – 3.89 (m, 2H), 2.47 – 2.39 (m, 1H), 2.24 – 2.18 (m, 1H), 2.11 – 1.98 (m, 2H), 0.96 (t, *J* = 7.5 Hz, 3H), 0.92 (s, 9H), 0.88 (s, 9H), 0.08 (s, 6H), 0.07 (s, 6H); ^{13}C{^{1}H} NMR (100 MHz, $CDCl_3$): δ 204.0, 135.1, 123.9, 81.1, 76.3, 31.7, 26.0 (3C), 26.0 (3C), 20.9, 18.4, 18.3, 14.2, -4.5, -4.5, -4.6, -4.7.

(2*E*,4*R*,5*S*,7*Z*)-4,5-Bis((*tert*-butyldimethylsilyl)oxy)deca-2,7-dienal (10)

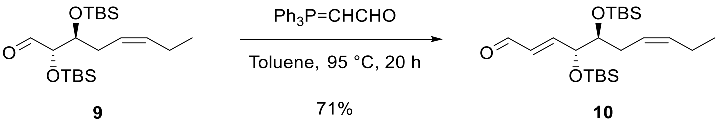

To a solution of aldehyde **9** (650 mg, 1.68 mmol, 1.00 equiv.) in toluene (33 mL) was added (triphenylphosphoranylidene)acetaldehyde (510 mg, 1.68 mmol, 1.00 equiv.). The suspension was warmed to 95 °C in an oil bath and stirred for 6 h at that temperature. Then another equivalent of (triphenylphosphoranylidene)acetaldehyde (510 mg, 1.68 mmol, 1.00 equiv.) was added, and the resulting suspension was warmed back to 95 °C and stirred for an additional 14 h. After cooling to room temperature, the solvent was evaporated, and the crude product was purified by flash chromatography (SiO_2, 2% EtOAc in hexane) to afford the α,β-unsaturated aldehyde **10** (490 mg, 1.19 mmol, 71%, 90% brsm, *E*/*Z* >98:2) as a yellow oil. Unreacted starting material was reisolated. R_f (5% EtOAc in hexane, visualized by UV and $KMnO_4$ stain) = 0.40; $[\alpha]_D^{25}$ = +13.2 (*c* 0.6, MeOH); ^{1}H NMR (400 MHz, $CDCl_3$): δ 9.58 (d, *J* = 8.0 Hz, 1H), 6.88 (dd, *J* = 15.7, 5.3 Hz, 1H), 6.24 (ddd, *J* = 15.7, 8.1, 1.5 Hz, 1H), 5.50 – 5.43 (m, 1H), 5.39 – 5.32 (m, 1H), 4.30 (ddd, *J* = 5.2, 3.6, 1.5 Hz, 1H), 3.74 (td, *J* = 6.2, 3.6 Hz, 1H), 2.32 – 2.20 (m, 2H), 2.07 – 1.99 (m, 2H), 0.96 (t, *J* = 7.5 Hz, 3H), 0.91 (s, 9H), 0.86 (s, 9H), 0.06 (s, 3H), 0.05 (s, 3H), 0.04 (s, 3H), 0.02 (s, 3H); ^{13}C{^{1}H} NMR (100 MHz, $CDCl_3$): δ 193.7, 157.7, 134.2, 132.5, 124.4, 75.4, 32.1, 26.0 (6C), 21.0, 18.4, 18.3, 14.2, -4.1, -4.3, -4.4, -4.6; HRMS (ESI): Exact mass calculated for $C_{22}H_{44}NaO_3Si_2$: 435.2721, found 435.2721.

(5*R*,6*S*)-5-((1*E*,3*E*)-4-Iodobuta-1,3-dien-1-yl)-2,2,3,3,8,8,9,9-octamethyl-6-((*Z*)-pent-2-en-1-yl)-4,7-dioxa-3,8-disiladecane (4)

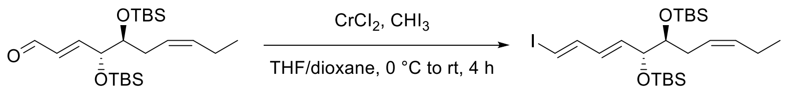

Vinyl iodide **4** was prepared according to the published procedure of Takai and coworkers.[3] $CrCl_2$ (119 mg, 0.969 mmol, 8.00 equiv.) was placed in a flask and dried under high vacuum using a heat gun. After cooling to room temperature, the flask was protected from light and dry THF (0.17 mL) and dioxane (2.95 mL) was added. The suspension was cooled to 0 °C before CHI_3 (119 mg, 0.303 mmol, 2.50 equiv.) was added in one portion. The resulting mixture was stirred at ambient temperature for 2 h, wherein the suspension turned from dark-green to red-brown. Then, the reaction mixture was cooled back to 0 °C, followed by dropwise addition of the α,β-unsaturated aldehyde **10** (50.0 mg, 0.121 mmol, 1.00 equiv.) dissolved in dioxane (0.21 mL). Stirring was continued for another 2 h at room temperature. When deemed complete, the reaction mixture was added sat. aq. NH_4Cl (2.5 mL), and the aqueous phase was extracted with Et_2O (3 × 3 mL). The combined organic layers were washed successively with a saturated aqueous solution of $Na_2S_2O_3$ (5 × 2 mL) and brine (5 × 2 mL), dried (Na_2SO_4), filtrated, and concentrated in vacuo. The crude product was purified by flash chromatography (SiO_2, hexane) to yield the desired product **4** (53 mg, 0.100 mmol, 82%) as a clear oil. R_f (hexane, visualized by UV and $KMnO_4$ stain) = 0.32; $[\alpha]_D^{20}$ = +1.1 (*c* 0.5, $CHCl_3$); ^{1}H NMR (400 MHz, $CDCl_3$): δ 7.04 (ddd, *J* = 14.4, 10.7, 0.8 Hz, 1H), 6.27 (d, *J* = 14.4 Hz, 1H), 6.04 (dd, *J* = 15.4, 10.6 Hz, 1H), 5.72 (dd, *J* = 15.5, 6.9 Hz, 1H), 5.46 – 5.31 (m, 2H), 3.98 (ddd, *J* = 7.1, 4.1, 1.1 Hz, 1H), 3.61 (td, *J* = 6.1, 4.0 Hz, 1H), 2.26 – 2.13 (m, 2H), 2.05 – 1.98 (m, 2H), 0.95 (t, *J* = 7.5 Hz, 1H), 0.88 (s, 9H), 0.86 (s , 9H), 0.03 - 0.02 (m, 9H), 0.00 (s, 3H); ^{13}C{^{1}H} NMR (100 MHz, $CDCl_3$): δ 145.1, 135.4, 133.5, 131.1, 125.3, 78.6, 76.7, 76.4, 31.8, 26.1 (3C), 26.1 (3C), 20.9, 18.4, 18.3, 14.3, -4.0, -4.0, -4.3, -4.5; HRMS (ESI): Exact mass calculated for $C_{23}H_{45}INaO_2Si_2$: 559.1895, found 559.1895. This compound is sensitive to heat, light and oxygen. Prudent care should be made during handling and storage. The above procedure was repeated on a 0.5 mmol scale of compound **10**.

7-Methoxy-7-oxoheptanoic acid (11)

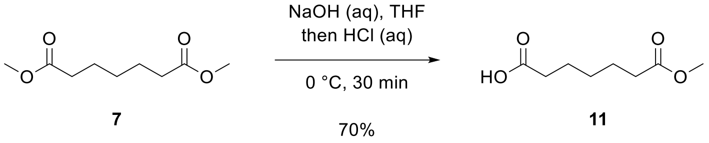

The selective monohydrolysis of diester **7** was performed according to the procedure of Niwayam.[4] Compound **7** (6.00 g, 31.8 mmol, 1.00 equiv.) was dissolved in THF (50 mL), and H_2O (500 mL) was added. The solution was cooled to 0 °C in an ice bath. To this mixture was added 0.25 M NaOH (200 mL) in small portions until detection of the dicarboxylic acid was observed by thin-layer chromatography. Then, the reaction was immediately acidified by addition of 1.0 M HCl (75 mL) at 0 °C, saturated with brine (100 mL), and extracted with EtOAc (3 × 200 mL). The combined organic layer was dried (Na_2SO_4), filtrated, and the solvent removed in vacuo. The crude product was purified by flash chromatography (SiO_2, hexane/EtOAc, 1:1) to afford the desired product **11** (3.90 g, 22.3 mmol, 70%) as a clear oil. R_f (60% EtOAc in hexane, visualized by bromocrestol green stain) = 0.30; ^{1}H NMR (400 MHz, CD_3OD): δ 4.87 (s, 1H), 3.65 (s, 3H), 2.31 (dt, J = 17.0, 7.4 Hz, 4H), 1.62 (pd, J = 7.5, 5.8 Hz, 4H), 1.40 – 1.32 (m, 2H); ^{13}C{^{1}H} NMR (100 MHz, CD_3OD): δ 177.5, 175.8, 52.0, 34.7, 34.6, 29.6, 25.7, 25.7; HRMS (ESI): Exact mass calculated for $C_8H_{14}NaO_4$: 197.0784, found 197.0784.

Methyl 7-oxo-9-(trimethylsilyl)non-8-ynoate (12)

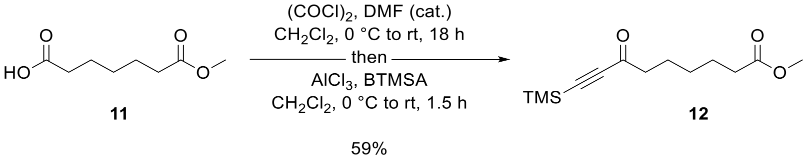

Ketone **12** was prepared according to the published procedure of Rodriguez and Spur.[5] Anhydrous *N,N*-dimethylformamide (30 µL, 1.95 x 10^{-3} mmol, 0.01 mol%) was added to a stirred solution of carboxylic acid **11** (3.30 g, 18.9 mmol, 1.00 equiv.) in anhydrous CH_2Cl_2 (51 mL). The reaction mixture was cooled to 0 °C, and oxalyl chloride (3.30 mL, 37.8 mmol, 2.00 equiv.) was added in a dropwise manner. The resulting solution was allowed to slowly warm to room temperature and stirred overnight. After removal of the volatile compounds by reduced pressure, the crude product was dissolved in anhydrous CH_2Cl_2 (24 mL) and transferred to a flame-dried flask with *bis*(trimethylsilyl)acetylene (3.21 g, 18.9 mmol, 1.00 equiv.) under argon. The resulting solution was added in a dropwise manner to a stirred solution of $AlCl_3$ (3.27 g, 24.6 mmol, 1.30 equiv.) in anhydrous CH_2Cl_2 (24 mL) at 0 °C. The resulting reaction mixture was stirred at 0 °C for 30 min, warmed up to room temperature over a period of 45 min and then cooled back down to 0 °C. Then, the reaction mixture was added 1 M HCl (50 mL) and stirred for an additional 10 min. The resulting thick suspension was vacuum-filtrated through a short plug of silica gel directly into a separatory funnel and the plug was washed with additional fresh CH_2Cl_2 (50 mL). The phases were separated and the aqueous phase was extracted with CH_2Cl_2 (3 × 50 mL). The combined organic phase was dried (Na_2SO_4) and concentrated in vacuo. The crude product was purified by flash chromatography (SiO_2, 10% EtOAc in hexane) to yield the desired product **12** (2.85 g, 11.2 mmol, 59%) as a colorless oil. The spectroscopic data were in agreement with those reported in literature.[5] R_f (10% EtOAc in hexane, visualized by UV and $KMnO_4$ stain) = 0.25; ^{1}H NMR (400 MHz, $CDCl_3$): δ 3.66 (s, 3H), 2.56 (td, J = 7.3, 2.6 Hz, 2H), 2.31 (t, J = 7.5 Hz, 2H), 1.69 – 1.62 (m, 4H), 1.38 – 1.30 (m, 2H), 0.23 (s, 9H); ^{13}C{^{1}H} NMR (100 MHz, $CDCl_3$): δ 187.8, 174.2, 102.1, 97.9, 51.6, 45.1, 33.9, 28.5, 24.7, 23.6, -0.6 (3C); HRMS (ESI): Exact mass calculated for $C_{13}H_{22}NaO_3Si$: 277.1230, found 277.1230.

Methyl (*S*)-7-hydroxy-9-(trimethylsilyl)non-8-ynoate ((*S*)-13)

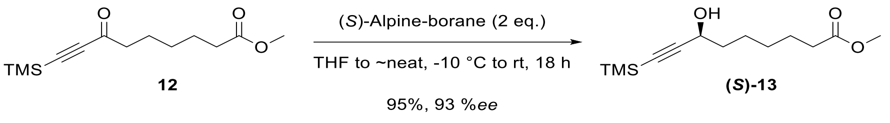

Ketone **12** (1.80 g, 7.08 mmol, 1.00 equiv.) was azeotropically dried with 2-methyltetrahydrofuran (2 × 2 mL) and then placed under high vacuum for 30 min. The flask was vented with argon and cooled to -10 °C. (*S*)-Alpine-borane solution (28.0 mL, 0.5 M in THF, 14.2 mmol, 2.00 equiv.) was added in one portion, and most of the THF solvent was immediately removed in vacuo with efficient stirring while warming up to 0° C. The resulting, highly viscous reaction mixture was then allowed to warm to ambient temperature and stirred overnight. Next, the reaction mixture was cooled to 0 °C and acetaldehyde (0.80 mL, 620 mg, 0.785 g/mL at 25 °C, 2.00 equiv.) was added dropwise. After 15 min, Et_2O (28.0 mL) was added, followed by dropwise addition of ethanolamine (1.72 mL, 1.72 g, 1.012 g/mL, 4.00 equiv.). The reaction was stirred for 30 min at 0 °C, then warmed to room temperature and stirred for an additional 1 h. The white, solid 9-BBN/ethanolamine complex was removed by filtration and the filtrate was washed with water (2 × 20 mL). The organic phase was dried (Na_2SO_4), filtrated, concentrated in vacuo and then purified by flash chromatography (SiO_2, gradient elution, 10 – 20% EtOAc in hexane) to give the desired product (*S*)-**13** (1.72 mg, 6.72 mmol, 95%, 93% *ee* (see below)) as a clear oil. All spectroscopic data were in agreement with those reported in literature.[5] R_f (20% EtOAc in hexane, visualized by $KMnO_4$ stain) = 0.29; $[\alpha]_D^{20}$ = -0.56 (*c* 1.3, $CHCl_3$) [Lit.[5] $[\alpha]_D^{20}$ = -0.50 (*c* 0.63, $CHCl_3$)]; ^1H NMR (400 MHz, CD_3OD): δ 4.27 (t, *J* = 6.7 Hz, 1H), 3.65 (s, 3H), 2.33 (t, *J* = 7.4 Hz, 2H), 1.67 – 1.59 (m, 4H), 1.49 – 1.42 (m, 2H), 1.40 – 1.34 (m, 2H), 0.15 (s, 9H); $^{13}C\{^1H\}$ NMR (100 MHz, CD_3OD): δ 175.9, 108.8, 88.8, 63.0, 52.0, 38.6, 34.7, 29.7, 26.0, 25.9, -0.1 (3C); HRMS (ESI): Exact mass calculated for $C_{13}H_{24}NaO_3Si$: 279.1387, found 279.1386.

Methyl 7-hydroxy-9-(trimethylsilyl)non-8-ynoate (rac 13)

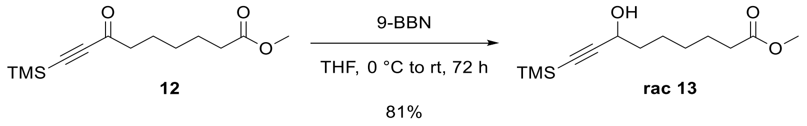

Ketone **12** (50 mg, 0,197 mmol, 1.00 equiv.) was azeotropically dried with 2-methyltetrahydrofuran (2 × 1 mL) and then placed under high vacuum for 30 min. The flask was cooled to 0 °C and 9-borabicyclo[3.3.1]nonane solution (9-BBN, 0.80 mL, 0.5 M in THF, 0.393 mmol, 2.00 equiv.) was added. Approximately half the solvent volume was removed under vacuum at room temperature, and the reaction was stirred for 72 h. Acetaldehyde (11 µL, 0.197 mmol, 1.00 equiv.) was added in a dropwise manner and the reaction was allowed to stir for an additional 1 h. The reaction mixture was diluted with Et_2O (1.1 mL), then ethanolamine (12 µL, 0.197 mmol, 1.00 equiv.) was added. After 30 min, the suspension was concentrated in vacuo. H_2O (2 mL) was added, and the aqueous phase was extracted with Et_2O (3 × 2 mL). The combined organic phases were dried (Na_2SO_4), filtrated and concentrated in vacuo. The crude product was purified by flash chromatography (SiO_2, gradient elution, 10 - 20% EtOAc in hexane) to give racemic **13** (41 mg, 0.160 mmol, 81%) as a clear oil. The obtained spectral data matched those given for compound (*S*)-**13**.

(*S*)-9-Methoxy-9-oxo-1-(trimethylsilyl)non-1-yn-3-yl 2-naphthoate ((*S*)-23)

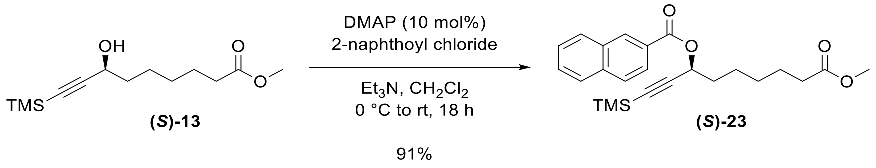

The propargylic alcohol (*S*)-**13** (30 mg, 0.117 mmol, 1.00 equiv.) was dissolved in CH_2Cl_2 (0.50 mL) and cooled to 0 °C. Triethylamine (0.049 mL, 0.327 mmol, 3.00 equiv.) was added dropwise followed by 4-(dimethylamino)pyridine (DMAP, 1.40 mg, 0.0117 mmol, 10 mol%). Next, 2-naphthoyl chloride (27 mg, 0.140 mmol, 1.20 equiv.) was added, and the resulting suspension was allowed to slowly warm up to room temperature and stirred overnight. The solvent was removed under a gentle stream of argon, and then hexane (2.5 mL) and phosphate buffer (2.5 mL) was added. The phases were separated after 5 min of stirring, and the aqueous phase was extracted in the same manner two more times. The combined organic phase was dried (Na_2SO_4), filtrated and the solvent removed in vacuo. The material thus obtained was purified by flash chromatography (SiO_2, gradient elution, 0 – 10% EtOAc in hexane) to afford the desired compound (*S*)-**23** (44 mg, 0.107 mmol, 91%) as a clear oil. The enantiomeric excess was determined by chiral HPLC analysis (Chiralpak AD-H, hexanes/*i*PrOH 99/1, 1 mL/min): t_r (e_1, minor) = 10.89 min and t_r (e_2, major) = 11.76 min. R_f (10% EtOAc in hexane, visualized by UV and $KMnO_4$ stain) = 0.32; $[\alpha]_D^{20}$ = +15.0 (*c* 0.7, $CHCl_3$); ^1H NMR (400 MHz, $CDCl_3$): δ 8.64 – 8.63 (m, 1H), 8.08 (dd, *J* = 8.6, 1.7 Hz, 1H), 7.97 (dd, *J* = 8.1, 1.5 Hz, 1H), 7.89 (dd, *J* = 8.5, 2.2 Hz, 2H), 7.58 (dddd, *J* = 19.7, 8.2, 6.9, 1.4 Hz, 2H), 5.70 (t, *J* = 6.6 Hz, 3H), 3.66 (s, 3H), 2.33 (t, *J* = 7.5, 2H), 1.98 – 1.91 (m, 2H), 1.78 (p, *J* = 7.5 Hz, 2H), 1.61 – 1.54 (m, 2H), 1.46 – 1.40 (m, 2H), 0.19 (s, 9H); $^{13}C\{^1H\}$ NMR (100 MHz, $CDCl_3$): δ 174.3, 165.8, 135.8, 132.6, 131.5, 129.6, 128.5, 128.3, 127.9, 127.4, 126.8, 125.5, 102.8, 90.8, 65.1, 51.6, 34.9, 34.1, 28.8, 24.9, -0.02 (3C); HRMS (ESI): Exact mass calculated for $C_{24}H_{30}NaO_4Si$: 433.1806, found 433.1806.

The exact same procedure was followed for racemic **13**. Yield: 56 mg (93%). The obtained spectral data matched those given for compound (*S*)-**23**.

Methyl (*S*)-7-((*tert*-butyldimethylsilyl)oxy)-9-(trimethylsilyl)non-8-ynoate (24)

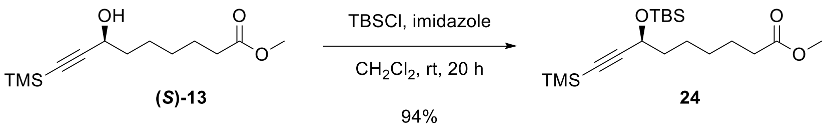

Imidazole (720 mg, 10.3 mmol, 2.00 equiv.) and *tert*-butyldimethylsilyl chloride (930 mg, 6.18 mmol, 1.50 equiv.) were added in a successive manner to a stirred solution of propargylic alcohol (*S*)-**13** (1.32 g, 5.16 mmol, 1.00 equiv.) in CH_2Cl_2 (15 mL) at room temperature. The reaction was stirred overnight and quenched by the addition of a half-sat. aq. solution of NaH_2PO_4 (40 mL). The phases were separated, and the aqueous phase was extracted with CH_2Cl_2 (3 × 40 mL). The combined organic phases were dried (Na_2SO_4), filtrated, and the solvent removed in vacuo. The desired product **24** (1.79 mg, 4.85 mmol, 94%) was obtain as a clear oil after purification by flash chromatography (SiO_2, 2.5% EtOAc in hexane). All spectroscopic data were in agreement with those reported in literature.[5] R_f (5% EtOAc in hexane, visualized by UV and $KMnO_4$ stain) = 0.32; $[\alpha]_D^{20}$ = -36.3 (*c* 0.4, $CHCl_3$) [Lit.[5] $[\alpha]_D^{25}$ = -37.0 (*c* 0.6, $CHCl_3$)]; ^1H NMR (400 MHz, $CDCl_3$): δ 4.30 (t, *J* = 6.6 Hz, 1H), 3.65 (s, 3H), 2.30 (t, *J* = 7.5 Hz, 2H), 1.67 – 1.58 (m, 4H), 1.47 – 1.30 (m, 4H), 0.89 (s, 9H), 0.14 (s, 9H), 0.12 (s, 3H), 0.09 (s, 3H); $^{13}C\{^1H\}$ NMR (100 MHz, $CDCl_3$): δ 174.3, 107.9, 88.6, 63.4, 51.6, 38.4, 34.1, 28.9, 26.0 (3C), 25.0, 25.0, 18.4, -0.03 (3C), -4.3, -4.8; HRMS (ESI): Exact mass calculated for $C_{19}H_{38}NaO_3Si_2$: 393.2252, found 393.2251.

Methyl (*S*)-7-((*tert*-butyldimethylsilyl)oxy)non-8-ynoate (5)

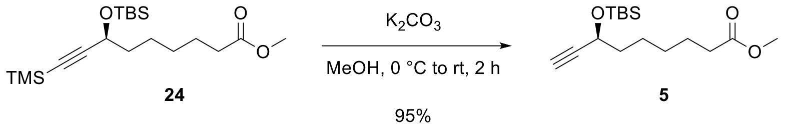

TMS-protected alkyne **24** (1.50 g, 4.05 mmol, 1.00 equiv) was dissolved in methanol (39 mL) and cooled to 0 °C. K_2CO_3 (570 mg, 4.05 mmol, 1.00 equiv.) was added in one portion, and the reaction was warmed to room temperature. The reaction was followed with TLC analysis (5% EtOAc in hexane) until completion (the product can be observed just below the starting material). After 2 h of stirring, the reaction mixture was cooled back to 0 °C, quenched by the addition of phosphate buffer (45 mL, pH = 7) and stirred for an additional 5 min. NaCl (~3 g) was added, and the aqueous phase was extracted with hexane (5 × 45 mL). The combined organic phase was dried ($MgSO_4$), filtrated and concentrated in vacuo. The crude product was purified by flash chromatography (SiO_2, 5% EtOAc in hexane) to give the desired compound **5** (1.15 g, 3.85 mmol, 95%) as a clear oil. All spectroscopic data were in agreement with those reported in literature.[5] R_f (5% EtOAc in hexane, visualized by $KMnO_4$ stain) = 0.25; $[\alpha]_D^{20}$ = -34.9 (*c* 1.5, $CHCl_3$) [Lit.[5] $[\alpha]_D^{20}$ = -36.0 (*c* 0.5, $CHCl_3$)]; ^{1}H NMR (400 MHz, $CDCl_3$): δ 4.33 (td, *J* = 6.4, 2.1 Hz, 1H), 3.66 (s, 3H), 2.36 (d, *J* = 2.1 Hz, 1H), 2.30 (t, *J* = 7.5 Hz, 3H), 1.70 – 1.60 (m, 4H), 1.49 – 1.40 (m, 2H), 1.37 – 1.32 (m, 2H), 0.89 (s, 9H), 0.13 (s, 3H), 0.10 (s, 3H); ^{13}C{^{1}H} NMR (100 MHz, $CDCl_3$): δ 174.3, 85.7, 72.1, 62.8, 51.6, 38.5, 34.1, 28.9, 25.9 (3C), 25.0, 24.9, 18.4, -4.4, -4.9; HRMS (ESI): Exact mass calculated for $C_{16}H_{30}NaO_3Si$: 321.1856, found 321.1856.

Methyl (*S*,*E*)-7-((*tert*-butyldimethylsilyl)oxy)-9-(tributylstannyl)non-8-enoate (25)

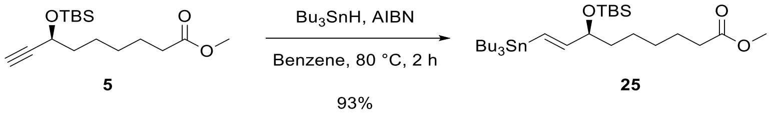

Compound **25** was prepared by following the protocol of Sulikowski and coworkers.[6] Alkyne **5** (980 mg, 3.29 mmol, 1.00 equiv.) was dissolved in benzene (68 mL). Bu_3SnH (2.70 mL, 10.1 mmol, 3.00 equiv.) and AIBN (90.0 mg, 0.550 mmol, 16 mol%) was added. The reaction was heated to 80 °C with stirring. After 2 h, the reaction mixture was cooled back to room temperature and the solvent was removed in vacuo. The crude product was purified by flash chromatography (SiO_2, 2.5% EtOAc in hexane) to obtain the stannane product **25** (1.80 g, 3.06 mmol, 93%) as a clear oil. R_f (5% EtOAc in hexane, visualized by $KMnO_4$ stain) = 0.48; $[\alpha]_D^{20}$ = -12.5 (*c* 1.6, $CHCl_3$); ^{1}H NMR (400 MHz, $CDCl_3$): δ 6.00 (d, *J* = 19.0 Hz, 1H), 5.89 (dd, *J* = 19.0, 5.6 Hz, 1H), 4.00 (q, *J* = 5.9 Hz, 1H), 3.66 (s, 3H), 2.29 (t, *J* = 7.6 Hz, 2H), 1.64 – 1.25 (m, 26H), 0.90 – 0.86 (m, 18H), 0.03 (s, 3H), 0.02 (s, 3H); ^{13}C{^{1}H} NMR (100 MHz, $CDCl_3$): δ 174.4, 152.0, 126.6, 51.6, 38.0, 34.2, 29.3, 29.3 (3C), 27.4 (3C), 26.1 (3C), 25.2, 25.1, 18.5, 13.9 (3C), 9.6 (3C), -4.1, -4.6; HRMS (ESI): Exact mass calculated for $C_{28}H_{58}NaO_3Si^{116}Sn$: 609.3065, found 609.3064.

Methyl (*S*,*E*)-7-((*tert*-butyldimethylsilyl)oxy)-9-iodonon-8-enoate (14)

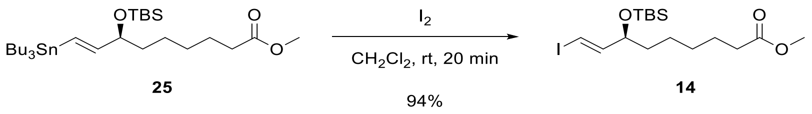

Stannane **25** (1.66 g, 2.82 mmol, 1.00 equiv.) was dissolved in dry CH_2Cl_2 (8.0 mL). A solution of I_2 (1.07 g, 4.21 mmol, 1.50 equiv.) in dry CH_2Cl_2 (11 mL) was added dropwise at room temperature until the resulting solution maintained a pink color. The reaction was stirred for 20 min, followed by addition of saturated aqueous $Na_2S_2O_3$ (5 mL), H_2O (5 mL), and saturated aqueous $NaHCO_3$ (5 mL). After stirring for 5 min, the phases were separated and the aqueous phase was extracted with CH_2Cl_2 (3 × 20 mL). The combined organic phases were dried ($MgSO_4$), filtrated, and the solvent removed in vacuo. The crude product was purified by flash column chromatography (SiO_2, 5% Et_2O in hexane) to obtain the vinyl iodide **14** (1.13 g, 2.65 mmol, 94%) as a clear oil. All spectroscopic and physical data were in agreement with those previously reported.[5] R_f (5% Et_2O in hexane, visualized by UV and $KMnO_4$ stain) = 0.26; $[\alpha]_D^{20}$ = -17.7 (*c* 0.9, $CHCl_3$) [Lit.[5] $[\alpha]_D^{20}$ = -18.5 (*c* 0.4, $CHCl_3$)]; ^1H NMR (400 MHz, $CDCl_3$): δ 6.49 (dd, *J* = 14.4, 6.0 Hz, 1H), 6.20 – 6.16 (m, 1H), 4.05 (qd, *J* = 6.0, 1.3 Hz, 1H), 3.66 (s, 3H), 2.29 (t, *J* = 7.5 Hz, 2H), 1.65 – 1.59 (m, 2H), 1.50 – 1.42 (m, 2H), 1.36 – 1.28 (m, 4H), 0.88 (m, 9H), 0.03 (s, 3H), 0.02 (s, 3H); $^{13}C\{^1H\}$ NMR (100 MHz, $CDCl_3$): δ 174.3, 149.4, 75.8, 75.2, 51.6, 37.4, 34.2, 29.2, 26.0 (3C), 25.0, 24.6, 18.4, -4.4, -4.7; HRMS (ESI): Exact mass calculated for $C_{16}H_{31}INaO_3Si$: 449.0979, found 449.0979. This compound is sensitive to heat, light and oxygen and prudent care should be made when handling and storing it.

Methyl (*S*,*E*)-7-((*tert*-butyldimethylsilyl)oxy)undec-8-en-10-ynoate (3)

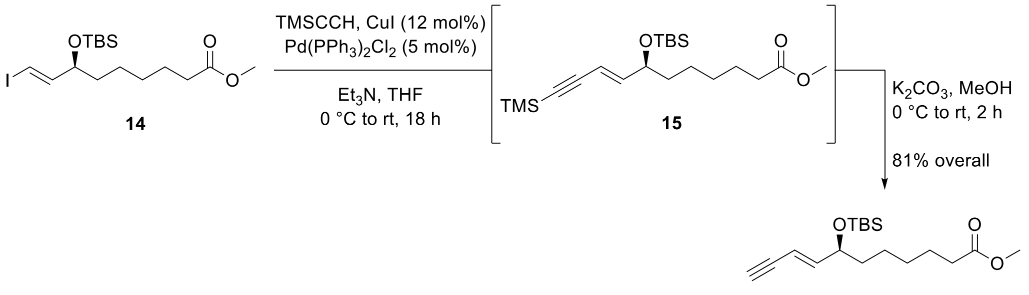

Vinyl iodide **14** (1.06 g, 2.49 mmol, 1.00 equiv.) was dissolved in dry THF (14.0 mL) and cooled to 0 °C. $Pd(PPh_3)_2Cl_2$ (90 mg, 0.12 mmol, 5 mol%) and CuI (57 mg, 0.30 mmol, 12 mol%) was added in one portion followed by dropwise addition of Et_3N (0.50 g, 0.70 mL, 4.98 mmol, 2.00 equiv.). Ethynyltrimethylsilane (62 mg, 0.90 mL, 6.23 mmol, 2.50 equiv.) was dissolved in a minimum amount of dry THF and added in a dropwise manner. The reaction mixture was allowed to slowly warm up to room temperature and stirred in the dark for 18 h. After completion, the suspension was filtrated through a plug of silica gel (10% EtOAc in hexane). The solvent was removed in vacuo, and the crude product thus obtained was used directly in the next reaction without any further purification. R_f (5% EtOAc in hexane, visualized by UV and $KMnO_4$ stain) = 0.26.

The crude TMS-acetylene **15** was dissolved in methanol (22 mL) and cooled to 0 °C. K_2CO_3 (690 mg, 4.99 mmol, 2.00 equiv.) was added in one portion, and the reaction was warmed to room temperature. The reaction was followed with TLC analysis (10% EtOAc in hexane) until completion (the product can be observed just below the starting material). After 2 h of stirring, the reaction mixture was cooled back to 0 °C, quenched by the addition of phosphate buffer (30 mL, pH = 7) and stirred for an additional 5 min. NaCl (1.80 g) was added, and the aqueous phase was extracted with hexane (5 × 30 mL). The combined organic phases were dried (Na_2SO_4), filtrated and concentrated in vacuo. The crude product was purified by flash chromatography (SiO_2, 5% EtOAc in hexane) to give the desired compound **3** (655 mg, 2.02 mmol, 81% over two steps) as a clear oil. R_f (5% EtOAc in hexane, visualized by $KMnO_4$ stain) = 0.15; $[\alpha]_D^{20}$ = -7.3 (*c* 0.3, MeOH); ^1H NMR (400 MHz, $CDCl_3$): δ 6.24 – 6.18 (ddd, *J* = 15.9, 5.3, 0.6 Hz, 1H), 5.65 – 5.60 (ddd, *J* = 15.9, 2.3, 1.6 Hz, 1H), 4.18 4.14 (qd, *J* = 5.8, 1.6 Hz, 1H), 3.67 (s, 3H), 2.87 – 2.86 (d, *J* = 2.3 Hz, 1H), 2.32 – 2.28 (t, *J* = 7.5 Hz, 2H), 1.65 – 1.58 (m, 2H), 1.51 – 1.44 (m, 2H), 1.36 – 1.27 (m, 4H), 0.89 (s, 9H), 0.04 (s, 3H), 0.03 (s, 3H); $^{13}C\{^1H\}$ NMR (100 MHz, $CDCl_3$): δ

174.3, 148.6, 107.7, 82.2, 77.4, 72.3, 51.6, 37.6, 34.2, 29.3, 26.0 (3C), 25.0, 24.6, 18.3, -4.4, -4.7; HRMS (ESI): Exact mass calculated for $C_{18}H_{32}NaO_3Si$: 347.2013, found 347.2013.

Methyl (7*S*,8*E*,12*E*,14*E*,16*R*,17*S*,19*Z*)-7,16,17-tris((*tert*-butyldimethylsilyl)oxy)docosa-8,12,14,19-tetraen-10-ynoate (16)

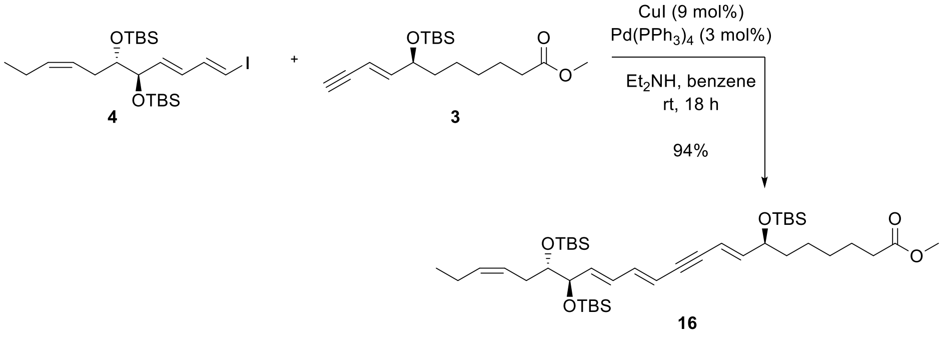

To a stirred solution of vinyliodine **4** (120 mg, 0.224 mmol, 1.00 equiv.) in benzene (0.4 mL) and Et_2NH (0.96 mL) was added $Pd(PPh_3)_4$ (7.8 mg, 6.8 µmol, 3 mol%) at ambient temperature and the reaction mixture was stirred for 30 min in the dark. CuI (4.0 mg, 21 µmol, 9 mol%) and a solution of alkyne **3** (72 mg, 0.224 mmol, 1.00 equiv.) in a minimum amount of Et_2NH were added in a sequential manner and the resulting suspension was stirred overnight. After completion, the reaction mixture was quenched by the addition of sat. aq. NH_4Cl (6 mL) and diluted with Et_2O (6 mL). The layers were separated, and the aqueous layer was extracted (Et_2O, 3 × 6 mL). The combined organic phase was washed with brine (6 mL), dried over $MgSO_4$, filtrated, and the solvent removed in vacuo. Alkyne **16** (156 mg, 0.212 mmol, 94%) was obtained as a pale yellow oil after purification by flash column chromatography (SiO_2, 1% EtOAc in hexane). R_f (10% EtOAc in hexane, visualized by UV and $KMnO_4$ stain) = 0.50; $[\alpha]_D^{20}$ = -15.5 (*c* 1.1, MeOH); ^{1}H NMR (400 MHz, C_6D_6): δ 6.65 (dd, *J* = 15.4, 10.8 Hz, 1H), 6.22 – 6.15 (m, 2H), 5.98 (dt, *J* = 15.8, 1.9 Hz, 1H), 5.82 – 5.73 (m, 2H), 5.59 – 5.46 (m, 2H), 4.10 (dd, *J* = 6.9, 4.5 Hz, 1H), 3.72 (td, *J* = 5.8, 4.4, Hz, 1H), 3.36 (s, 3H), 2.40 (t, *J* = 6.1 Hz, 2H), 2.11 – 2.02 (m, 4H), 1.55 – 1.47 (m, 2H), 1.42 – 1.32 (m, 2H), 1.27 – 1.18 (m, 2H), 1.17 – 1.09 (m, 2H), 1.00 (s, 9H), 0.99 (s, 9H), 0.96 (s, 9H), 0.94 (t, *J* = 7.5 Hz, 3H), 0.10 (s, 3H), 0.09 (s, 6H), 0.07 (s, 3H), 0.05 (s, 3H), 0.03 (s, 3H); $^{13}C\{^1H\}$ NMR (100 MHz, C_6D_6): δ 173.3, 146.6, 141.2, 137.2, 133.6, 131.4, 125.6, 112.0, 109.7, 91.5, 90.2, 77.1, 76.9, 72.9, 50.9, 38.0, 34.0, 32.0, 29.4, 26.2 (6C), 26.1 (3C), 25.2, 25.0, 21.3, 18.5, 18.4 (2C), 14.4, -3.8, -3.9, -4.1, -4.3, -4.5, -4.7; HRMS (ESI): Exact mass calculated for $C_{41}H_{76}NaO_5Si_3$: 755.4893, found 755.4892.

Methyl (7*S*,8*E*,12*E*,14*E*,16*R*,17*S*,19*Z*)-7,16,17-trihydroxydocosa-8,12,14,19-tetraen-10-ynoate (17)

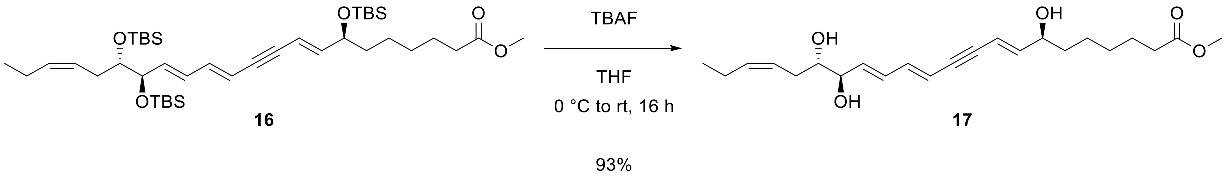

TBS-protected triol **16** (81.0 mg, 0.110 mmol, 1.00 equiv.) was dissolved in THF (1.8 mL) and cooled to 0 °C. TBAF (1 M in THF, 0.828 mL, 0.828 mmol, 7.50 equiv.) was added dropwise, and the resulting mixture was stirred over night. After completion, the reaction mixture was quenched by the addition of phosphate buffer (pH = 7, 1.5 mL). Brine (7.5 mL) and EtOAc (9 mL) was added, and the phases were separated. The aqueous phase was extracted with EtOAc (3 × 9 mL), and the combined organic layer was dried (Na_2SO_4), filtrated and the solvent removed in vacuo. The crude product was purified by flash chromatography (SiO_2, EtOAc/heptane 1:1) to obtain the desired triol **17** (40.2 mg, 0.103 mmol, 93%) as a clear oil. R_f (EtOAc/Heptane 1:1, visualized by UV and $KMnO_4$ stain) = 0.19; $[\alpha]_D^{20}$ = +40.0 (*c* 0.9, MeOH); ^{1}H NMR (400 MHz, CD_3OD): δ 6.58 (dd, *J* = 15.4, 10.8 Hz, 1H), 6.35 (dd, *J* = 15.6, 10.7 Hz, 1H), 6.07 (dd, *J* = 15.8, 6.1 Hz, 1H), 5.92 (dd, *J* = 15.3, 6.6 Hz, 1H), 5.82 (ddd, *J* = 15.8, 2.4, 1.4 Hz, 1H), 5.75 (dd, *J* = 15.4, 2.4 Hz, 1H), 5.53 – 5.39 (m, 2H), 4.08 (qd, *J* = 6.2, 1.4 Hz, 1H), 4.00 (ddd, *J* = 6.5, 5.0, 1.2 Hz, 1H), 3.52 (dt, *J* = 8.2, 4.9 Hz, 1H), 2.33 (t, *J* = 7.4 Hz, 2H), 2.19 – 2.11 (m, 1H), 2.10 – 2.01 (m, 2H), 1.62 (p, *J* = 7.4 HZ, 1H), 1.54 – 1.47 (m, 2H), 1.41 – 1.29 (m, 4H), 0.97 (t, *J* = 7.5 Hz, 3H); ^{13}C{^{1}H} NMR (100 MHz, CD_3OD): δ 176.0, 147.1, 142.2, 136.7, 134.4, 132.3, 126.2, 112.3, 110.5, 91.3, 90.0, 76.0, 75.9, 72.7, 52.0, 37.8, 34.7, 31.8, 30.1, 26.1, 25.9, 21.7, 14.6; HRMS (ESI): Exact mass calculated for $C_{23}H_{34}NaO_5$: 413.2298, found 413.2297.

RvD2$_{n\text{-}3\ DPA}$ methyl ester (2)

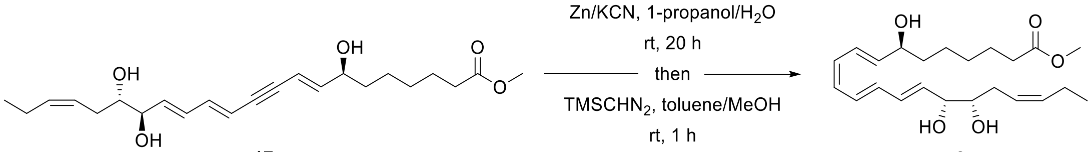

17 **2**

59%

Compound **17** was prepared by following the protocol reported by Näf *et al.*[7] Compound **17** (20.0 mg, 51.2 µmol, 1.00 equiv.) was dissolved in a 1:1 mixture of 1-propanol/H_2O (2.4 mL, pH = 7.0). Zinc powder (730 mg, 11.2 mmol, 218 equiv.) and KCN (47.0 mg, 0.722 mmol, 14.1 equiv.) was added in a successive manner, and the resulting suspension was stirred at ambient temperature under protection of argon in the dark. After stirring for 20 hours, the reaction mixture was filtered through a short pad of Celite® and the pad was washed with EtOAc (10 mL). Sat aq. NH_4Cl (8 mL) was added to the filtrate and the phases were separated. The aqueous phase was extracted with EtOAc (3 × 6 mL), and the combined organic layer was concentrated in vacuo, but not to complete dryness [R_f (10% MeOH in CH_2Cl_2, visualized by UV and $KMnO_4$ stain) = 0.26]. The crude product was immediately dissolved in a mixture of toluene/MeOH (3:2, 0.92 mL), followed by dropwise addition of TMS-diazomethane (2.0 M in Et_2O, 33.3 µL, 66.6 µmol, 1.30 equiv.) until the yellow color persisted. After completion (~1 h), the reaction was quenched with sat. aq. NH_4Cl (2 mL). The layers were separated, and the aq. phase was extracted with Et_2O (3 × 2 mL). The combined organic layer was evaporated, and the crude product was purified by flash column chromatography (SiO_2, gradient elution, 2 – 5% MeOH in CH_2Cl_2) to afford RvD2$_{n\text{-}3\ DPA}$ methyl ester **2** (12.5 mg, 31.8 µmol, 59%) as a clear oil. The chemical purity (>96%) was determined by HPLC analysis (Eclipse XDBC$_{18}$, MeOH/H_2O 70:30, 1.0 mL/min): t_r (major) = 11.16 min, t_r (minor) = 12.10 min. R_f (5% MeOH in CH_2Cl_2, visualized by UV and $KMnO_4$ stain) = 0.26; $[\alpha]_D^{20}$ = +23.8 (*c* 0.3, MeOH); ^{1}H NMR (400 MHz, CD_3OD): δ 6.76 (ddd, *J* = 14.2, 9.9, 3.4 Hz, 2H), 6.39 (dd, *J* = 14.8, 11.5 Hz, 1H), 6.30 (dd, *J* = 14.3, 10.8 Hz, 1H), 6.06 – 5.98 (m, 2H), 5.85 (ddd, *J* = 14.9, 12.0, 5.3 Hz, 1H), 5.72 (dd, *J* = 15.1, 6.6 Hz, 1H), 5.60 – 5.45 (m, 2H), 4.15 (q, *J* = 6.4 Hz, 1H), 4.04 – 3.97 (m, 1H), 3.66 (s, 3H), 3.56 – 3.49 (m, 1H), 2.34 (t, *J* = 7.4 Hz, 2H), 2.27 – 2.10 (m, 2H), 2.04 (p, *J* = 7.3 Hz, 2H), 1.65 – 1.61 (m, 2H), 1.58 – 1.47 (m, 2H), 1.42 – 1.30 (m, 4H), 1.00 (t, *J* = 7.6 Hz, 3H); ^{13}C{^{1}H} NMR (100 MHz, CD_3OD): δ 176.0, 138.8, 135.6, 134.6, 134.5, 133.4, 130.4, 130.3, 129.1, 126.6, 126.5, 76.2, 75.9, 73.1, 52.0, 38.2, 37.3, 34.7, 30.1, 26.7, 26.2, 26.0, 14.2; HRMS

(ESI): Exact mass calculated for $C_{23}H_{36}NaO_5$: 415.2455, found 415.2456; UV (MeOH): λ_{max} 288, 301, 315 nm (log ε = 7.02).

RvD2$_{n\text{-}3\ DPA}$ (1)

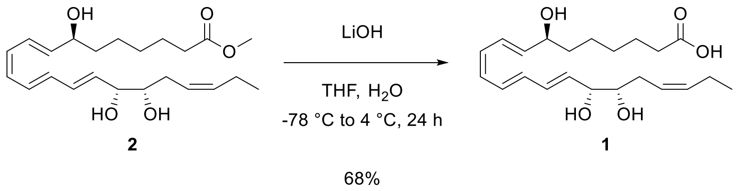

Following a literature procedure,[9] a solution of RvD2$_{n\text{-}3\ DPA}$ methyl ester (**2**, 5.0 μg, 13 nmol) in MeOH was concentrated under a gentle stream of nitrogen gas, dissolved in THF (500 μL), and cooled to −78 °C. To the resulting solution was added 1 M LiOH (50 μL, 50 μmol) and distilled water (one drop, ~20 μL) and the reaction mixture was stirred in a 4 °C cold room for 24 h. The reaction mixture was then concentrated under a gentle stream of nitrogen gas and reconstituted with MeOH (500 μL). The chemical yield of the RvD2$_{n\text{-}3\ DPA}$ free acid **1** was 68% (3.3 μg, 8.9 nmol) post saponification (based on UV−Vis).

UV and MS Experiments for authentic RvD2$_{n\text{-}3\ DPA}$ (1)

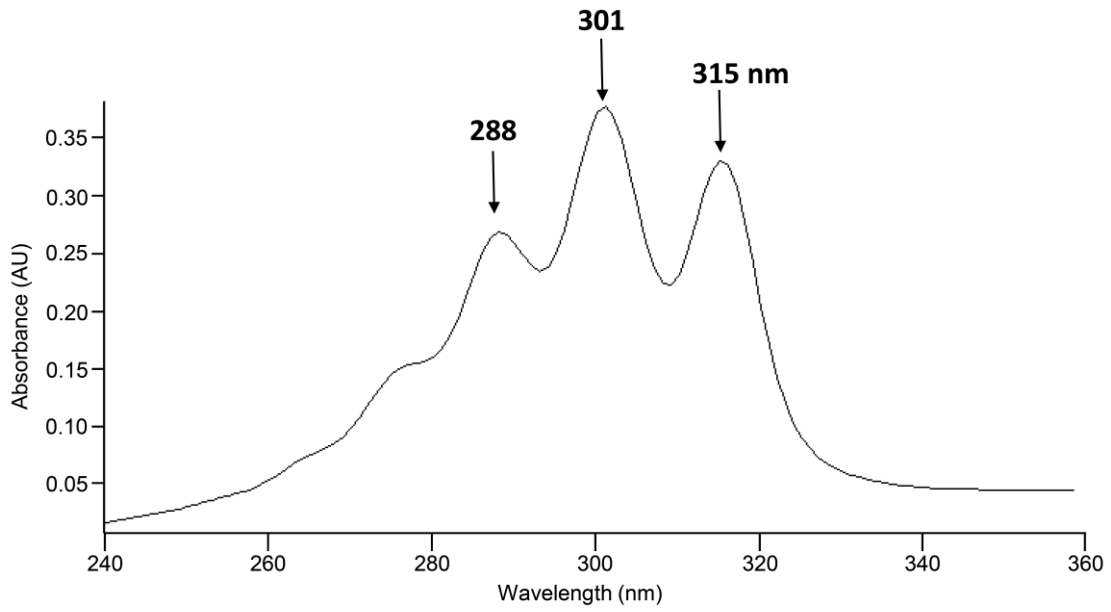

Figure S-1. UV spectrum of endogenous RvD2$_{n\text{-}3\ DPA}$ (**1**).

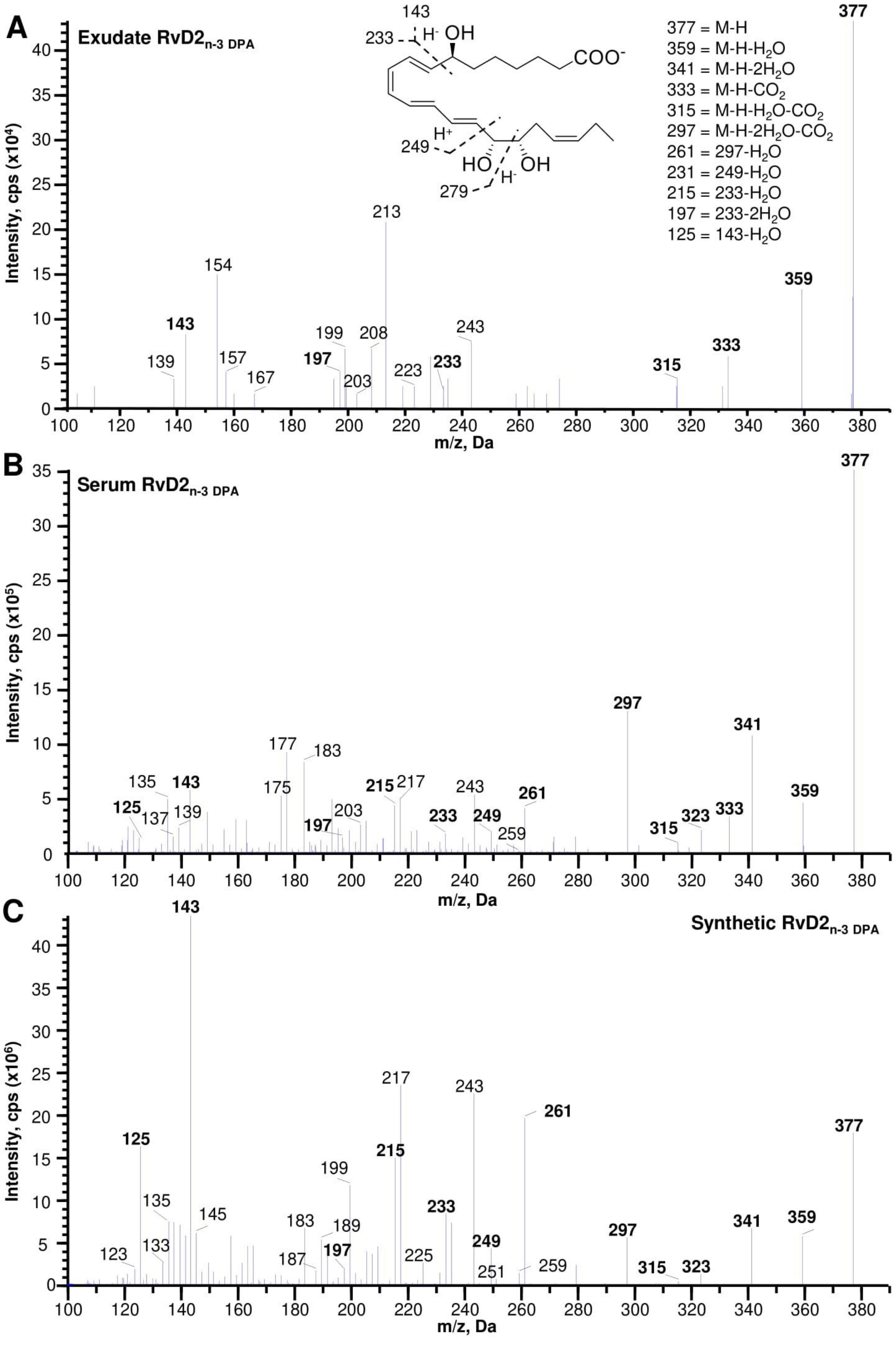

Figure S-2. MS/MS fragmentation spectra from biological RvD2$_{n\text{-}3\ DPA}$ (**1**) and synthetic material of **1**. Lipid mediators were extracted using C18 SPE from (A) inflammatory exudates were collected from mice 2 h after inoculation of *E. coli* (10^5 CFU) via peritoneal lavage (B) human serum was obtained from commercial sources and MS/MS spectra for endogenous RvD2$_{n\text{-}3\ DPA}$ (**1**), together with those of (C) synthetic **1**, were obtained using lipid mediator profiling. Results are from n = 3 mice for A and n = 3 determination for B and C. Ions in bold denoted deduced ions.

Lipid Mediator Profiling

Human serum (0.5 mL; Sigma Aldrich) and mouse exudates were extracted and lipid mediators were identified and quantified as described.[10] In brief, after the addition of ice-cold methanol containing d_5-RvD2, samples were placed at -20 °C (45 min) to allow for protein precipitation. The lipid mediators were extracted using an ExtraHera liquid handling system (Biotage) and solid phase extraction techniques with Isolute C18 500 mg columns (Biotage). Methyl formates were collected, brought to dryness and resuspended in phase (methanol/water, 1:1, vol/vol) for injection on a Shimadzu LC-20AD HPLC and a Shimadzu SIL-20AC autoinjector, paired with a QTrap 6500+ (Sciex). The QTRAP was operated in negative ion mode using a multiple reaction monitoring method coupled with an information dependent acquisition to an Enhanced Product Ion experiment. An Agilent Poroshell 120 EC-C18 column (100 mm × 4.6 mm × 2.7 μm) was kept at 50 °C and mediators eluted using a mobile phase consisting of methanol/water/acetic acid of 20:80:0.01 (vol/vol/vol) that was ramped to 50:50:0.01 (vol/vol/vol) over 0.5 min and then to 80:20:0.01 (vol/vol/vol) from 2 min to 11 min, maintained till 14.5 min and then rapidly ramped to 98:2:0.01 (vol/vol/vol) for the next 0.1 min. This was subsequently maintained at 98:2:0.01 (vol/vol/vol) for 5.4 min, and the flow rate was maintained at 0.5 ml/min and the presence of $RvD2_{n\text{-}3\ DPA}$ (**1**) was monitored using multiple reaction monitoring with QM set to an *m/z* 377 and Q3 to *m/z* of 143.

MS/MS spectral matching was conducted in accordance with published criteria with the matching of retention time and at least 6 diagnostic ions to that of reference standard, with a minimum of one backbone fragment being identified.[10] Furthermore, we also required a minimum of 70% match between the biological and reference standard spectra as determined by the SCIEX OS library match algorithm.

Evaluations of bone marrow-derived macrophage differentiation

The experiments strictly adhered to UK Home Office regulations (Guidance on the Operation of Animals, Scientific Procedures Act, 1986) and Laboratory Animal Science Association (LASA) Guidelines (Guiding Principles on Good Practice for Animal Welfare and Ethical Review Bodies, 3rd Edition, 2015) and according to protocols detailed in a UK Home Office approved protocol (P998AB295).

***E. coli* peritonitis**

Male C57/Bl6 mice were inoculated with 10^5 CFU of *E. coli* via i.p. injection. After 2 h the peritoneum was lavages with 4 mL of sterile PBS, 2 volumes of ice-cold methanol containing 500 pg of deuterium labelled d_5-RvD2 were added to the lavages and lipid mediators were extracted as detailed below.

Bone marrow-derived macrophage differentiation

Generation of bone marrow-derived macrophages (BMDM). Male C57BL6/J mice were culled using CO_2 and bone marrow (BM) from femurs and tibias flushed using Ca^{2+}- and Mg^{2+} free DPBS (Sigma, D8537). BM cells were left to adhere to 10 cm^2 petri dishes for 45 min at 37 °C and 5% CO_2 and non-adherent cells were washed away. Cells were subsequently cultured using RPMI (Sigma, R8758) complemented with 10% fetal bovine serum (Gibco, 10270106), 1% Penicillin/Streptomycin (Gibco, 15140122) and 20 ng/mL of GM-CSF (Peprotech, 315-03-100UG), for 7 days, with media refreshed every 3 days. BMDM were detached by incubating for 15 min at 37 °C and 5% CO_2 with 5 mM EDTA in Ca^{2+}- and Mg^{2+} free DPBS and plated in black 96-well plate (Greiner, 655090) at a number of 50,000 per well and nuclei were stained using 1 μg/mL Hoechst H3342 (Thermofisher, P37165) for 15 min at 37 °C. pHrodo Green-labelled *S. aureus* (5 μg/well)

or pHrodo Red- labelled zymosan A bioparticles (2.5 µg/well) (Invitrogen, P35367 and P35364) were opsonized by incubating in 20% (v/v) human serum (Sigma, H4522) for 30 min at 37 °C. BMDM were incubated with $RvD2_{n\text{-}3\ DPA}$ (**1**) (10 nM to 0.01 nM) for 15 min or vehicle (0.01% EtOH in RPMI) at 37 °C and 5% CO_2, before adding 5 µg/well of opsonized pHrodo Green-labelled *S. aureus* bioparticles. The accumulation of pHrodo Green or pHrodo Red fluorescence, indicative of bioparticle engulfment in phago-lysosomes, was then recorded using a Celldiscoverer 7 high content imaging system (Zeiss) during the subsequent 120 min.

^{1}H- and $^{13}C\{^{1}H\}$ NMR Spectra

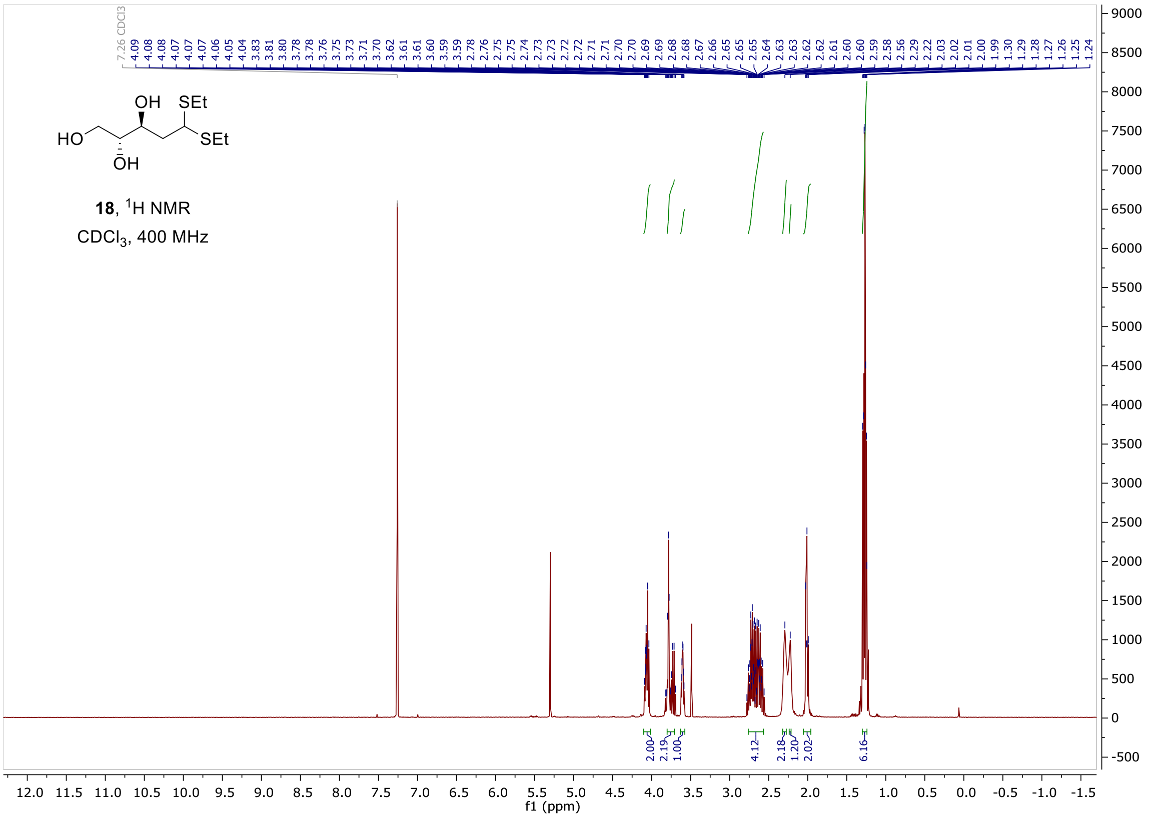

Figure S-3. ^{1}H NMR spectrum of compound **18**.

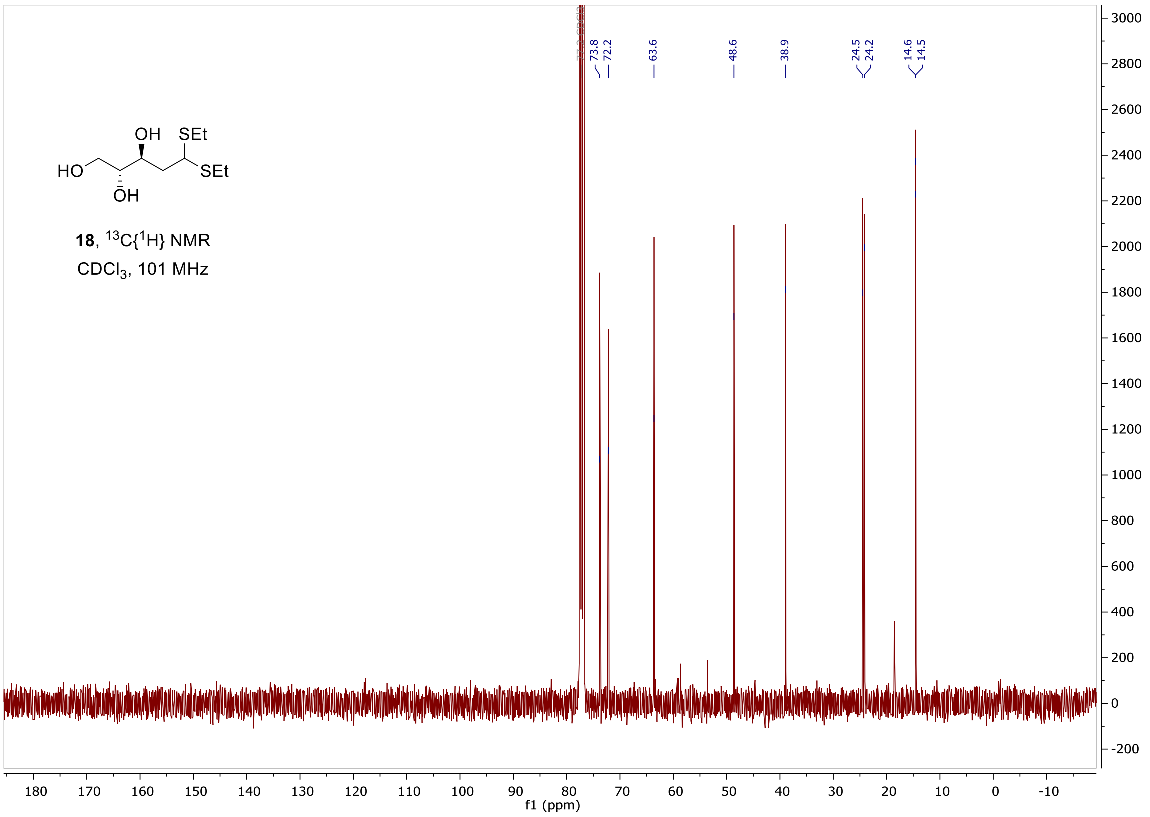

Figure S-4. ^{13}C{^{1}H} NMR spectrum of compound **18**.

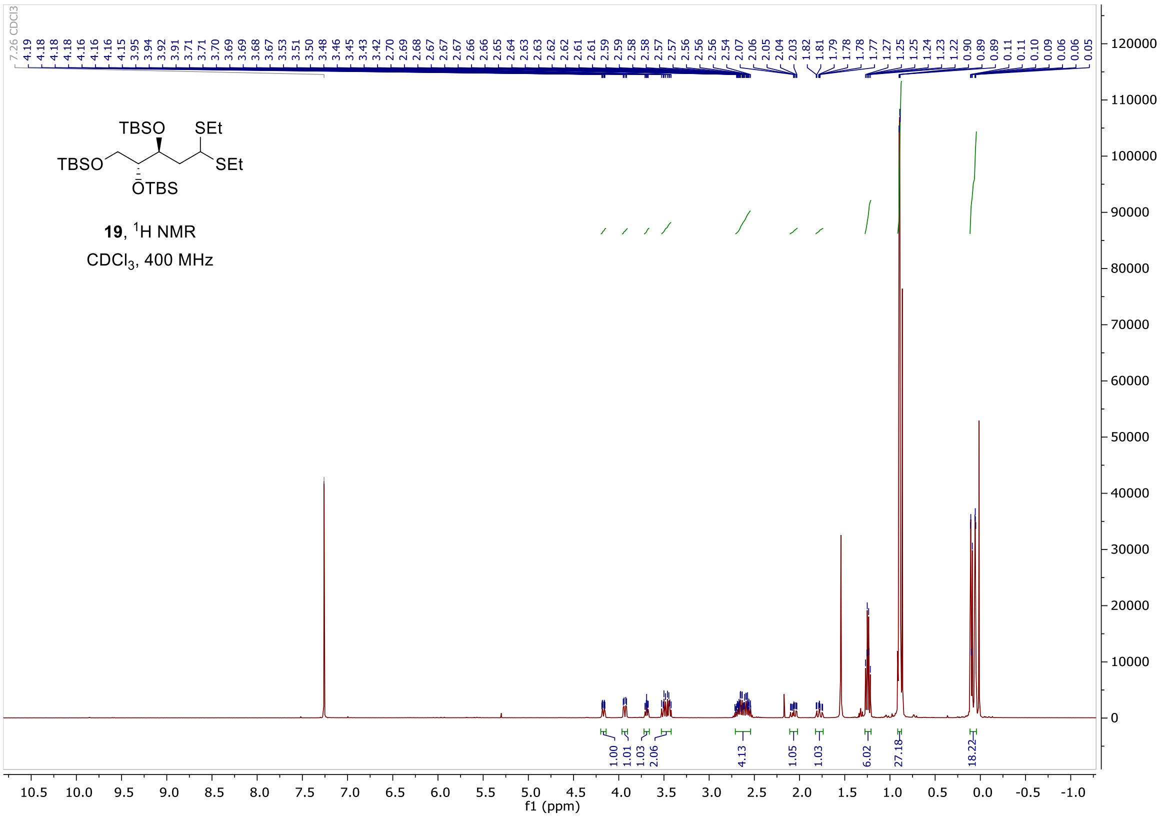

Figure S-5. ^{1}H NMR spectrum of compound **19**.

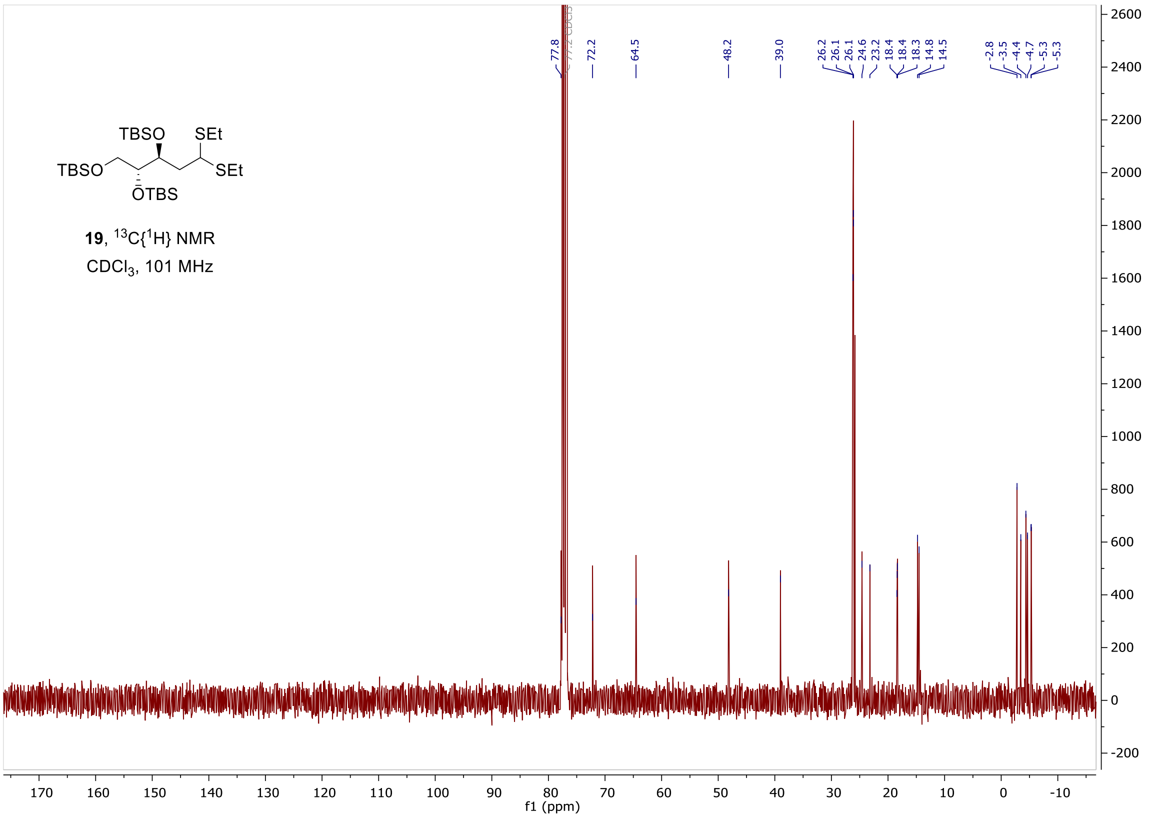

Figure S-6. ^{13}C{^{1}H} NMR spectrum of compound **19**.

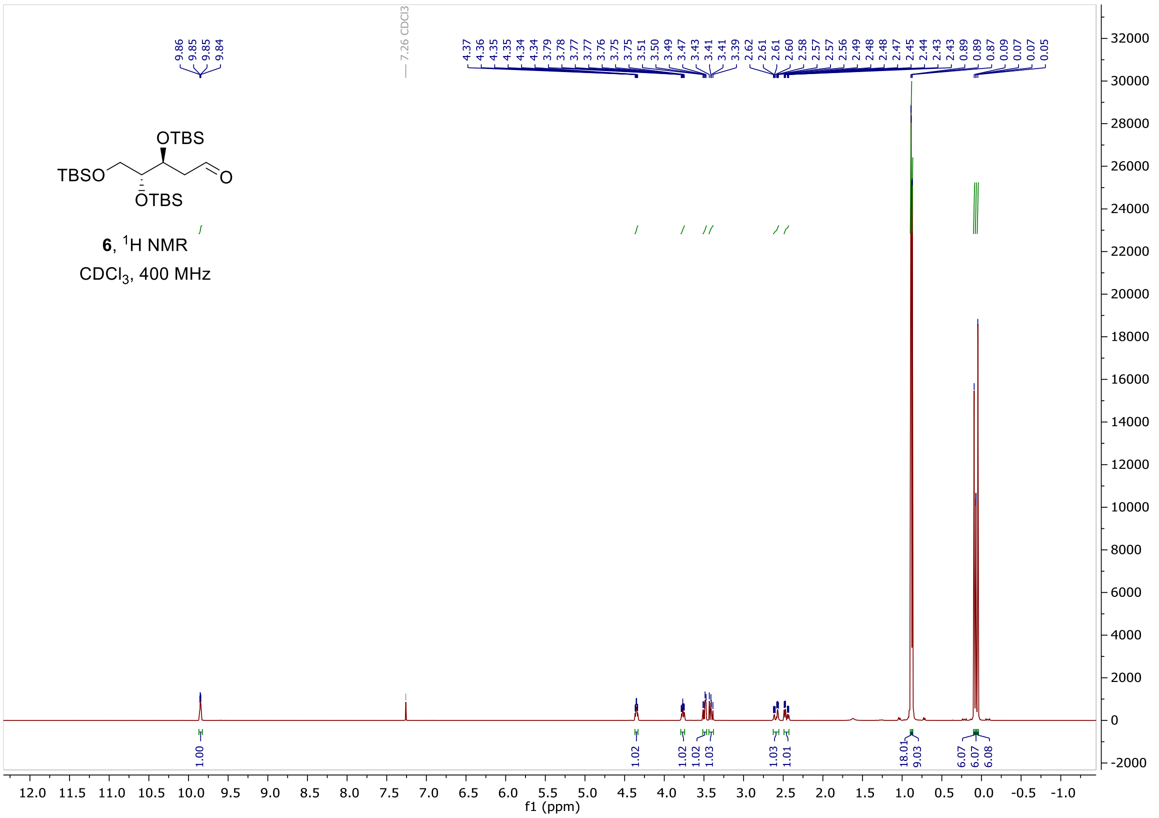

Figure S-7. ^{1}H NMR spectrum of compound **6**.

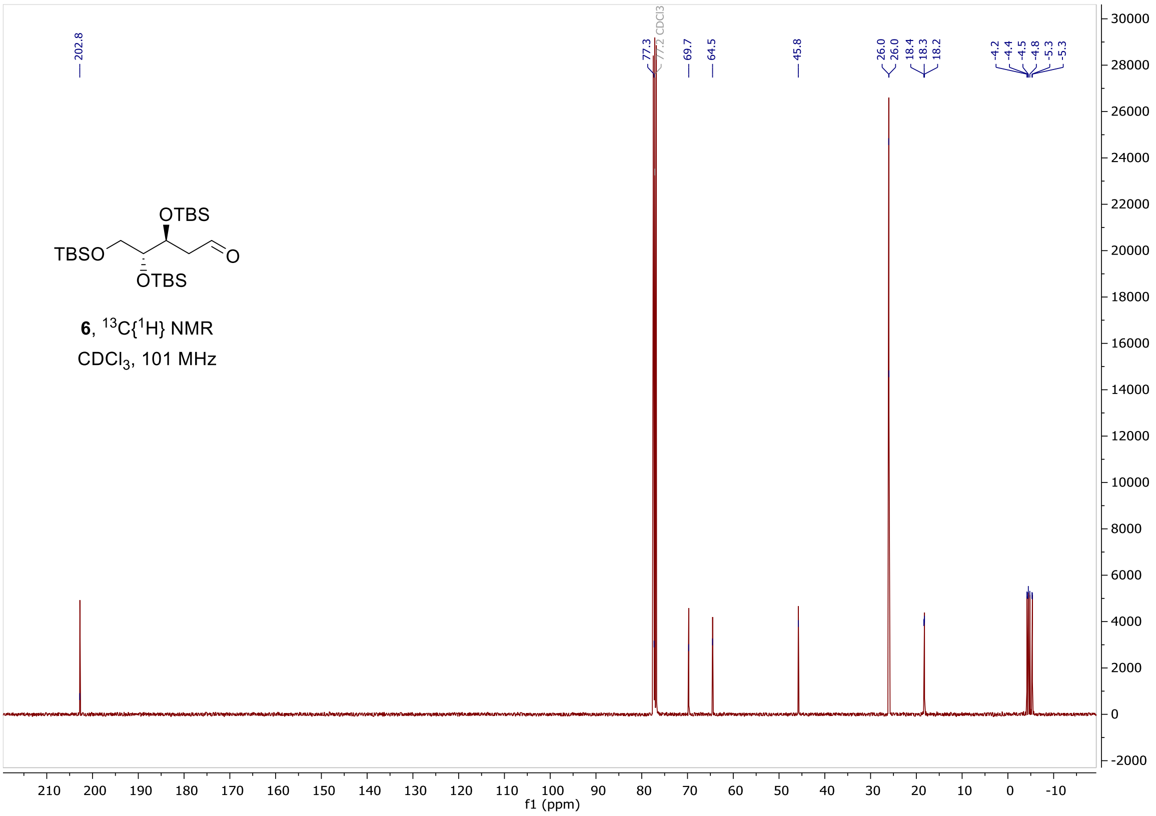

Figure S-8. $^{13}C\{^{1}H\}$ NMR spectrum of compound **6**.

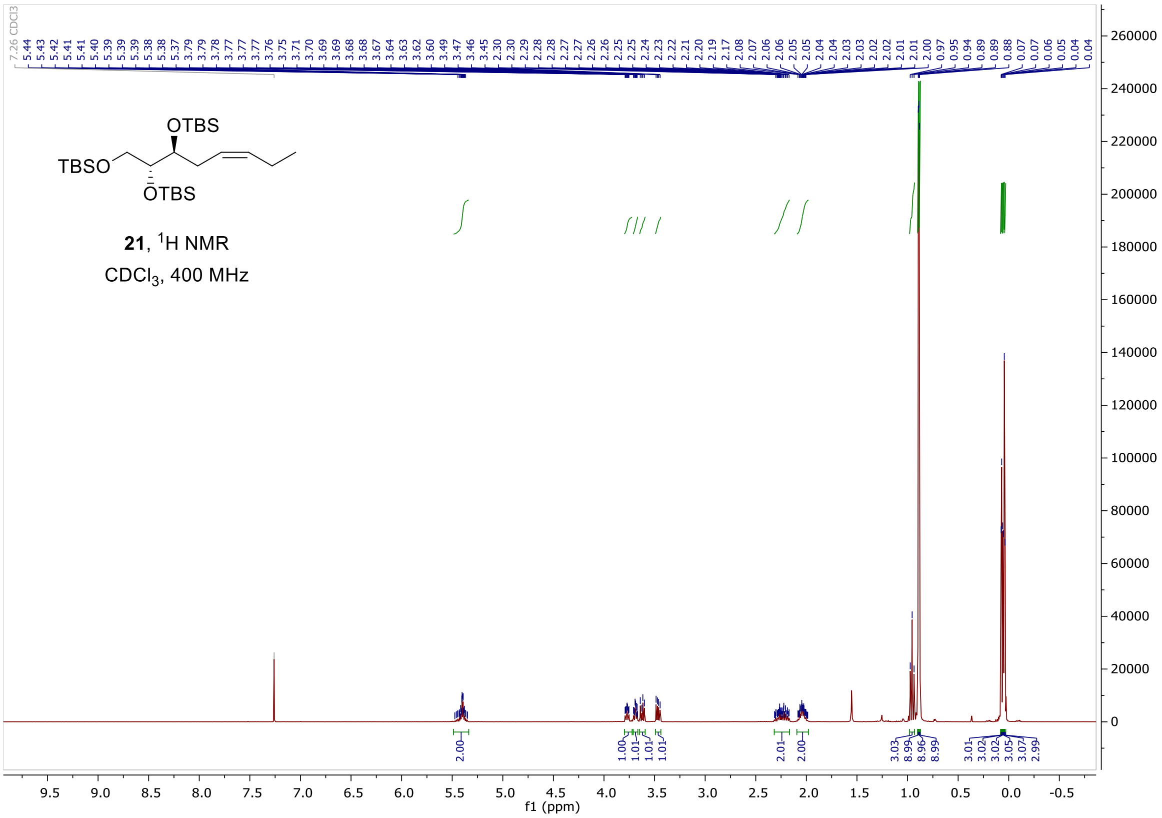

Figure S-9. ^{1}H NMR spectrum of compound **21**.

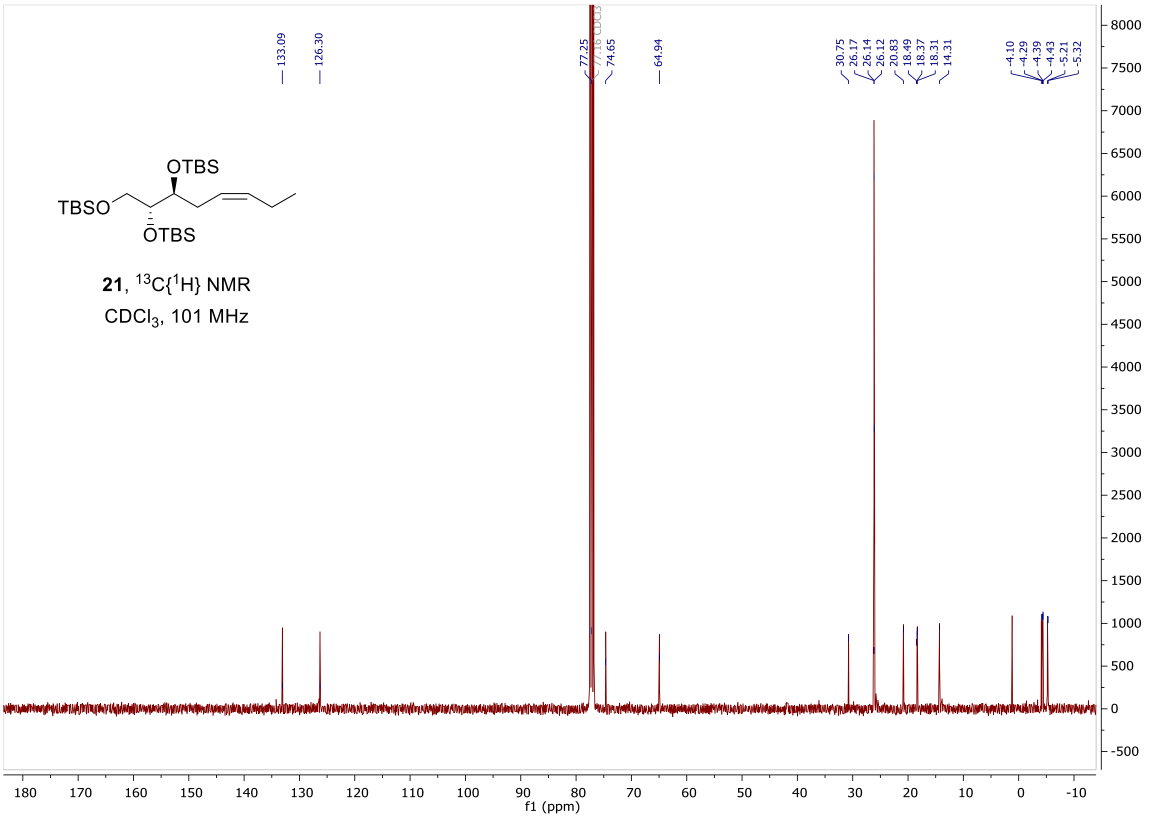

Figure S-10. $^{13}C\{^{1}H\}$ NMR spectrum of compound **21**.

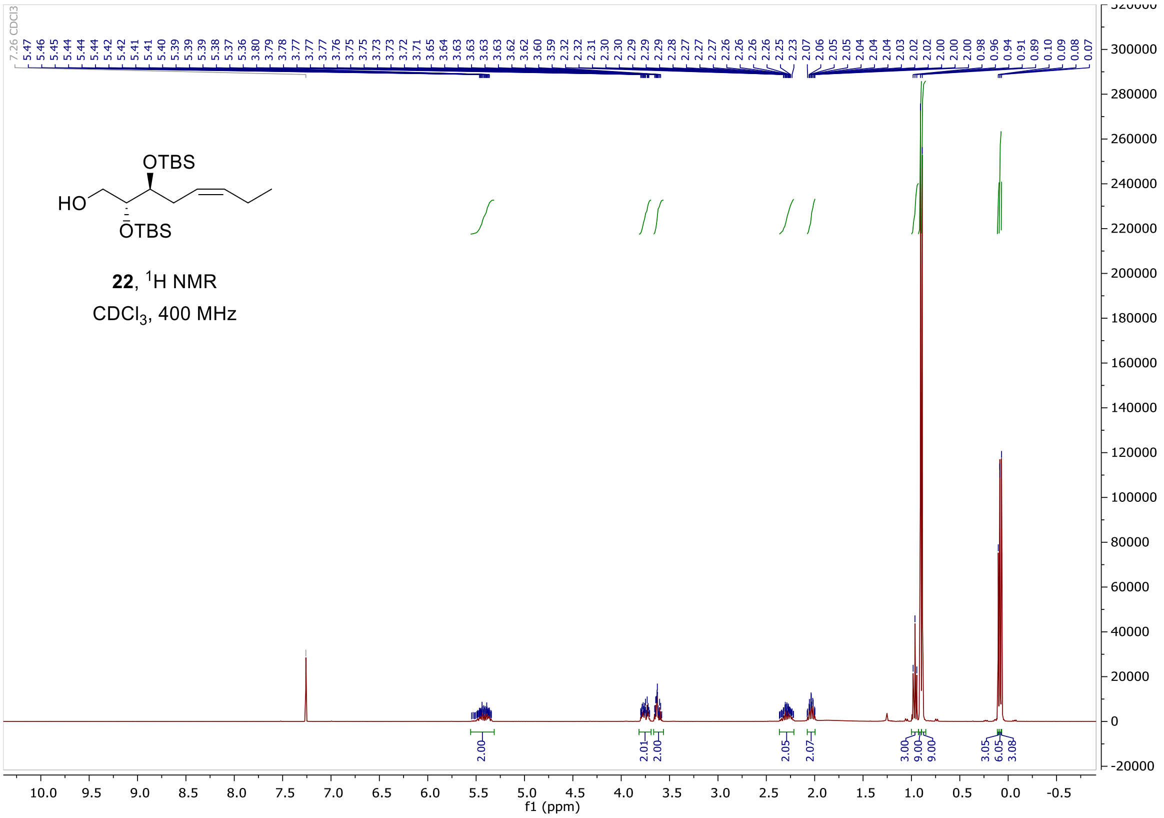

Figure S-11. ^{1}H NMR spectrum of compound **22**.

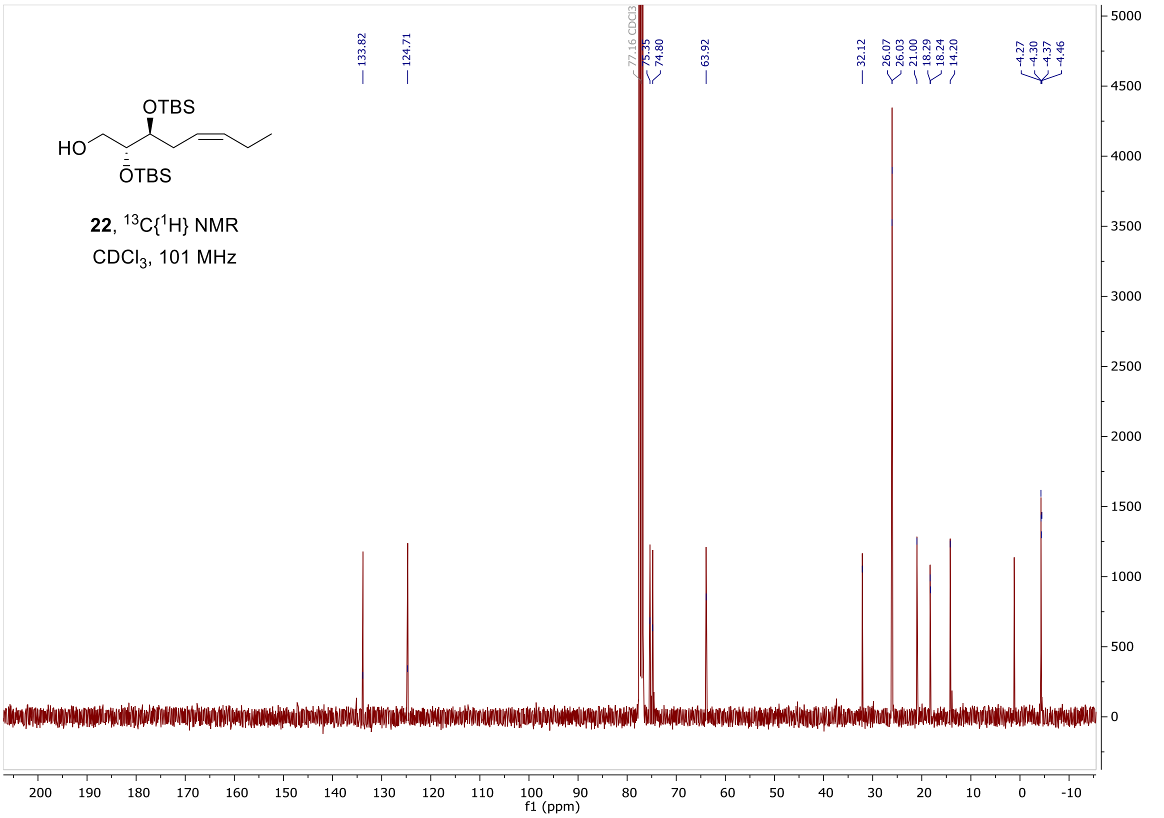

Figure S-12. ^{13}C{^{1}H} NMR spectrum of compound **22**.

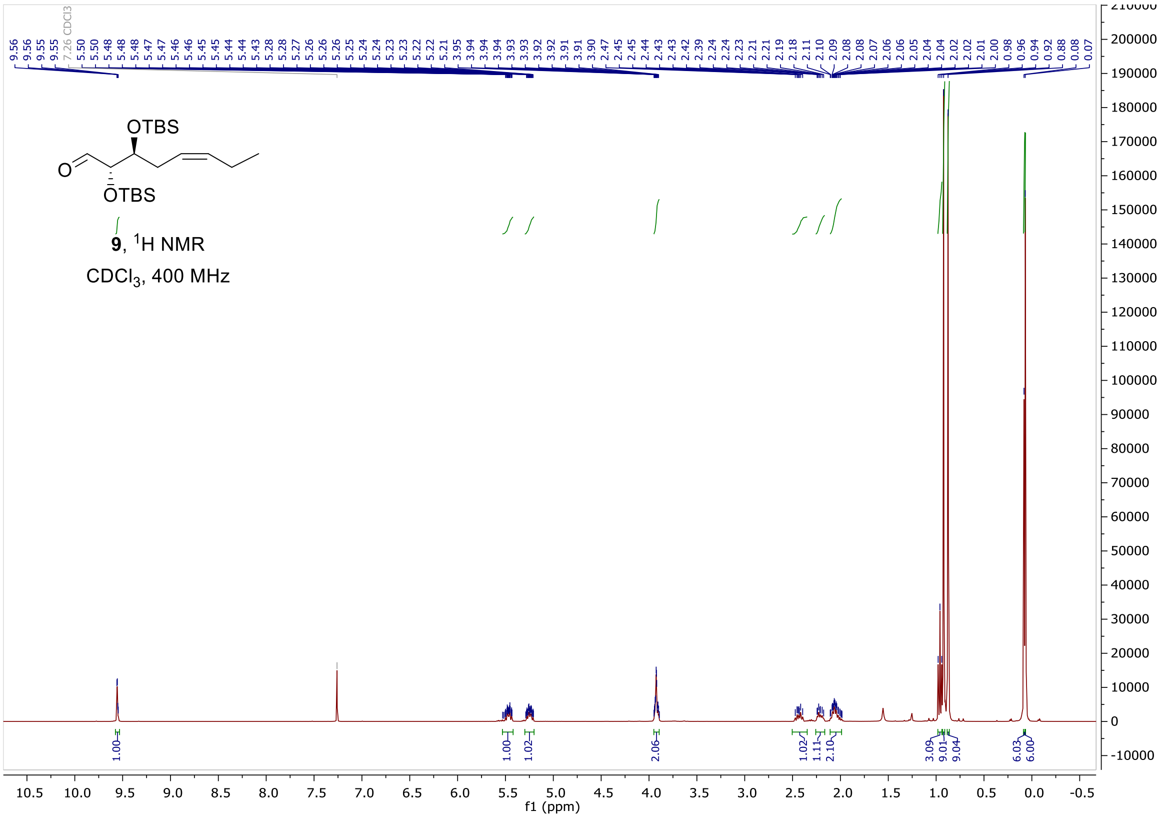

Figure S-13. ^{1}H NMR spectrum of compound **9**.

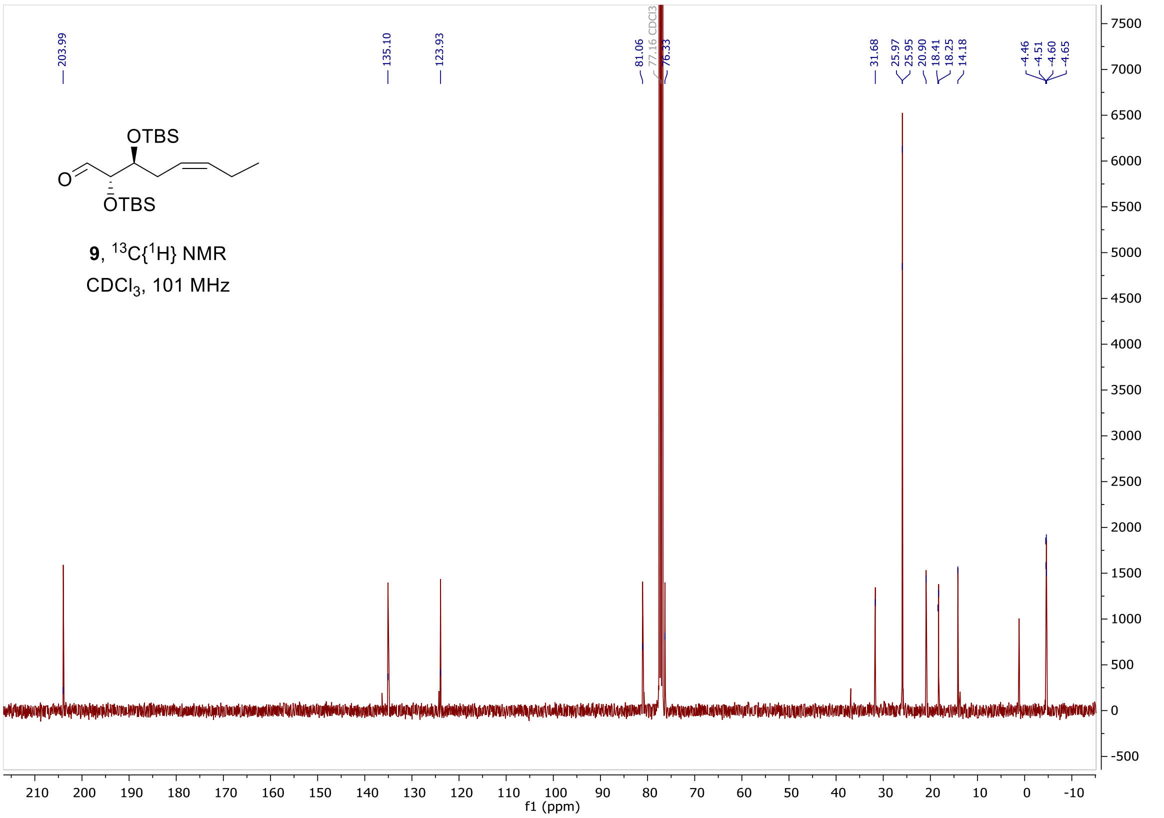

Figure S-14. ^{13}C{^{1}H} NMR spectrum of compound **9**.

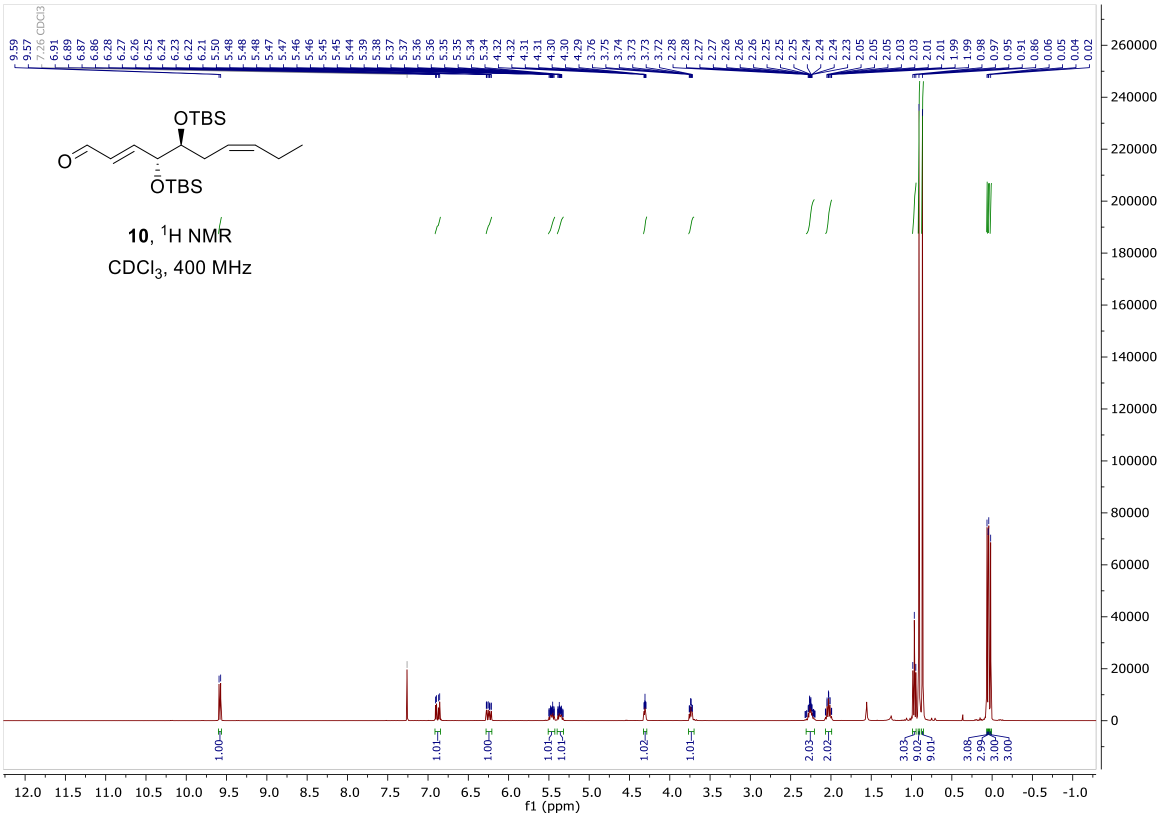

Figure S-15. ^{1}H NMR spectrum of compound **10**.

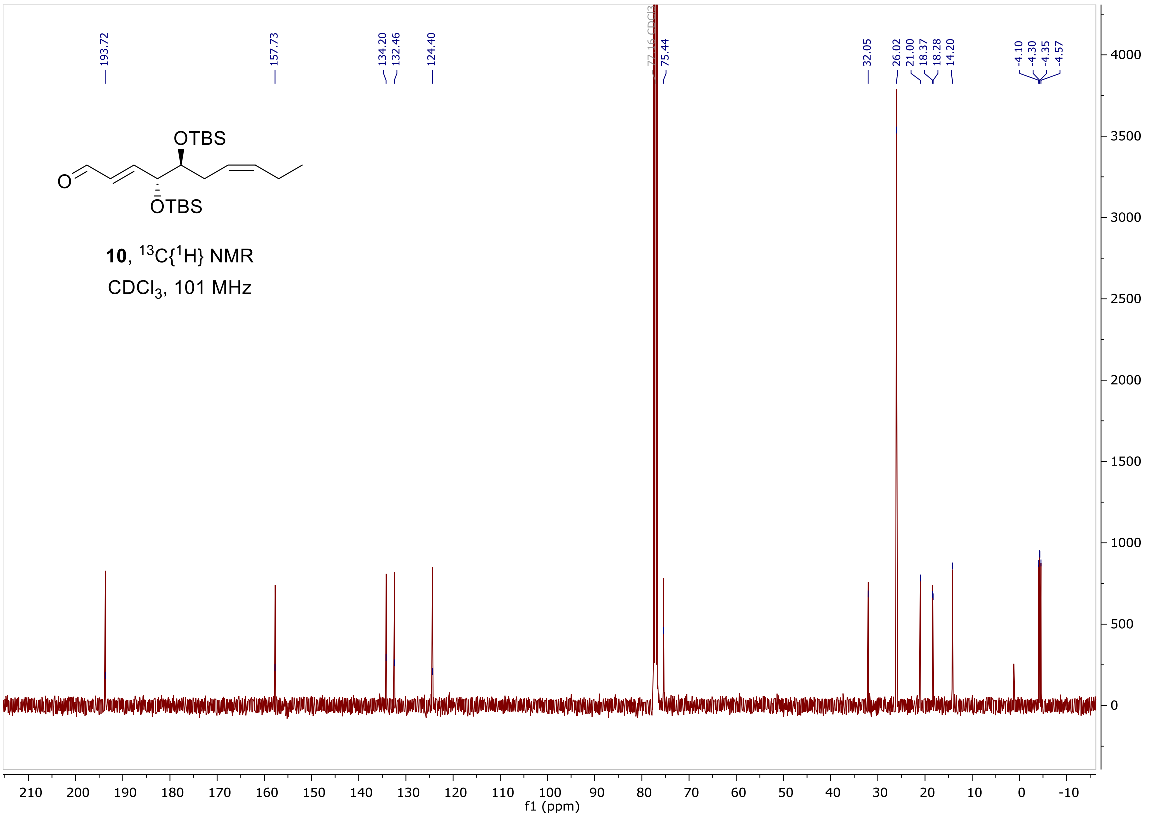

Figure S-16. ^{13}C{^{1}H} NMR spectrum of compound **10**.

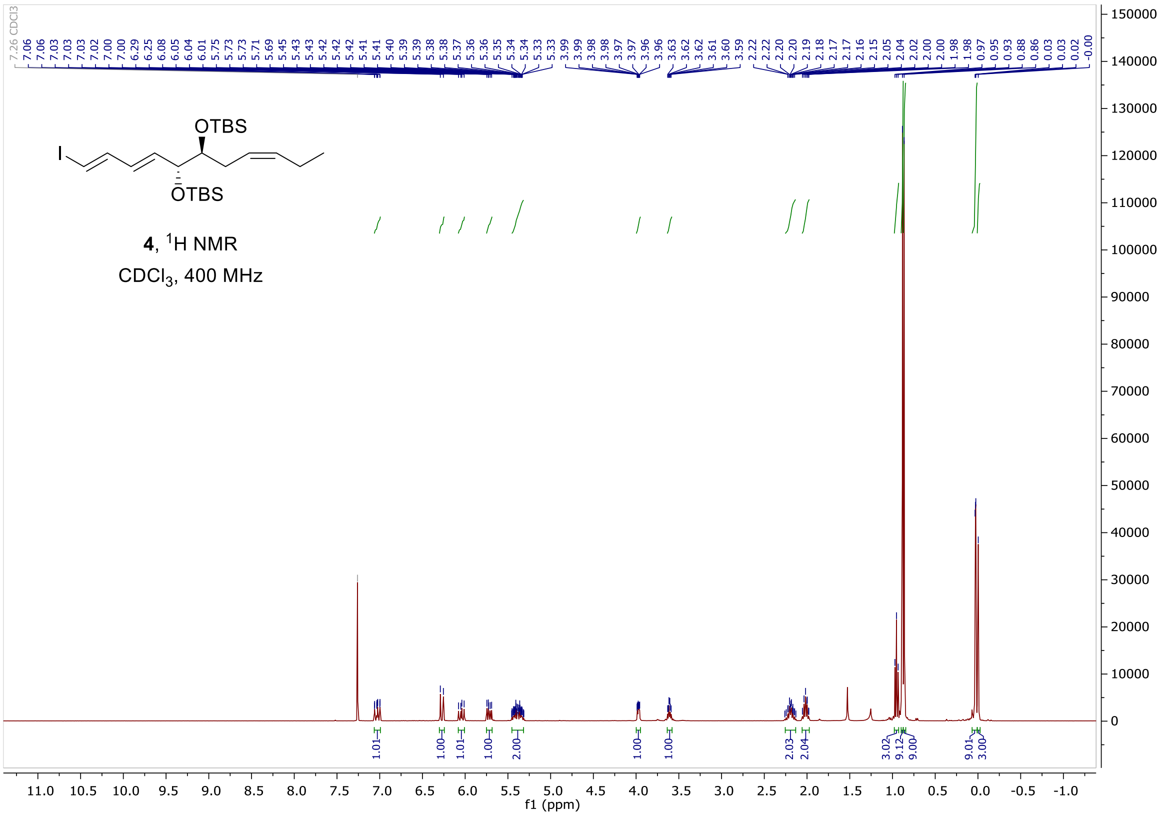

Figure S-17. ^{1}H NMR spectrum of compound **4**.

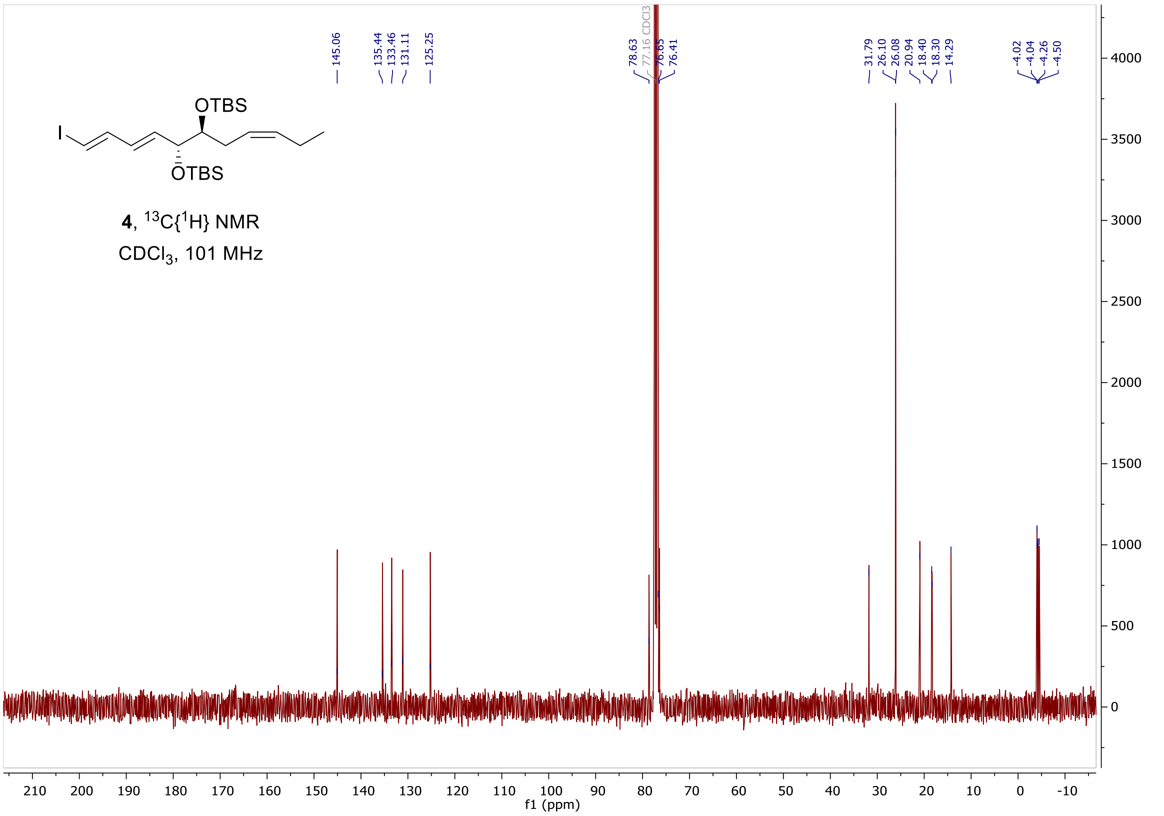

Figure S-18. ^{13}C{^{1}H} NMR spectrum of compound **4**.

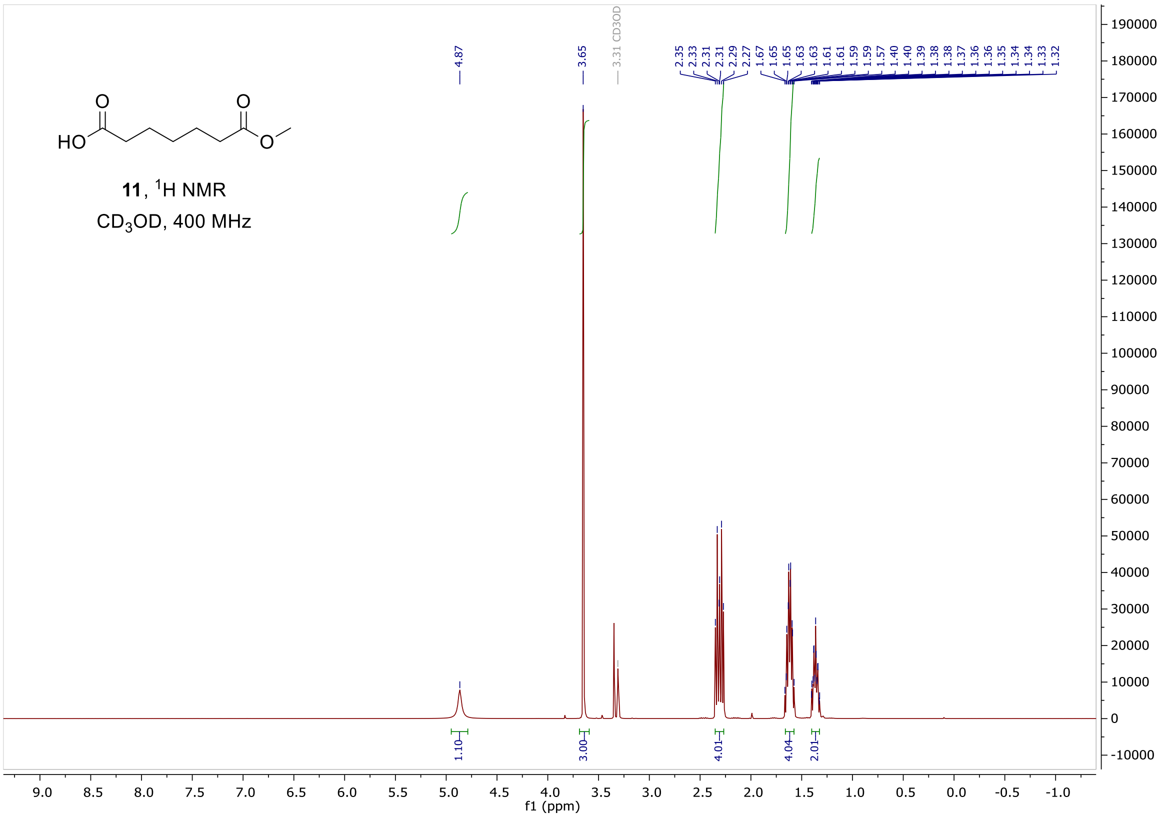

Figure S-19. ^{1}H NMR spectrum of compound **11**.

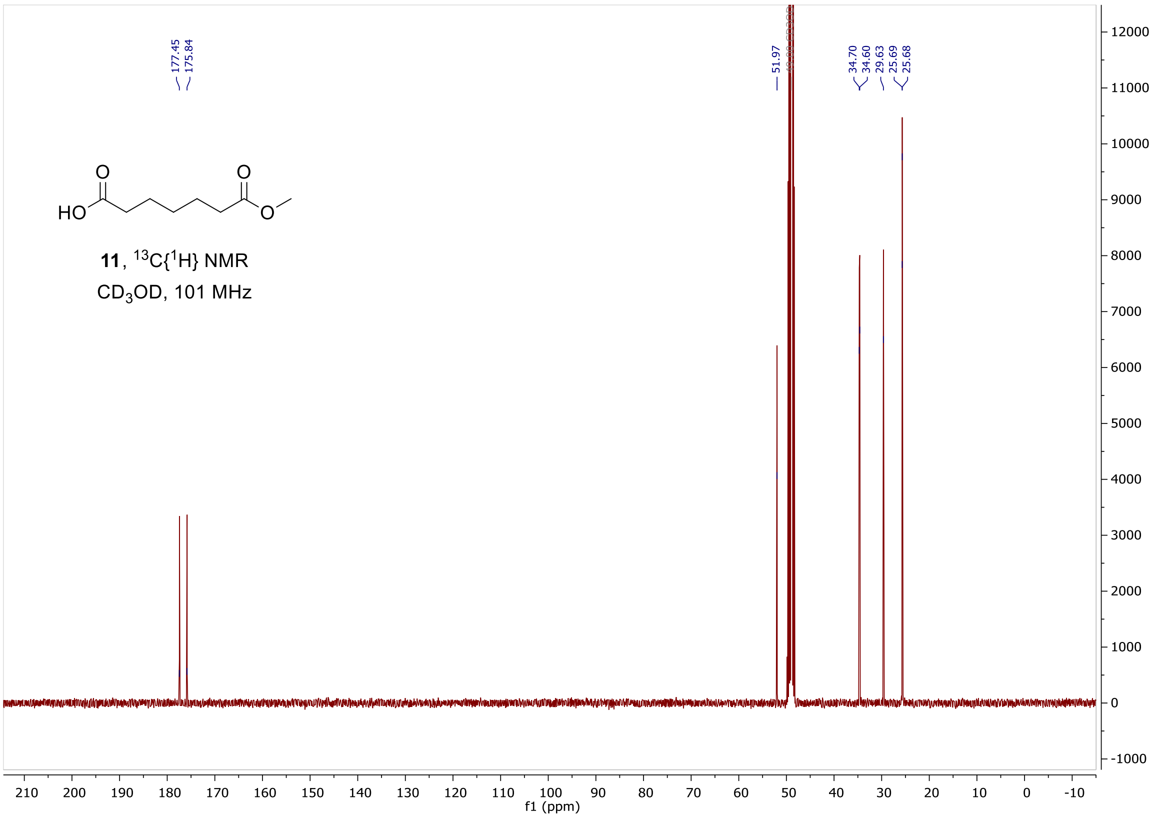

Figure S-20. $^{13}C\{^{1}H\}$ NMR spectrum of compound **11**.

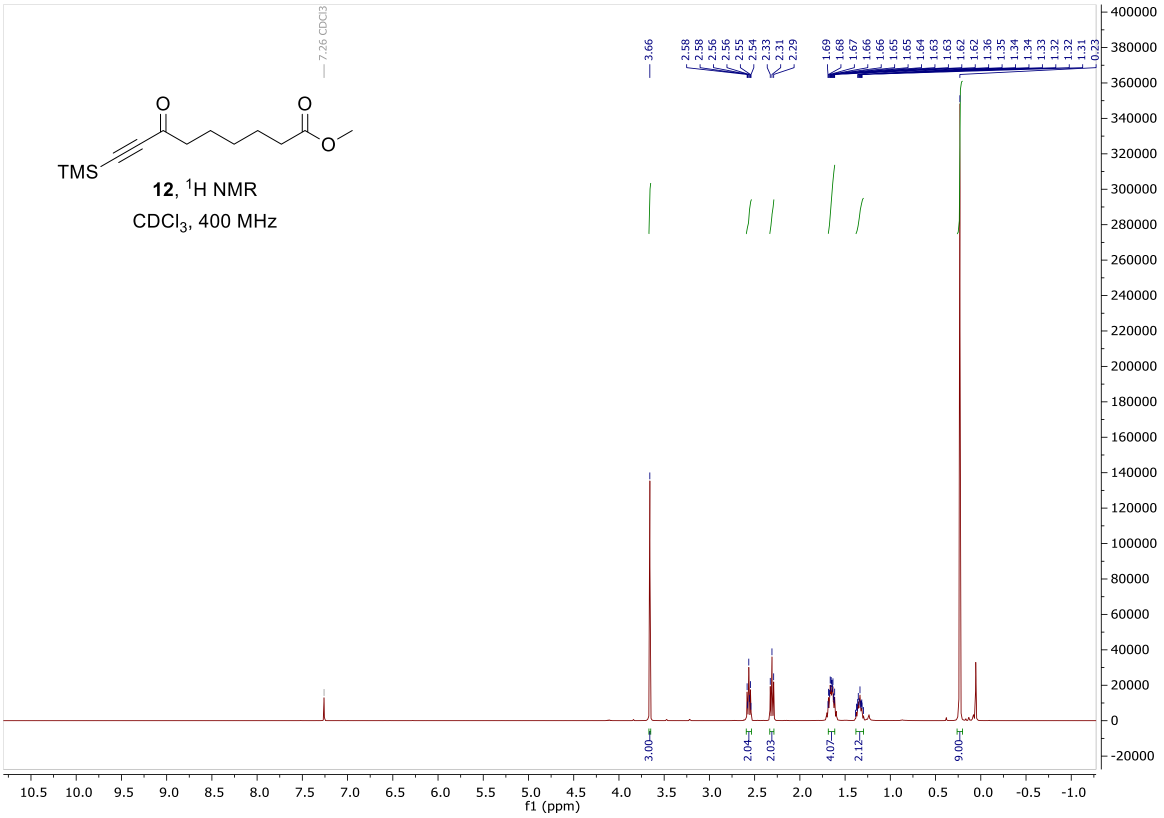

Figure S-21. ^{1}H NMR spectrum of compound **12**.

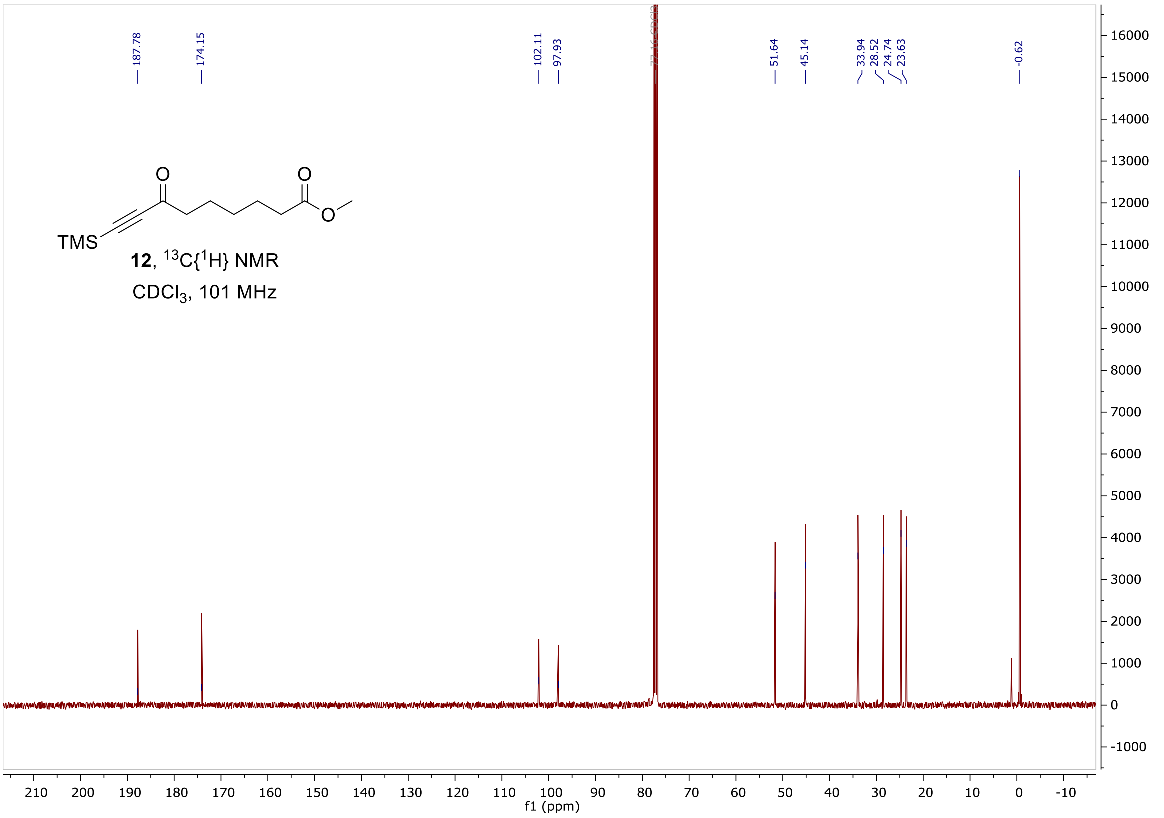

Figure S-22. ^{13}C{^{1}H} NMR spectrum of compound **12**.

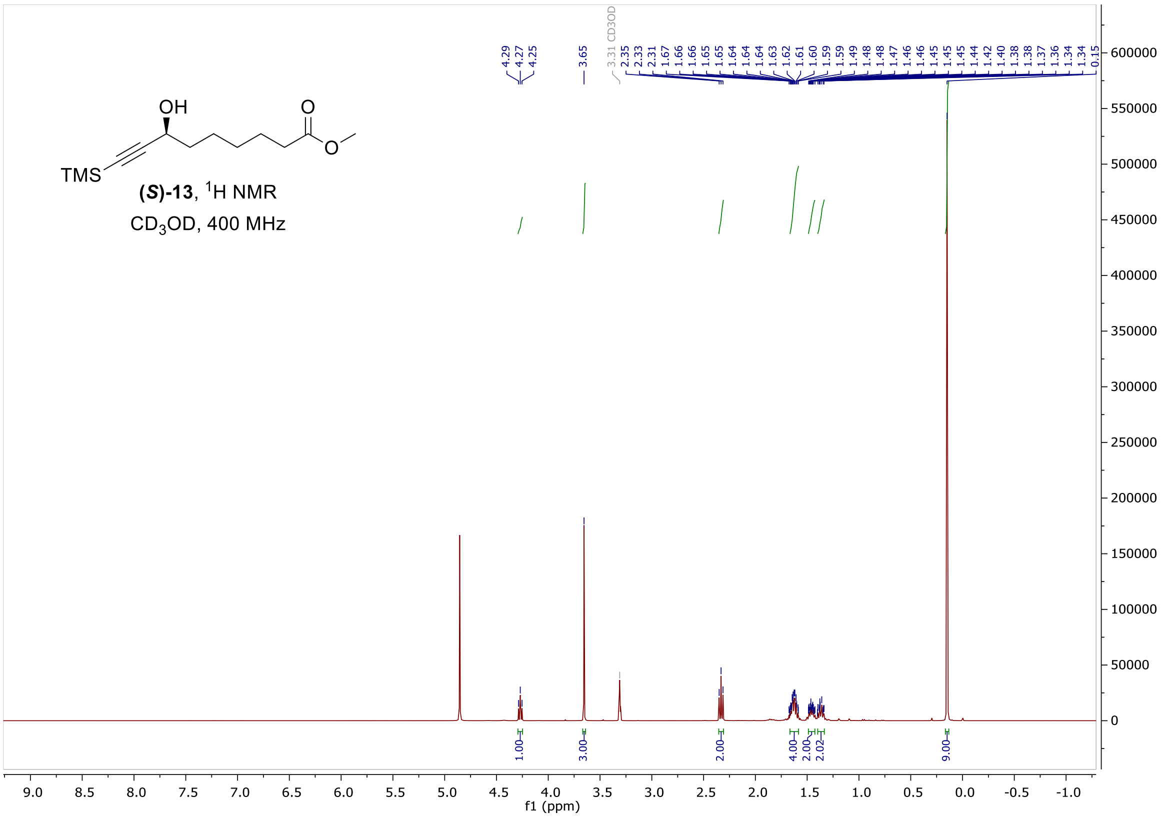

Figure S-23. ^{1}H NMR spectrum of compound (*S*)-**13**.

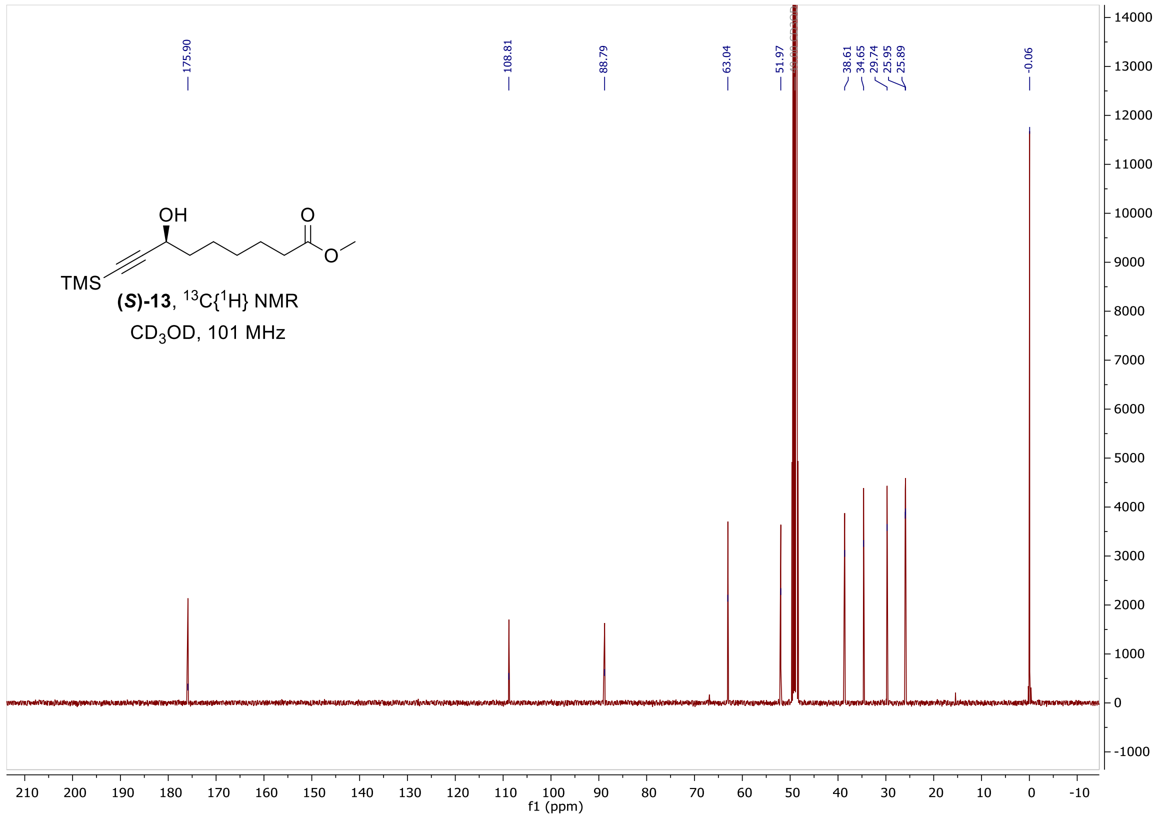

Figure S-24. ^{13}C{^{1}H} NMR spectrum of compound (*S*)-**13**.

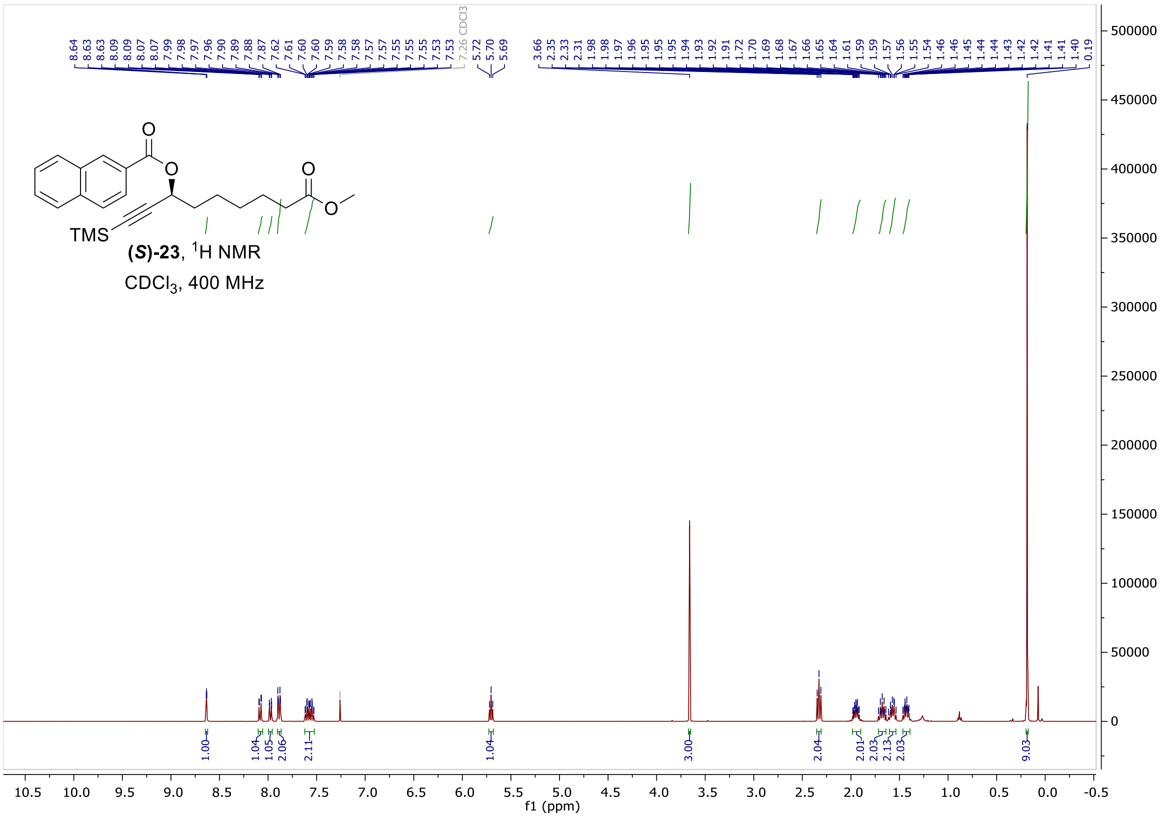

Figure S-25. ^{1}H NMR spectrum of compound (*S*)-**23**.

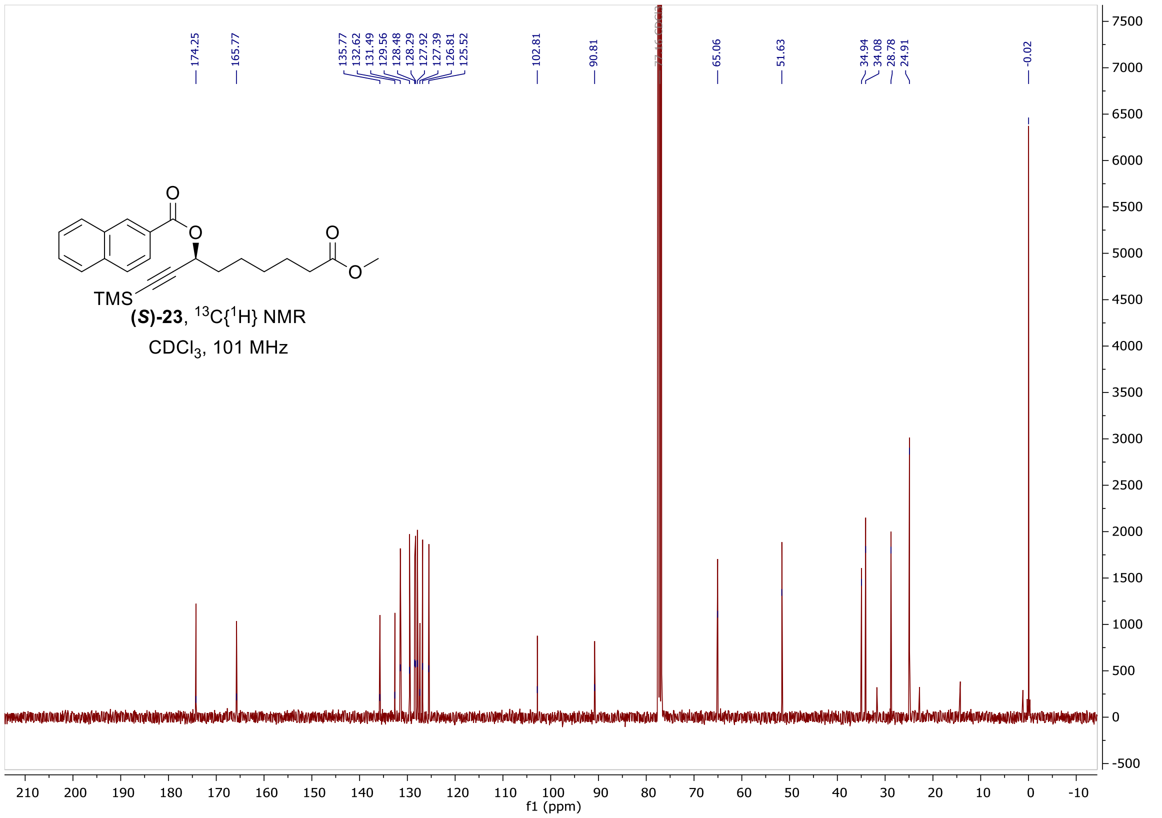

Figure S-26. ^{13}C{^{1}H} NMR spectrum of compound (*S*)-**23**.

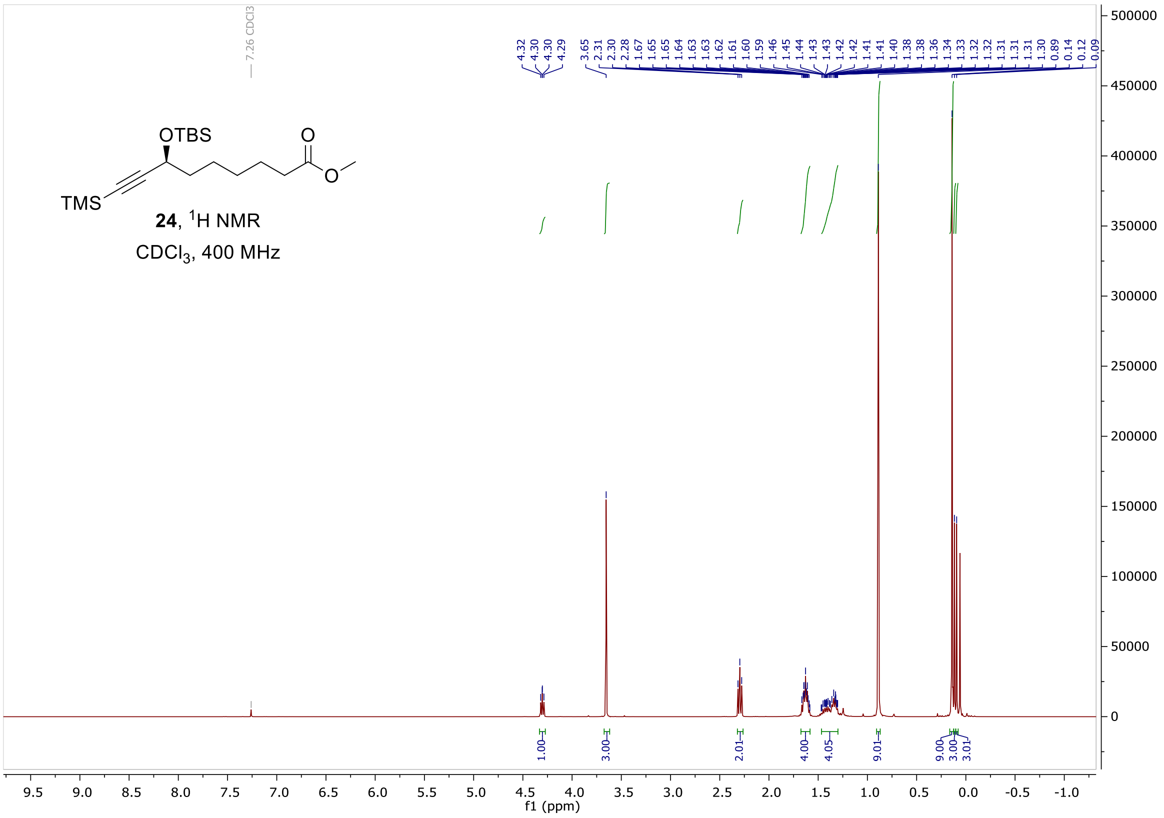

Figure S-27. ^{1}H NMR spectrum of compound **24**.

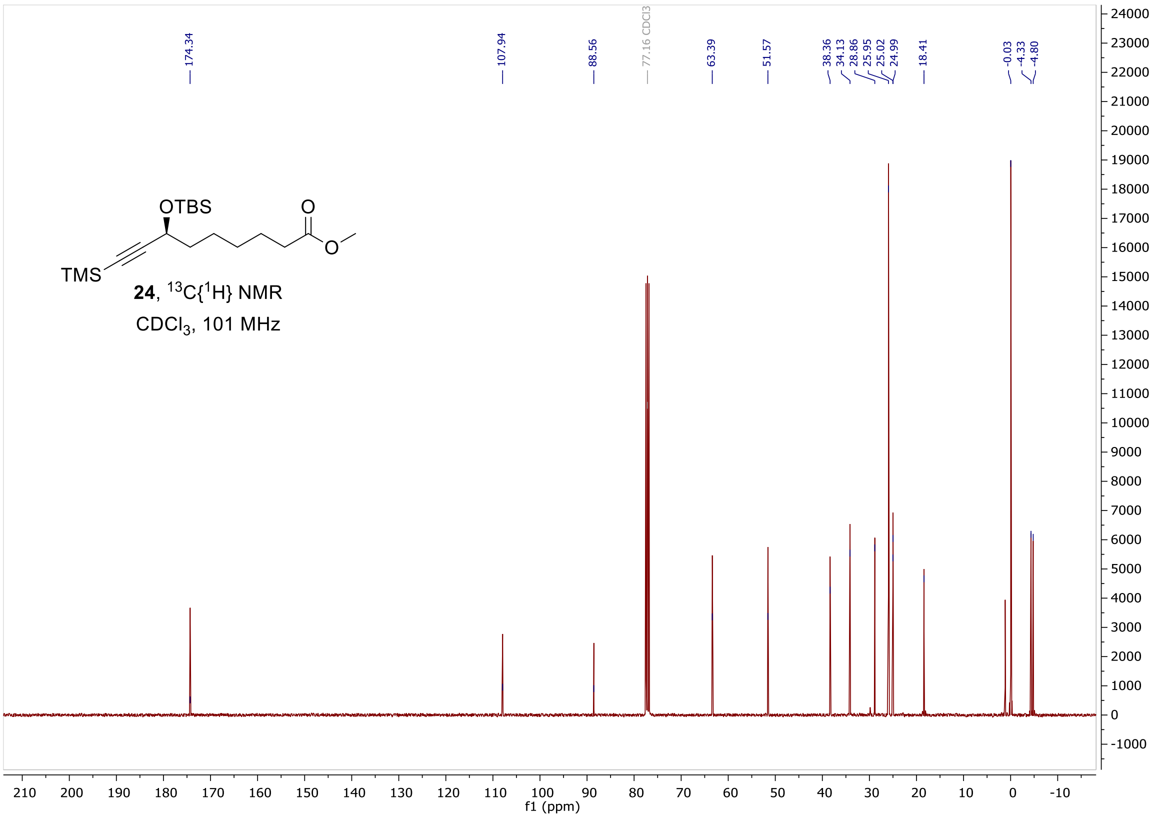

Figure S-28. ^{13}C{^{1}H} NMR spectrum of compound **24**.

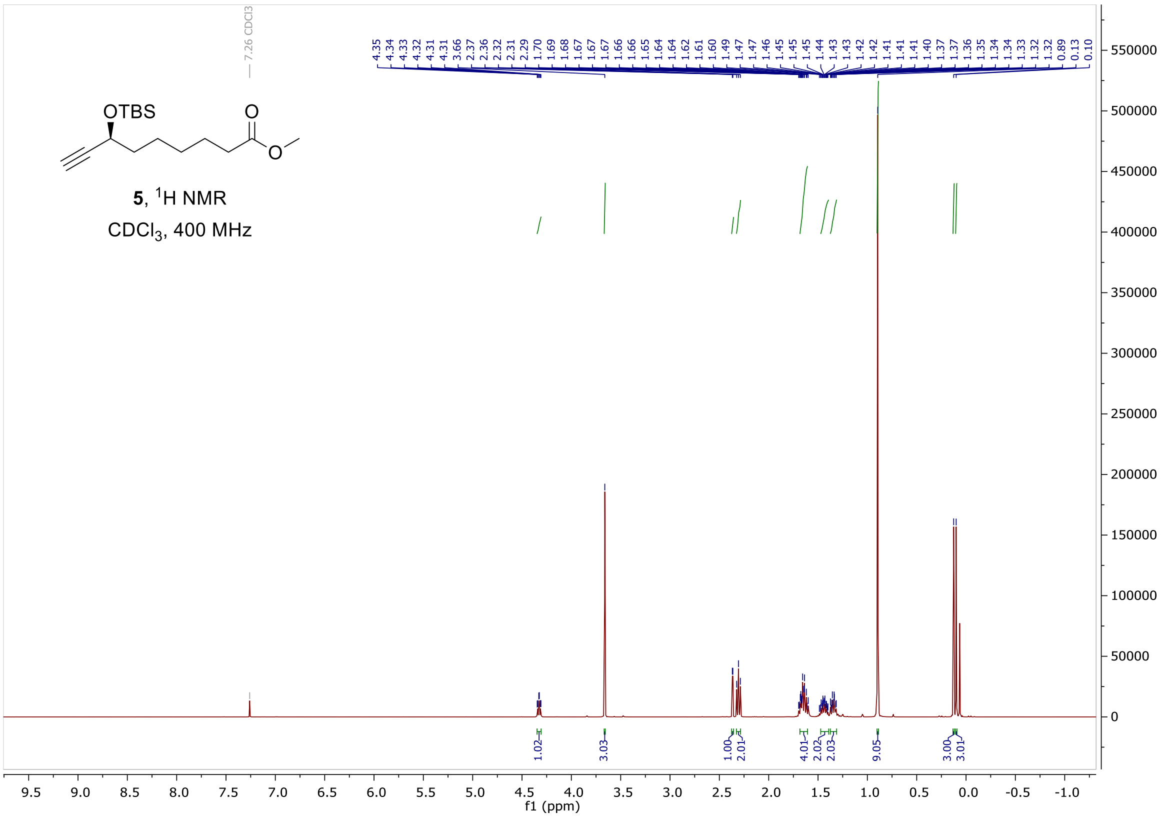

Figure S-29. ^{1}H NMR spectrum of compound **5**.

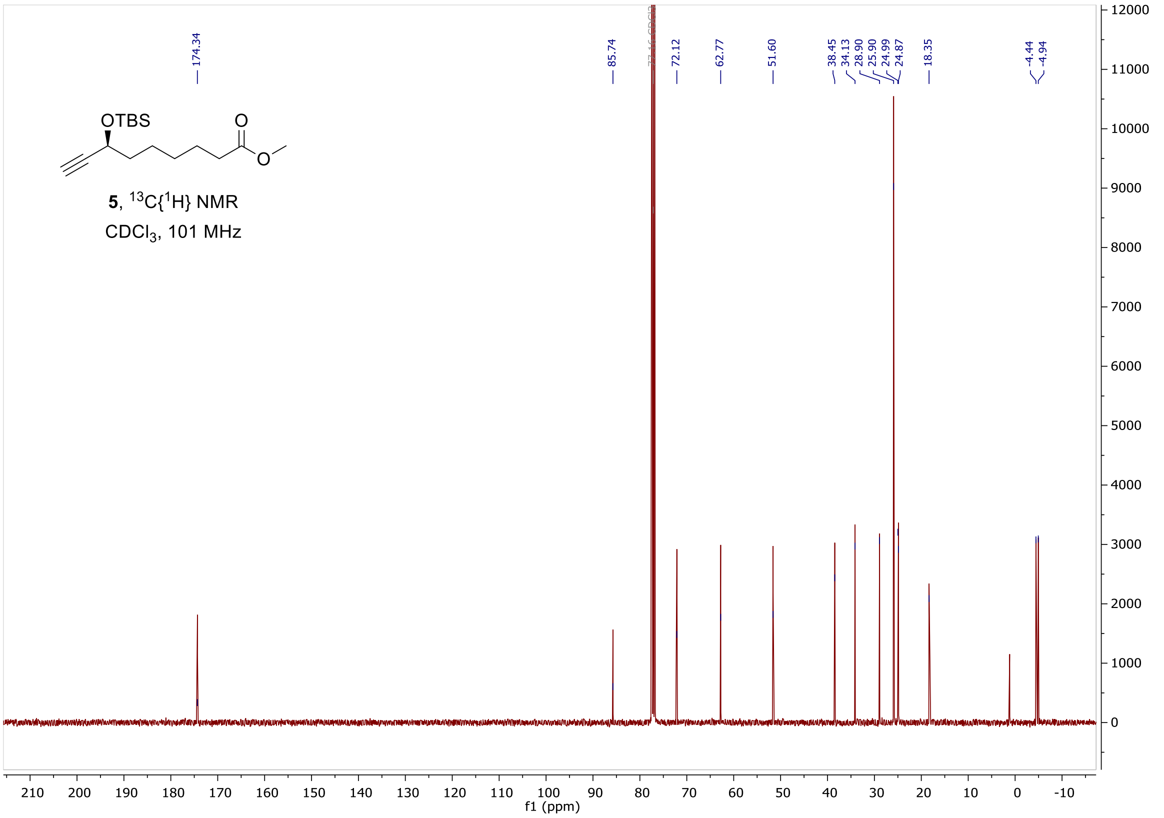

Figure S-30. ^{13}C{^{1}H} NMR spectrum of compound **5**.

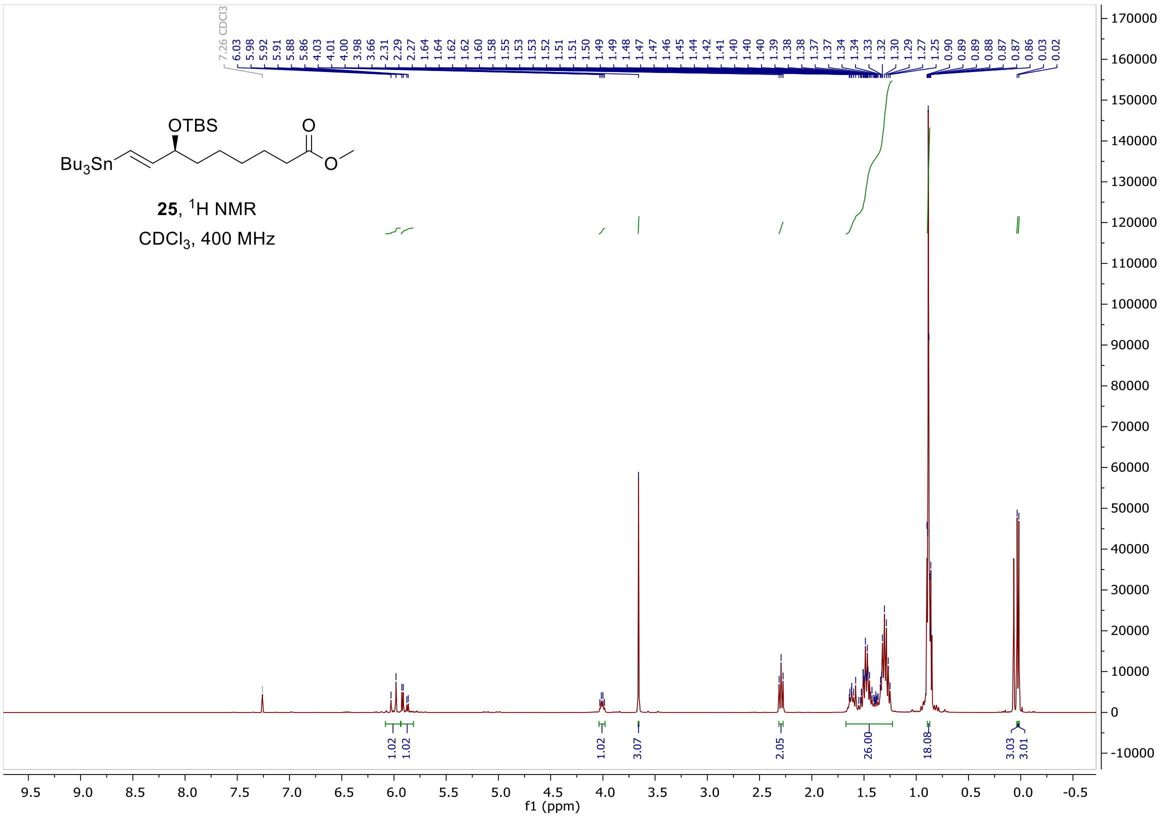

Figure S-31. ^{1}H NMR spectrum of compound **25**.

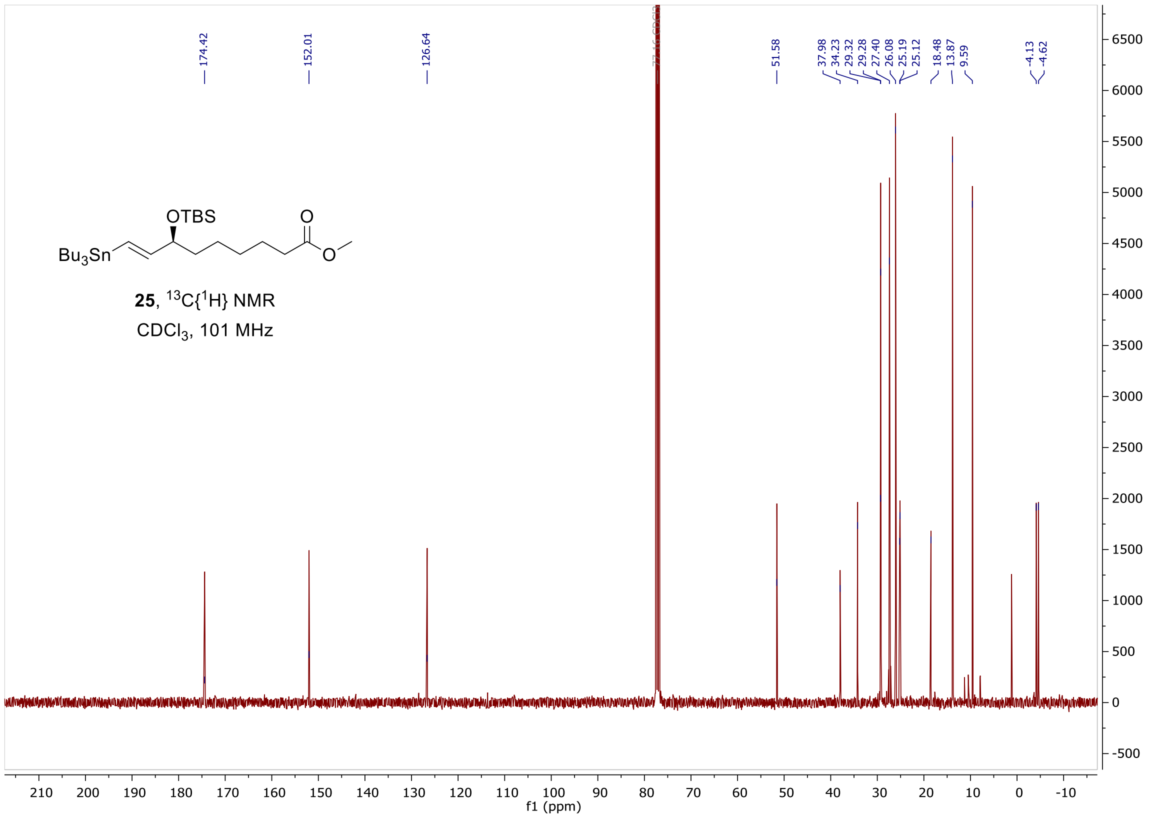

Figure S-32. ^{13}C{^{1}H} NMR spectrum of compound **25**.

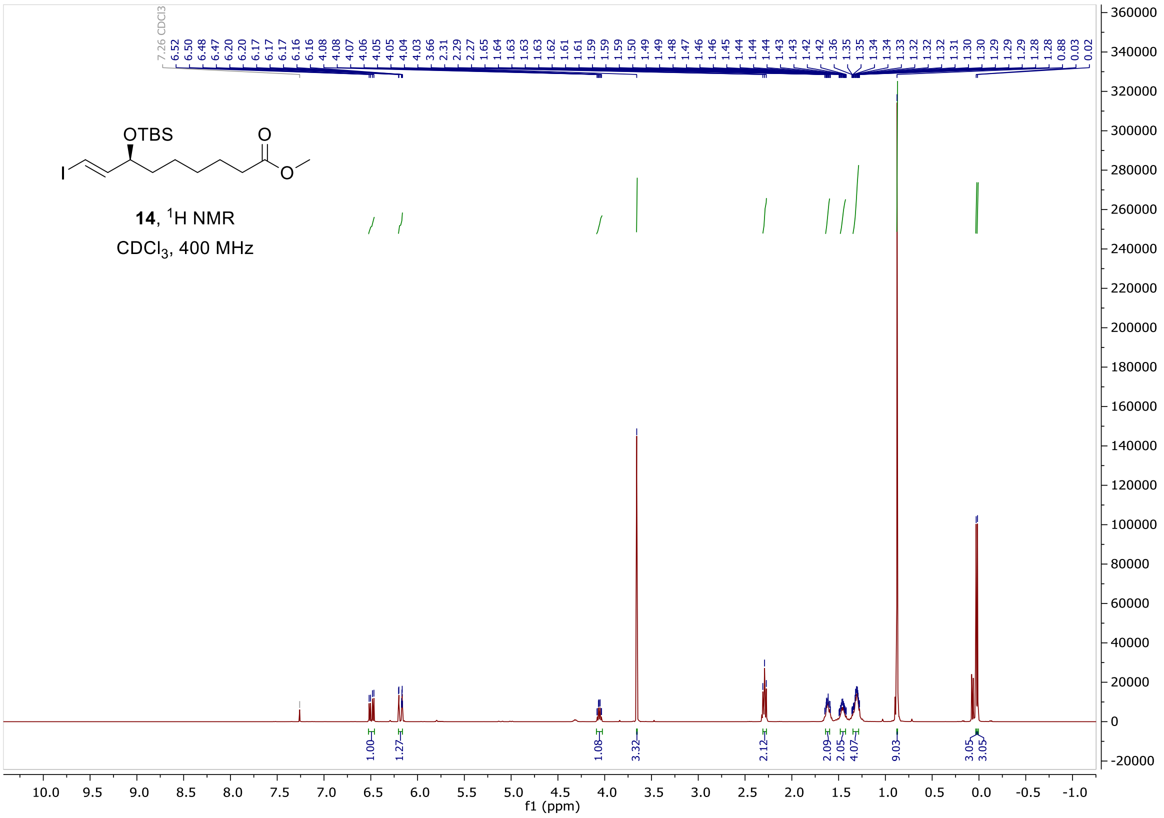

Figure S-33. ^{1}H NMR spectrum of compound **14**.

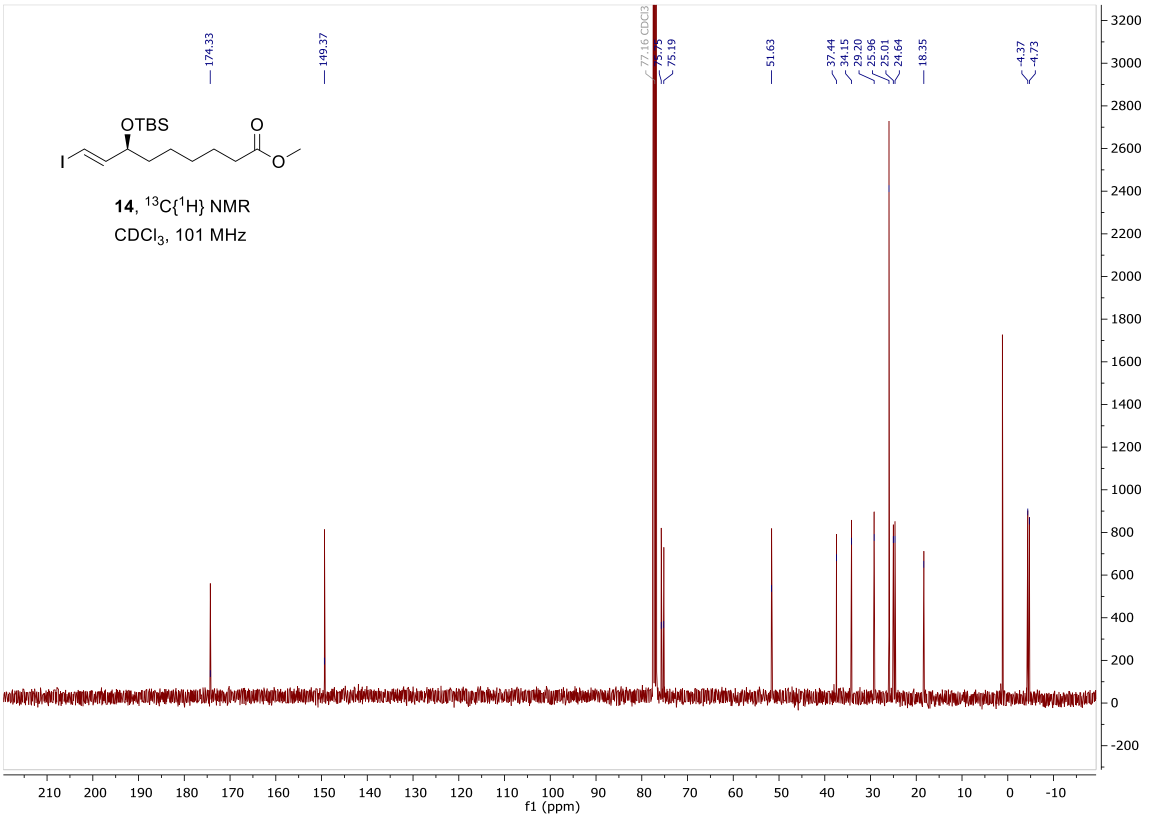

Figure S-34. ^{13}C{^{1}H} NMR spectrum of compound **14**.

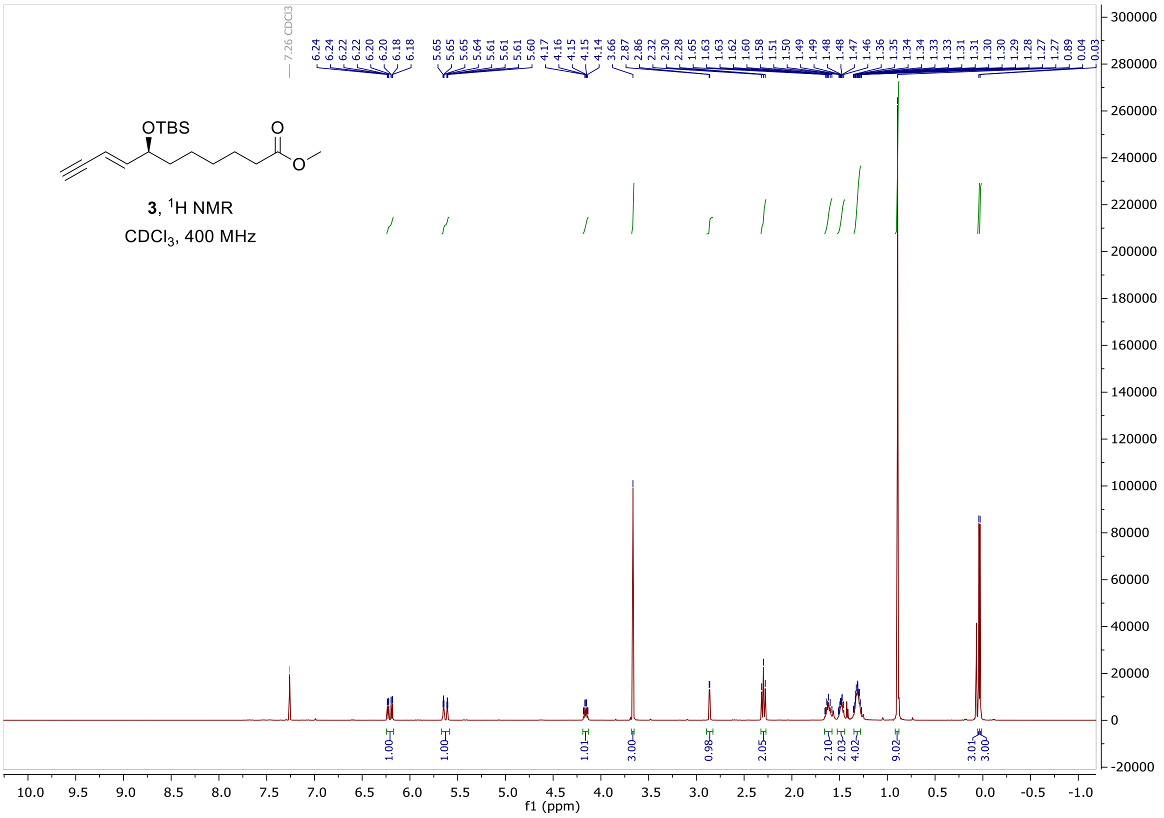

Figure S-35. ^{1}H NMR spectrum of compound **3**.

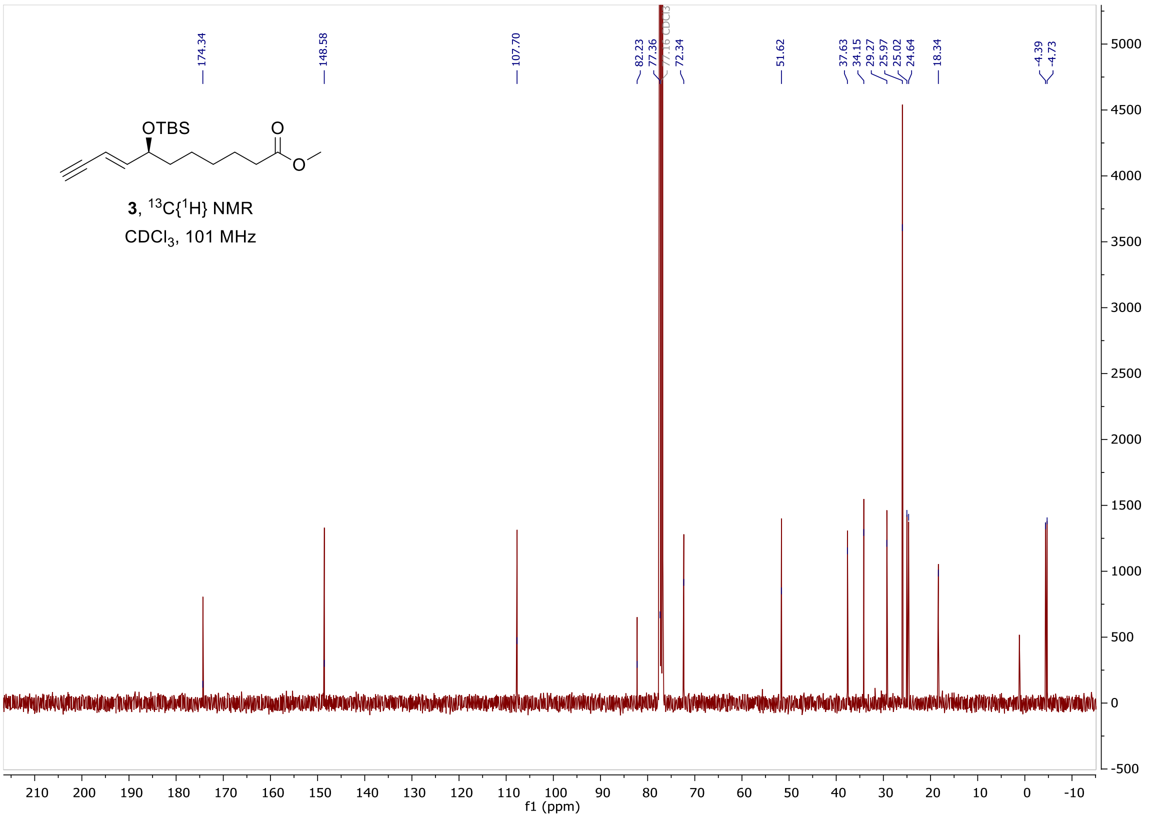

Figure S-36. ^{13}C{^{1}H} NMR spectrum of compound **3**.

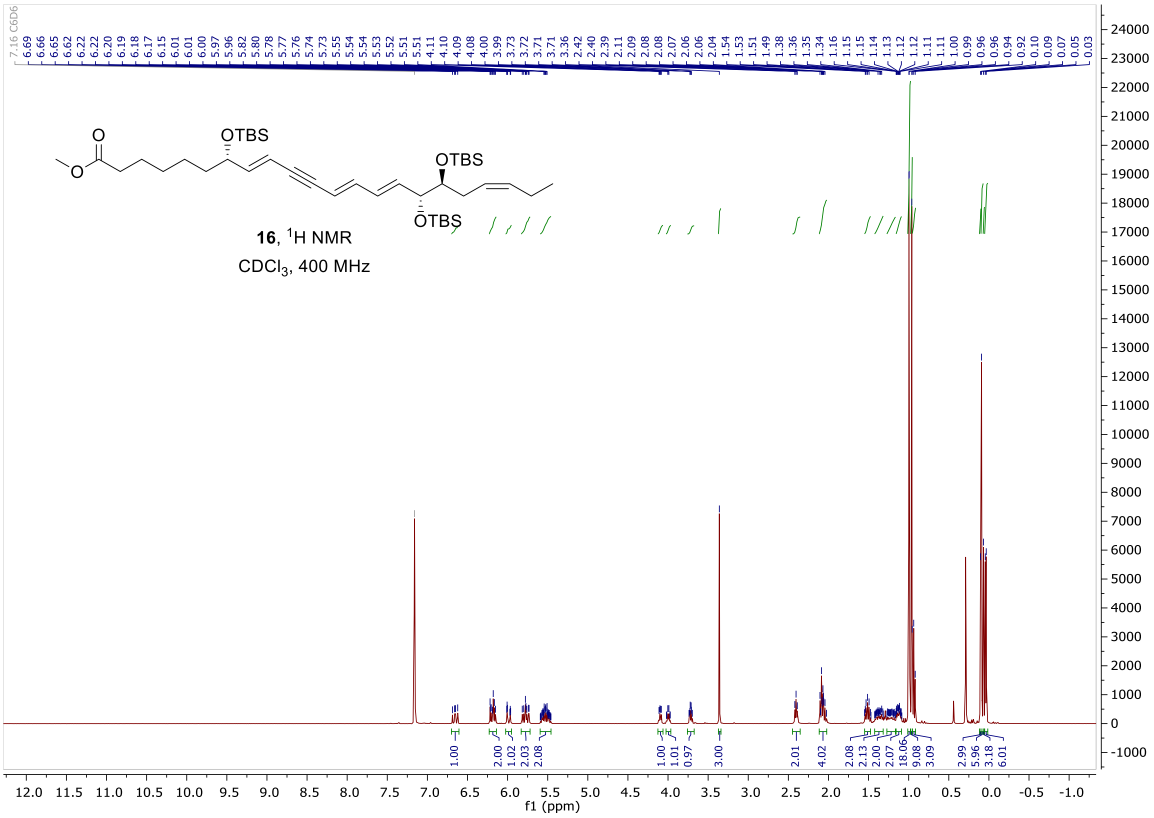

Figure S-37. ^{1}H NMR spectrum of compound **16**.

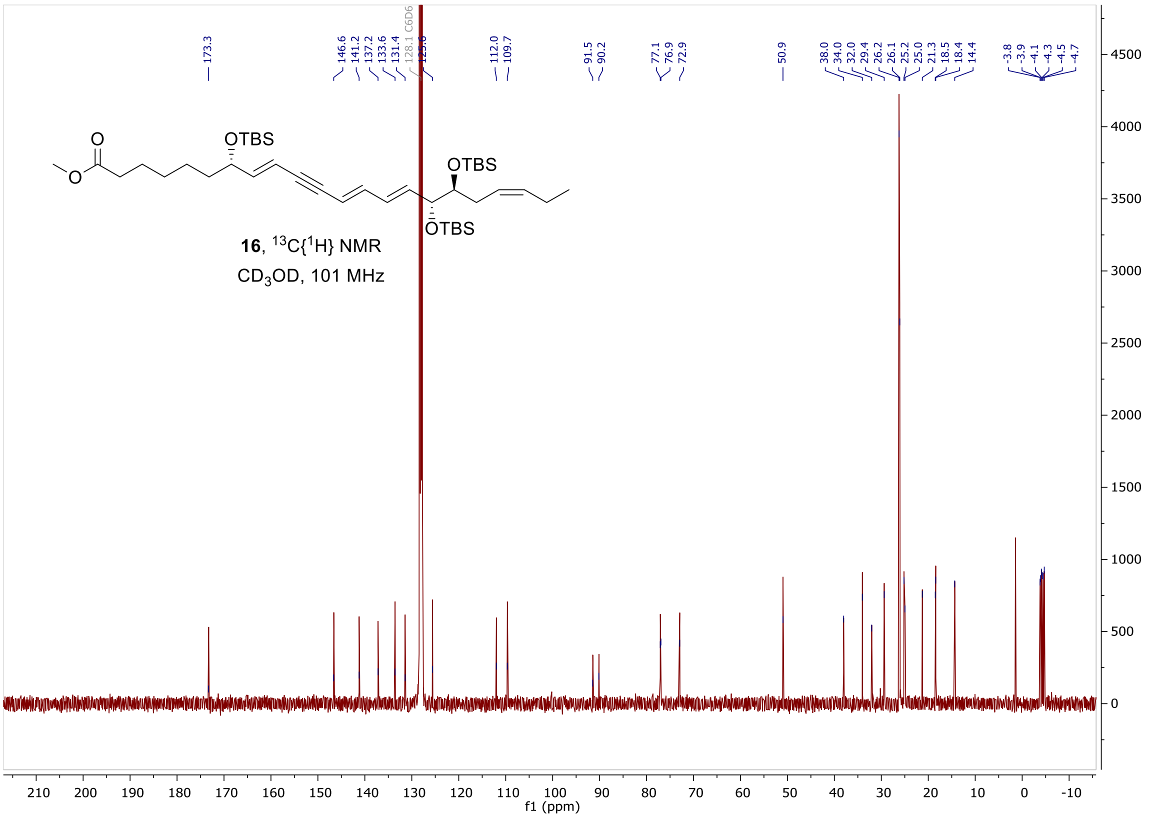

Figure S-38. $^{13}C\{^{1}H\}$ NMR spectrum of compound **16**.

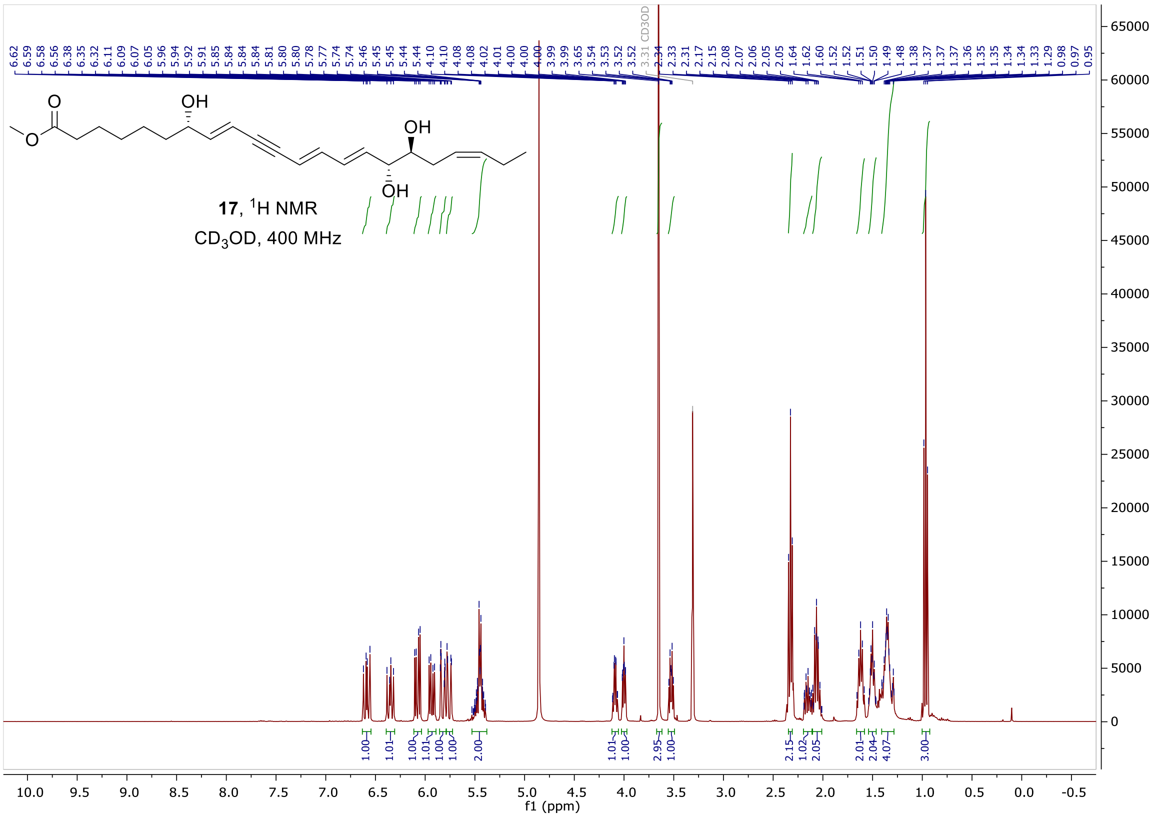

Figure S-39. ^{1}H NMR spectrum of compound **17**.

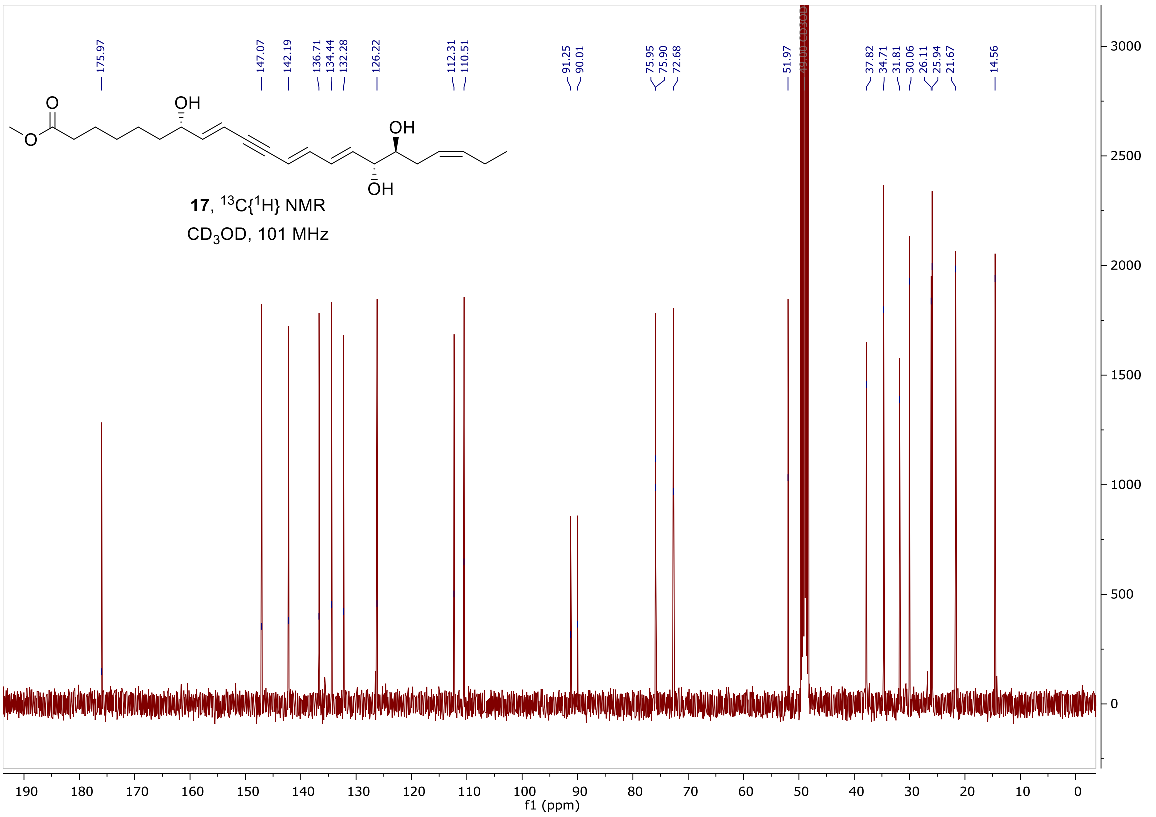

Figure S-40. ^{13}C{^{1}H} NMR spectrum of compound **17**.

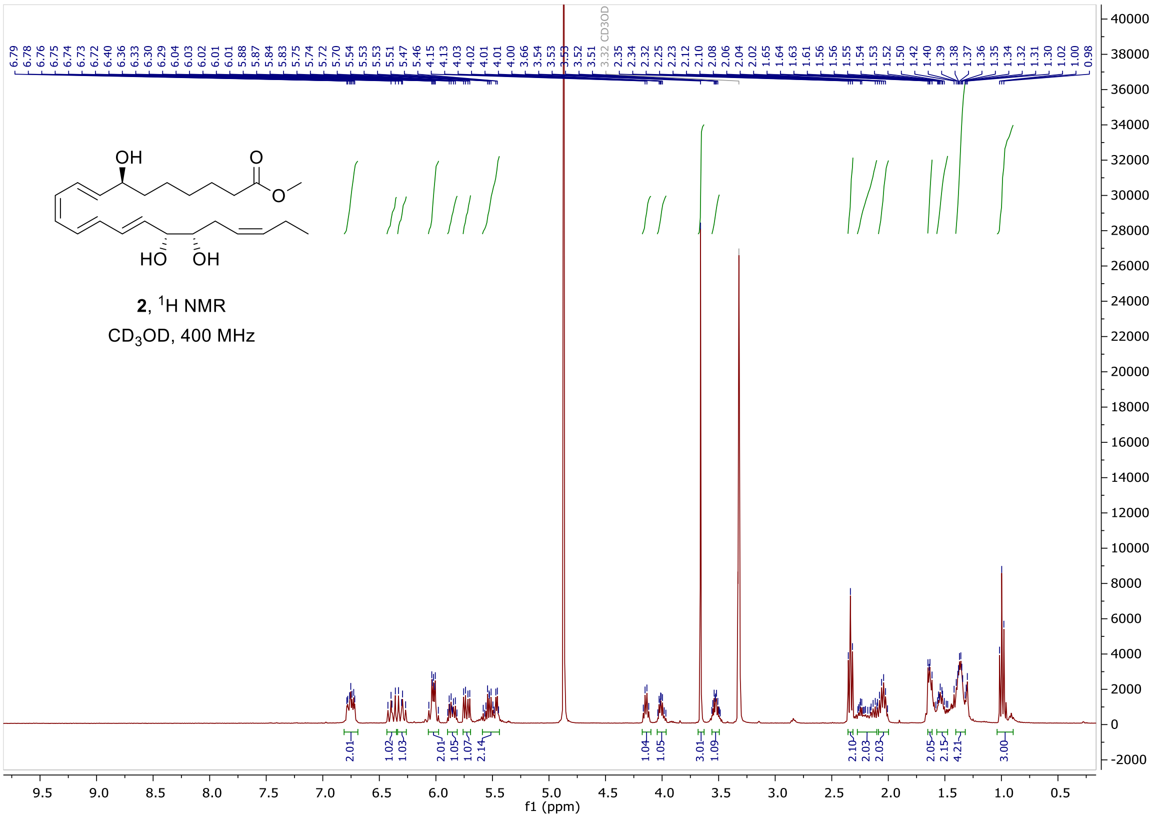

Figure S-41. ^{1}H NMR spectrum of RvD2$_{\text{n-3 DPA}}$ methyl ester **2**.

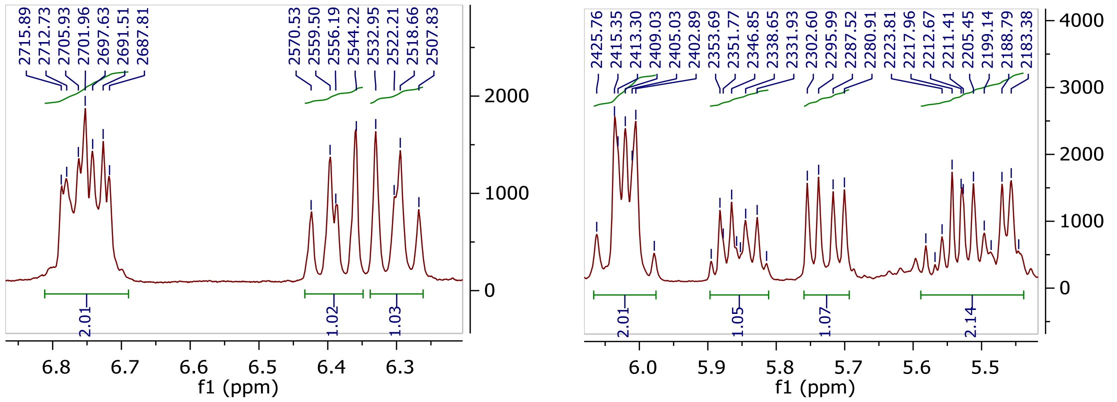

Figure S-42. Expansion of the olefinic region in the ^{1}H NMR spectrum of RvD2$_{\text{n-3 DPA}}$ methyl ester **2**.

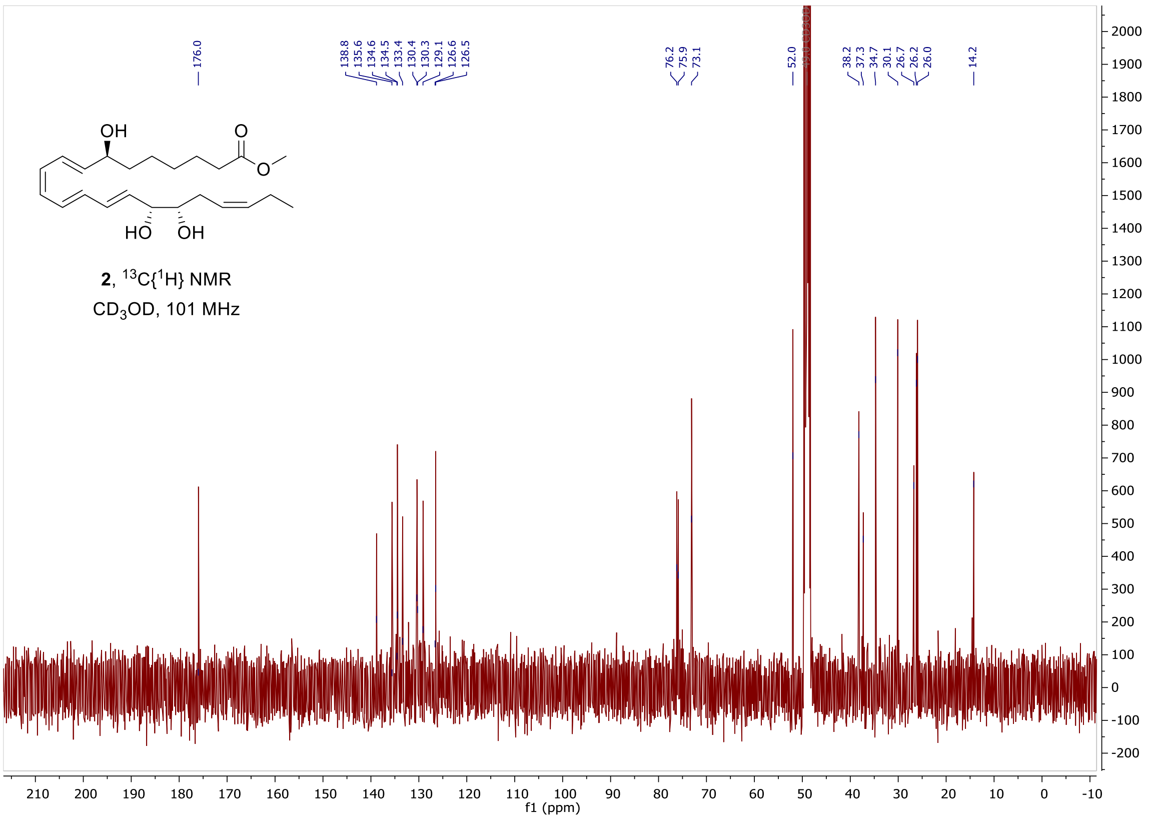

Figure S-43. $^{13}C\{^{1}H\}$ NMR spectrum of RvD2$_{n\text{-}3\ DPA}$ methyl ester **2**.

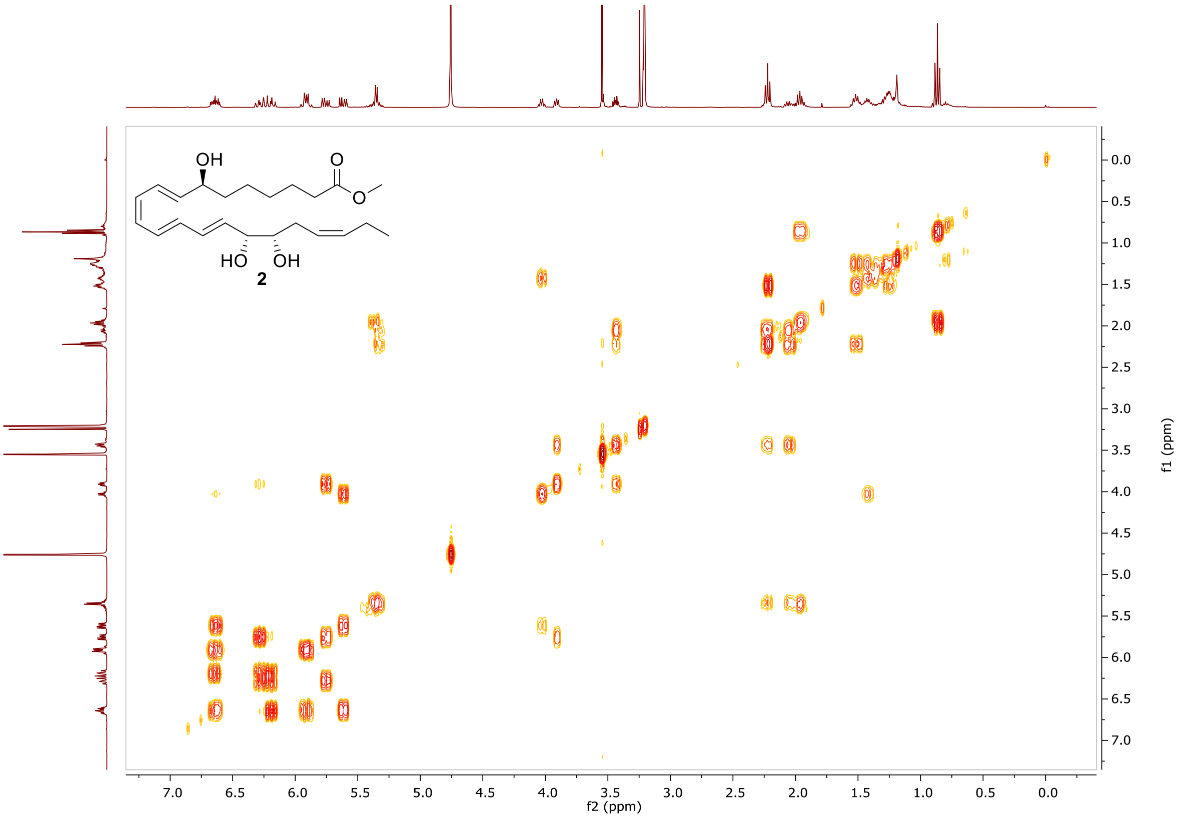

Figure S-44. COSYGP spectrum of RvD2$_{n\text{-}3\ DPA}$ methyl ester **2**.

HPLC Chromatograms

```
Data File C:\Chem32\2\Data\Naft midland Rac AFR2 2020-09-02 07-54-48.D
Sample Name: Naft midland Rac AFR2

    =====================================================================
    Acq. Operator    : SYSTEM
    Sample Operator : SYSTEM
    Acq. Instrument : LC1260-1                        Location :   P1-A-01
    Injection Date  : 9/2/2020 7:55:33 AM
                                                   Inj Volume : 15.000 µl
    Acq. Method      : C:\CHEM32\2\METHODS\Krial 10% iPrOH i heksan.M
    Last changed     : 9/2/2020 8:14:09 AM by SYSTEM
                       (modified after loading)
    Analysis Method : C:\CHEM32\2\METHODS\Krial 10% iPrOH i heksan.M
    Last changed     : 9/2/2020 8:17:45 AM by SYSTEM
                       (modified after loading)
    Sample Info      : AD-H kolonne, 254 nm, 1 mL/min, 1% iPrOH i heksan
                       Rac naft Amalie

    Additional Info : Peak(s) manually integrated
```

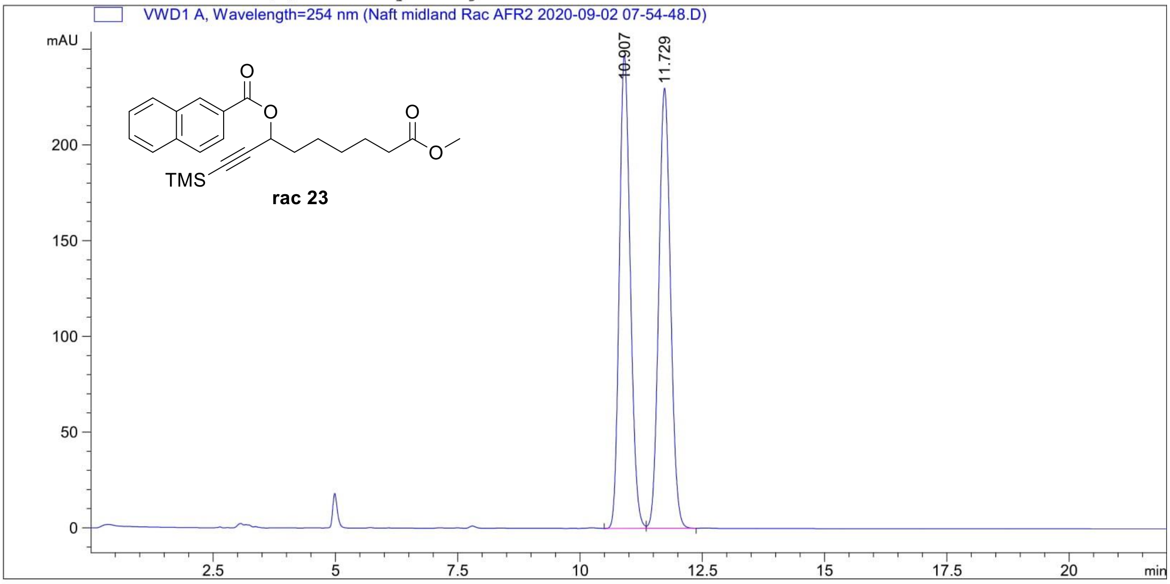


```
    =====================================================================
                             Area Percent Report
    =====================================================================

    Sorted By             :      Signal
    Multiplier            :      1.0000
    Dilution              :      1.0000
    Use Multiplier & Dilution Factor with ISTDs


    Signal 1: VWD1 A, Wavelength=254 nm

    Peak RetTime Type  Width      Area       Height      Area
      #   [min]        [min]    [mAU*s]      [mAU]         %
    ----|-------|----|-------|----------|----------|--------|
       1  10.907 BV    0.2413 3839.74780  247.06580  49.9741
       2  11.729 VB    0.2601 3843.72021  229.89789  50.0259

    Totals :                  7683.46802  476.96368
```

Figure S-45. HPLC chromatogram of racemic **23**.

Data File C:\Chem32\2\Data\Naft midland optisk AFR 2020-09-02 08-19-06.D
Sample Name: Naft midland optisk AFR

===

Acq. Operator : SYSTEM
Sample Operator : SYSTEM
Acq. Instrument : LC1260-1 Location : P1-A-01
Injection Date : 9/2/2020 8:19:51 AM
Inj Volume : 15.000 µl
Acq. Method : C:\CHEM32\2\METHODS\Krial 10% iPrOH i heksan.M
Last changed : 9/2/2020 8:17:45 AM by SYSTEM
(modified after loading)
Analysis Method : C:\CHEM32\2\METHODS\Krial 10% iPrOH i heksan.M
Last changed : 9/2/2020 8:43:38 AM by SYSTEM
(modified after loading)
Sample Info : AD-H kolonne, 254 nm, 1 mL/min, 1% iPrOH i heksan
optisk (S) naft Amalie

Additional Info : Peak(s) manually integrated

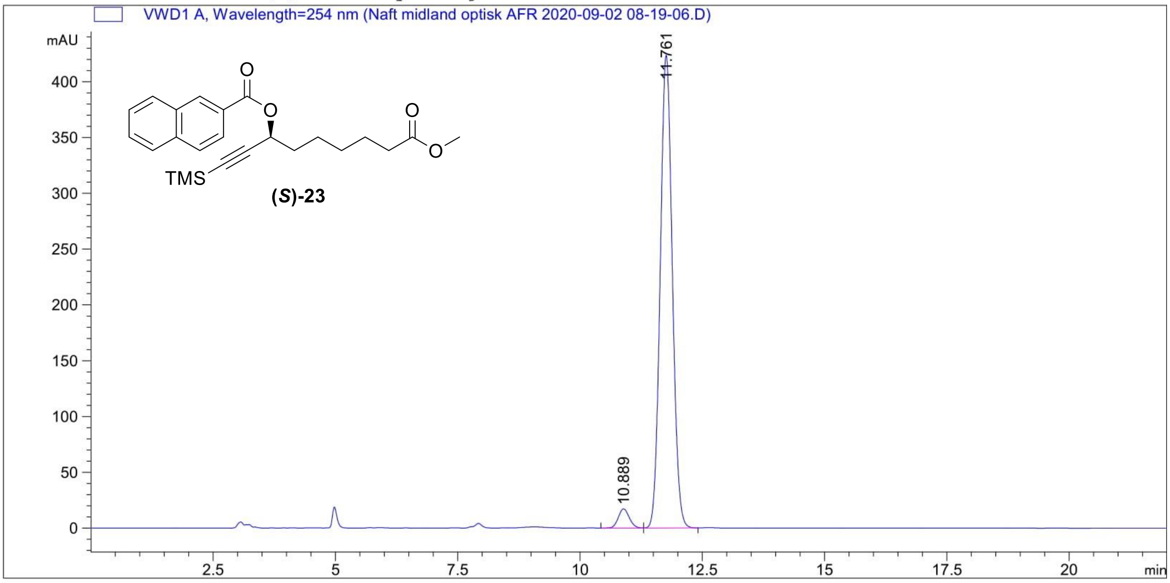

===

Area Percent Report

===

Sorted By : Signal
Multiplier : 1.0000
Dilution : 1.0000
Use Multiplier & Dilution Factor with ISTDs

Signal 1: VWD1 A, Wavelength=254 nm

Peak #	RetTime [min]	Type	Width [min]	Area [mAU*s]	Height [mAU]	Area %
1	10.889	BV	0.2432	270.33051	17.21600	3.5902
2	11.761	VB	0.2670	7259.40918	423.51501	96.4098

Totals : 7529.73969 440.73101

Figure S-46. HPLC chromatogram of (*S*)-**23**.

Data File C:\Chem32\2\Data\RvD2n-3 DPA Me KCN_Zn 2021-01-29 13-32-24.D
Sample Name: RvD2n-3 DPA Me KCN/Zn

===

Acq. Operator : SYSTEM
Sample Operator : SYSTEM
Acq. Instrument : LC1260-1 Location : P1-A-01
Injection Date : 1/29/2021 1:33:06 PM
Inj Volume : 10.000 µl
Acq. Method : C:\CHEM32\2\METHODS\uio-test.M
Last changed : 1/29/2021 1:31:14 PM by SYSTEM
(modified after loading)
Analysis Method : C:\CHEM32\2\METHODS\uio-test.M
Last changed : 1/29/2021 1:53:50 PM by SYSTEM
(modified after loading)
Sample Info : 1 ml/min, C-18 kolonne, 70% MeOH i H2O, UV-254

Additional Info : Peak(s) manually integrated

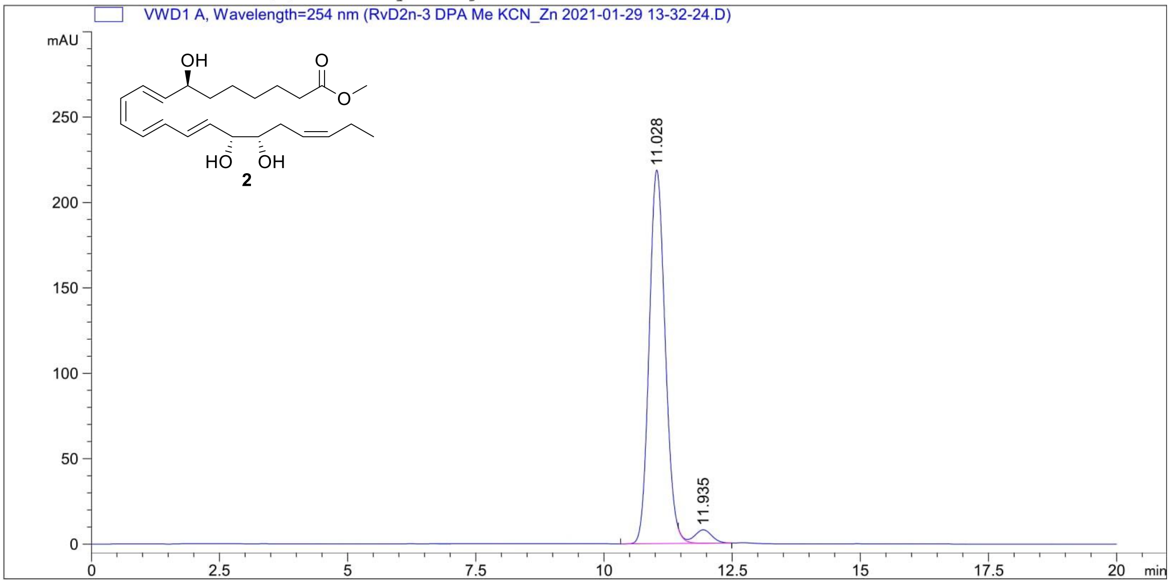

===

Area Percent Report

===

Sorted By : Signal
Multiplier : 1.0000
Dilution : 1.0000
Use Multiplier & Dilution Factor with ISTDs

Signal 1: VWD1 A, Wavelength=254 nm

Peak #	RetTime [min]	Type	Width [min]	Area [mAU*s]	Height [mAU]	Area %
1	11.028	BV R	0.3389	4760.36719	218.78113	96.3047
2	11.935	VB E	0.3588	182.66048	7.84069	3.6953

Totals : 4943.02766 226.62182

Figure S-47. HPLC chromatogram of RvD2$_{n\text{-}3\ DPA}$ methyl ester (**2**).

UV-Vis Chromatograms

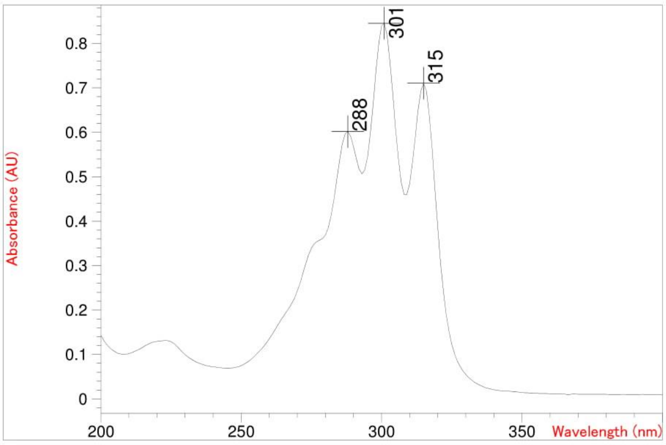

Sample/Result Table

#	Name	Peaks(nm)	Abs(AU)	#	Name	Peaks(nm)	Abs(AU)
1		301.0	0.84501	1		288.0	0.60171
1		315.0	0.71037				

Figure S-48. UV-Vis chromatogram of RvD2$_{\text{n-3 DPA}}$ methyl ester (**2**).

HRMS Chromatograms

Elemental Analysis Report

Analysis Info

Sample Name	AFR-95	Acquisition Date	9/2/2020 9:38:35 AM
Method	ESI_pos_50_1500_os.m	Analysis Name	D:\Data\maxis2020\16939.d

Acquisition Parameter

Source Type	ESI	Ion Polarity	Positive	Set Nebulizer	0.4 Bar
Focus	Not active	Set Capillary	3500 V	Set Dry Heater	200 °C
Scan Begin	50 m/z	Set End Plate Offset	-500 V	Set Dry Gas	4.0 l/min
Scan End	1500 m/z	Set Charging Voltage	2000 V	Set Divert Valve	Waste
		Set Corona	0 nA	Set APCI Heater	0 °C

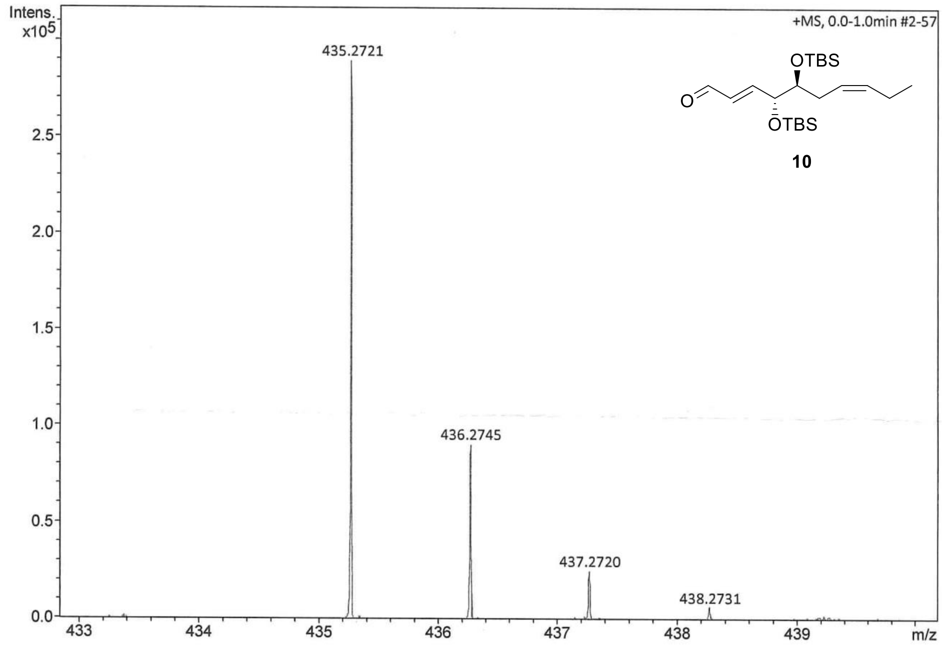

Meas. m/z	Ion Formula	m/z	err [ppm]
435.2721	C28H39O2Si	435.2714	-1.7
	C22H44NaO3Si2	435.2721	0.0
	C25H39O6	435.2741	4.6
	C24H36N4NaO2	435.2730	2.1
	C22H31N10	435.2728	1.5
	C23H40NaO6	435.2717	-0.9

Figure S-49. HRSM spectrum of compound **10**.

Elemental Analysis Report

Analysis Info

Sample Name	AFR-98	Acquisition Date	9/2/2020 9:20:43 AM
Method	ESI_pos_50_1500_os.m	Analysis Name	D:\Data\maxis2020\16938.d

Acquisition Parameter

Source Type	ESI	Ion Polarity	Positive	Set Nebulizer	0.4 Bar
Focus	Not active	Set Capillary	3500 V	Set Dry Heater	200 °C
Scan Begin	50 m/z	Set End Plate Offset	-500 V	Set Dry Gas	4.0 l/min
Scan End	1500 m/z	Set Charging Voltage	2000 V	Set Divert Valve	Waste
		Set Corona	0 nA	Set APCI Heater	0 °C

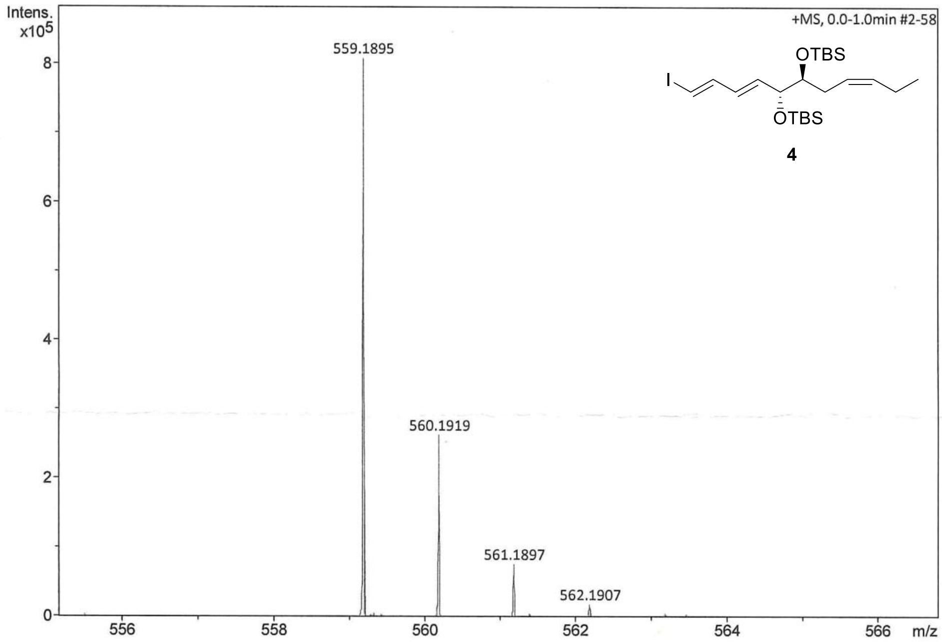

Meas. m/z	Ion Formula	m/z	err [ppm]
559.1895	C29H40IOSi	559.1888	-1.4
	C23H45INaO2Si2	559.1895	-0.0
	C25H32N2NaO11	559.1898	0.5
	C23H20N16NaO	559.1898	0.5
	C23H27N8O9	559.1896	0.0
	C29H28N6NaO3Si	559.1884	-2.0
	C24H36N2NaO8Si2	559.1902	1.3
	C30H31N2O7Si	559.1895	-0.0
	C25H37IN4NaO	559.1904	1.6
	C24H41INaO5	559.1891	-0.8
	C39H27O4	559.1904	1.5
	C38H24N4Na	559.1893	-0.4
	C36H32NaOSi2	559.1884	-2.0

Figure S-50. HRSM spectrum of compound **4**.

Elemental Analysis Report

Analysis Info

Sample Name	AFR-96	Acquisition Date	9/1/2020 11:52:34 AM
Method	ESI_pos_50_1500_os.m	Analysis Name	D:\Data\maxis2020\16934.d

Acquisition Parameter

Source Type	ESI	Ion Polarity	Positive	Set Nebulizer	0.4 Bar
Focus	Not active	Set Capillary	3500 V	Set Dry Heater	200 °C
Scan Begin	50 m/z	Set End Plate Offset	-500 V	Set Dry Gas	4.0 l/min
Scan End	1500 m/z	Set Charging Voltage	2000 V	Set Divert Valve	Waste
		Set Corona	0 nA	Set APCI Heater	0 °C

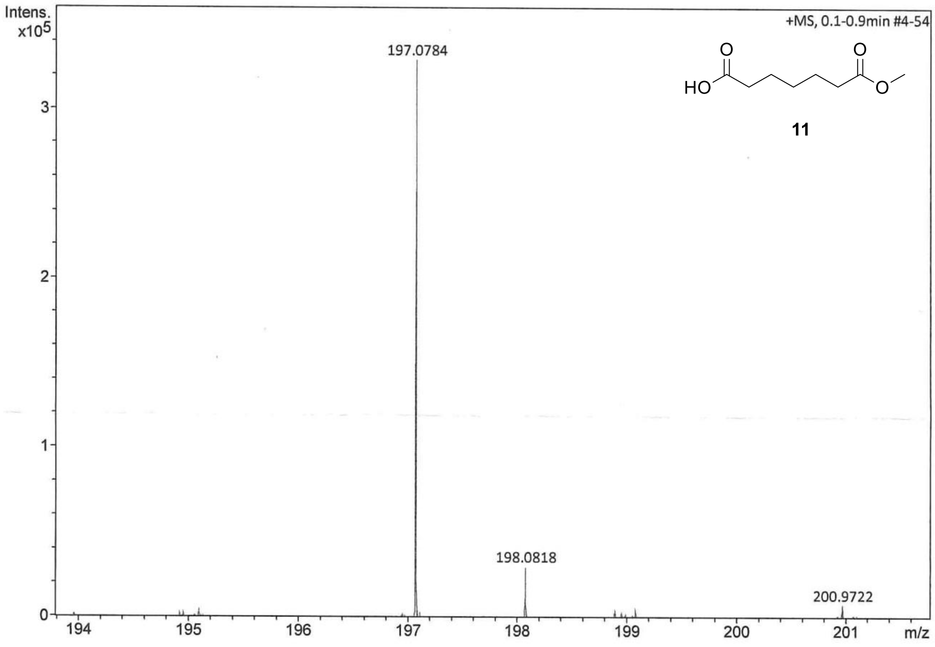

Meas. m/z	Ion Formula	m/z	err [ppm]
197.0784	C8H14NaO4	197.0784	0.0
	C6H9N6O2	197.0781	-1.4
	C8H11N3O3	197.0795	5.4
	C6H12N3NaO3	197.0771	-6.8
	C4H7N9O	197.0768	-8.2
	C9H10N4Na	197.0798	6.8
	C5H13N2O6	197.0768	-8.2

Figure S-51. HRMS spectrum of compound **11**.

Elemental Analysis Report

Analysis Info

Sample Name	AFR-105	Acquisition Date	9/2/2020 8:09:13 AM
Method	ESI_pos_50_1500_os.m	Analysis Name	D:\Data\maxis2020\16935.d

Acquisition Parameter

Source Type	ESI	Ion Polarity	Positive	Set Nebulizer	0.4 Bar
Focus	Not active	Set Capillary	3500 V	Set Dry Heater	200 °C
Scan Begin	50 m/z	Set End Plate Offset	-500 V	Set Dry Gas	4.0 l/min
Scan End	1500 m/z	Set Charging Voltage	2000 V	Set Divert Valve	Waste
		Set Corona	0 nA	Set APCI Heater	0 °C

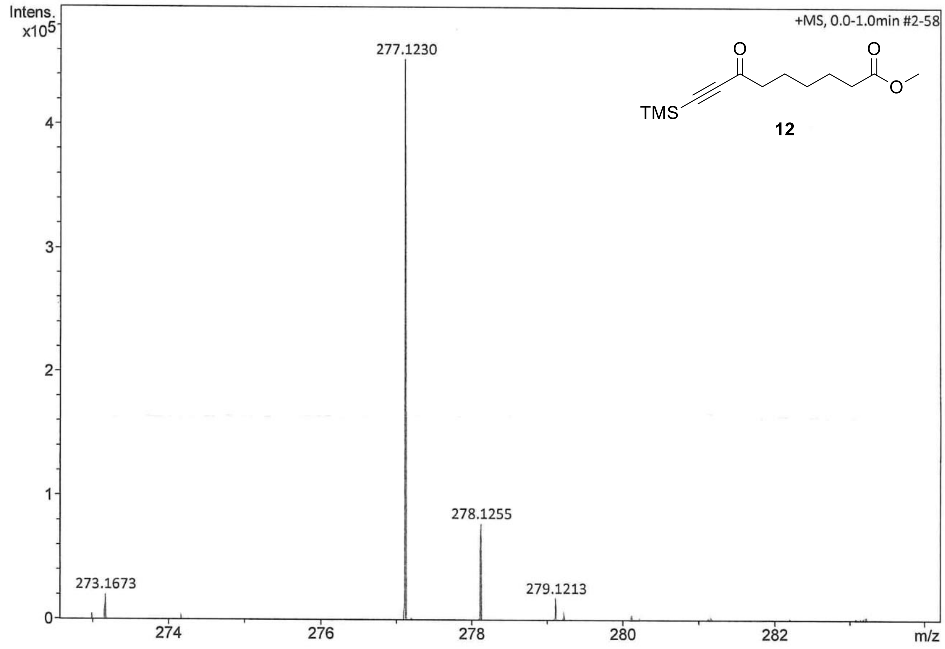

Meas. m/z	Ion Formula	m/z	err [ppm]
277.1230	C10H21N2O5Si	277.1214	-5.7
	C13H22NaO3Si	277.1230	0.1
	C11H17N6OSi	277.1228	-0.9
	C19H17O2	277.1223	-2.6
	C15H21O3Si	277.1254	8.8
	C10H22NaO7	277.1258	10.0

Figure S-52. HRMS spectrum of compound **12**.

Elemental Analysis Report

Analysis Info

Sample Name	AFR-106	Acquisition Date	9/1/2020 11:39:25 AM
Method	ESI_pos_50_1500_os.m	Analysis Name	D:\Data\maxis2020\16933.d

Acquisition Parameter

Source Type	ESI	Ion Polarity	Positive	Set Nebulizer	0.4 Bar
Focus	Not active	Set Capillary	3500 V	Set Dry Heater	200 °C
Scan Begin	50 m/z	Set End Plate Offset	-500 V	Set Dry Gas	4.0 l/min
Scan End	1500 m/z	Set Charging Voltage	2000 V	Set Divert Valve	Waste
		Set Corona	0 nA	Set APCI Heater	0 °C

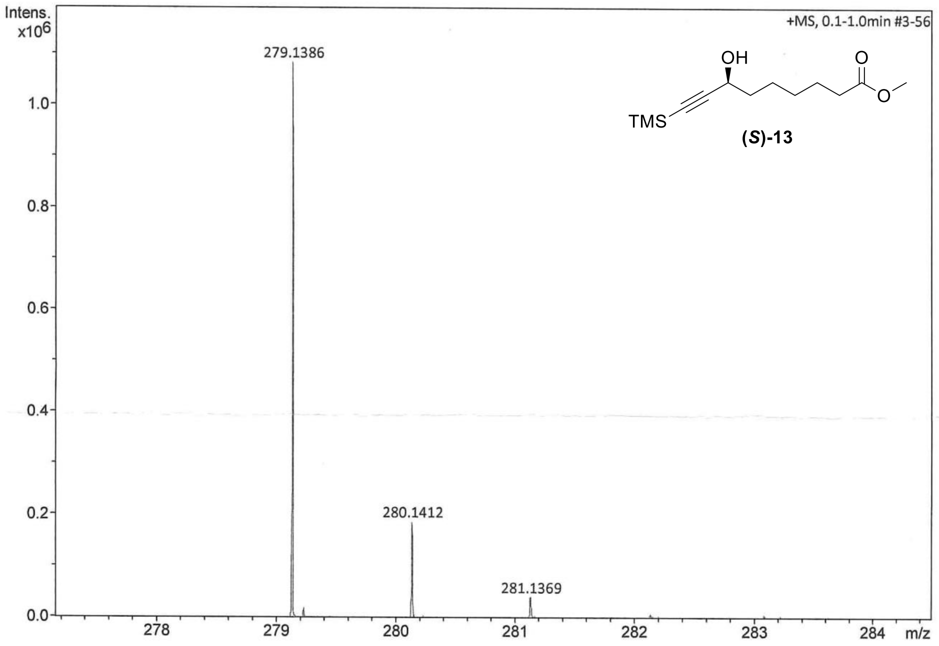

Meas. m/z	Ion Formula	m/z	err [ppm]
279.1386	C11H22N3NaO2Si	279.1373	-4.5
	C13H24NaO3Si	279.1387	0.3
	C11H19N6OSi	279.1384	-0.7
	C13H21N3O2Si	279.1398	4.1
	C19H19O2	279.1380	-2.4

Figure S-53. HRMS spectrum of compound (*S*)-**13**.

Elemental Analysis Report

Analysis Info

Sample Name	AFR-107	Acquisition Date	9/2/2020 10:06:09 AM
Method	ESI_pos_50_1500_os.m	Analysis Name	D:\Data\maxis2020\16940.d

Acquisition Parameter

Source Type	ESI	Ion Polarity	Positive	Set Nebulizer	0.4 Bar
Focus	Not active	Set Capillary	3500 V	Set Dry Heater	200 °C
Scan Begin	50 m/z	Set End Plate Offset	-500 V	Set Dry Gas	4.0 l/min
Scan End	1500 m/z	Set Charging Voltage	2000 V	Set Divert Valve	Waste
		Set Corona	0 nA	Set APCI Heater	0 °C

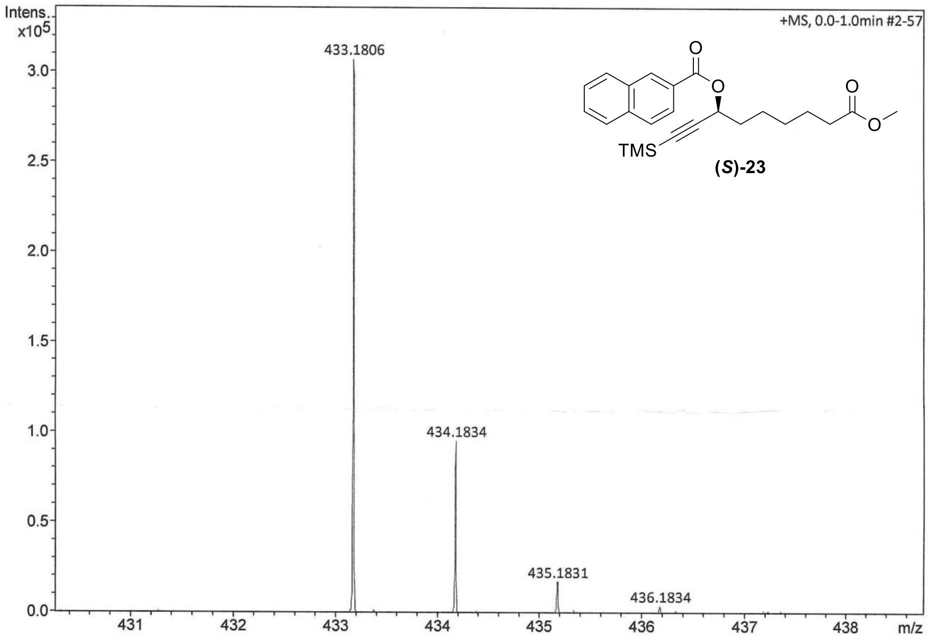

Meas. m/z	Ion Formula	m/z	err [ppm]
433.1806	C30H25O3	433.1798	-1.7
	C22H25N6O2Si	433.1803	-0.7
	C24H30NaO4Si	433.1806	-0.0

Figure S-54. HRMS spectrum of compound (*S*)-**23**.

Elemental Analysis Report

Analysis Info

Sample Name	AFR-110	Acquisition Date	9/8/2020 11:17:50 AM
Method	ESI_pos_50_1500_os.m	Analysis Name	D:\Data\maxis2020\16964.d

Acquisition Parameter

Source Type	ESI	Ion Polarity	Positive	Set Nebulizer	0.4 Bar
Focus	Not active	Set Capillary	3500 V	Set Dry Heater	200 °C
Scan Begin	50 m/z	Set End Plate Offset	-500 V	Set Dry Gas	4.0 l/min
Scan End	1500 m/z	Set Charging Voltage	2000 V	Set Divert Valve	Waste
		Set Corona	0 nA	Set APCI Heater	0 °C

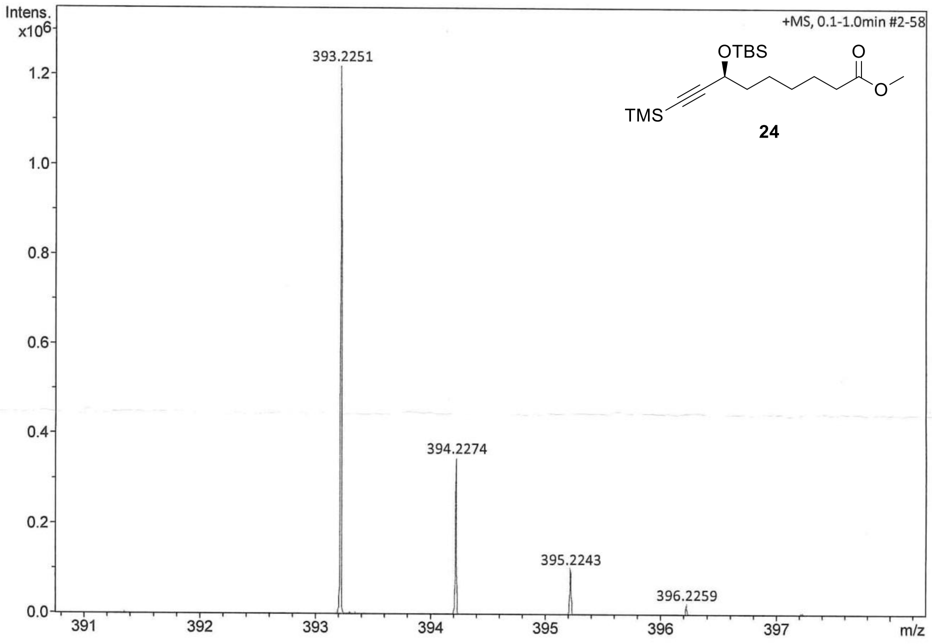

Meas. m/z	Ion Formula	m/z	err [ppm]
393.2251	C25H33O2Si	393.2244	-1.8
	C19H38NaO3Si2	393.2252	0.1
	C19H25N10	393.2258	1.7
	C20H34NaO6	393.2248	-1.0

Figure S-55. HRMS spectrum of compound **24**.

Elemental Analysis Report

Analysis Info

Sample Name	AFR-115	Acquisition Date	9/11/2020 8:26:52 AM
Method	ESI_pos_50_1500_os.m	Analysis Name	D:\Data\maxis2020\16977.d

Acquisition Parameter

Source Type	ESI	Ion Polarity	Positive	Set Nebulizer	0.3 Bar
Focus	Not active	Set Capillary	3500 V	Set Dry Heater	200 °C
Scan Begin	50 m/z	Set End Plate Offset	-500 V	Set Dry Gas	4.0 l/min
Scan End	1500 m/z	Set Charging Voltage	2000 V	Set Divert Valve	Waste
		Set Corona	0 nA	Set APCI Heater	0 °C

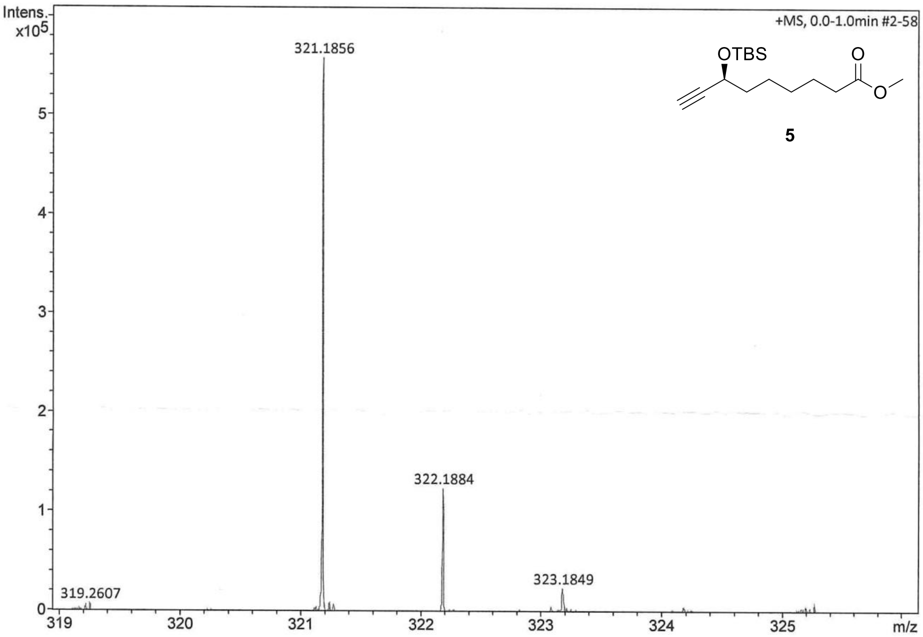

Meas. m/z	Ion Formula	m/z	err [ppm]
321.1856	C14H28N3NaO2Si	321.1843	-3.9
	C14H25N6OSi	321.1854	-0.6
	C16H30NaO3Si	321.1856	0.2
	C22H25O2	321.1849	-2.0
	C16H27N3O2Si	321.1867	3.6

Figure S-56. HRMS spectrum of compound **5**.

Elemental Analysis Report

Analysis Info

Sample Name	AFR-125	Acquisition Date	9/28/2020 11:31:27 AM
Method	ESI_pos_50_1500_os.m	Analysis Name	D:\Data\maxis2020\17064.d

Acquisition Parameter

Source Type	ESI	Ion Polarity	Positive	Set Nebulizer	0.3 Bar
Focus	Not active	Set Capillary	3500 V	Set Dry Heater	200 °C
Scan Begin	50 m/z	Set End Plate Offset	-500 V	Set Dry Gas	4.0 l/min
Scan End	1500 m/z	Set Charging Voltage	2000 V	Set Divert Valve	Waste
		Set Corona	0 nA	Set APCI Heater	0 °C

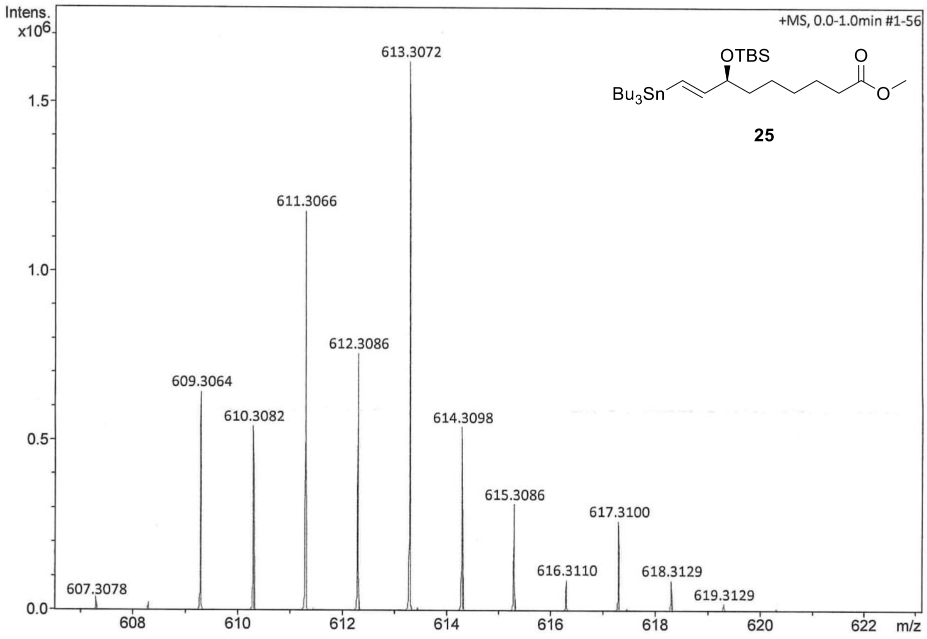

Meas. m/z	Ion Formula	m/z	err [ppm]
609.3064	C29H50NaO10Si	609.3065	0.2
	C28H41N10O4Si	609.3076	1.9
	C27H45N6O8Si	609.3063	-0.3
	C27H38N14NaSi	609.3065	0.2
	C26H42N10NaO4Si	609.3052	-2.0
	C28H58NaO3Si^116Sn	609.3065	0.1
	C26H53N6OSi^116Sn	609.3062	-0.4
	C36H41N4O5	609.3071	1.2
	C35H45O9	609.3058	-1.0
	C35H38N8NaO	609.3061	-0.6
	C34H53O2^116Sn	609.3057	-1.1
	C12H34N26NaOSi	609.3070	1.0
	C23H49N2O16	609.3077	2.0
	C20H34N20NaO2	609.3066	0.3
	C22H46N6NaO12	609.3066	0.3
	C19H38N16NaO6	609.3052	-1.9
	C21H37N16O6	609.3076	2.0
	C18H29N26	609.3063	-0.2
	C20H41N12O10	609.3063	-0.2

Figure S-57. HRMS spectrum of compound **25**.

Elemental Analysis Report

Analysis Info

Sample Name	AFR-119	Acquisition Date	9/16/2020 8:36:12 AM
Method	ESI_pos_50_1500_os.m	Analysis Name	D:\Data\maxis2020\17013.d

Acquisition Parameter

Source Type	ESI	Ion Polarity	Positive	Set Nebulizer	0.3 Bar
Focus	Not active	Set Capillary	3500 V	Set Dry Heater	200 °C
Scan Begin	50 m/z	Set End Plate Offset	-500 V	Set Dry Gas	4.0 l/min
Scan End	1500 m/z	Set Charging Voltage	2000 V	Set Divert Valve	Waste
		Set Corona	0 nA	Set APCI Heater	0 °C

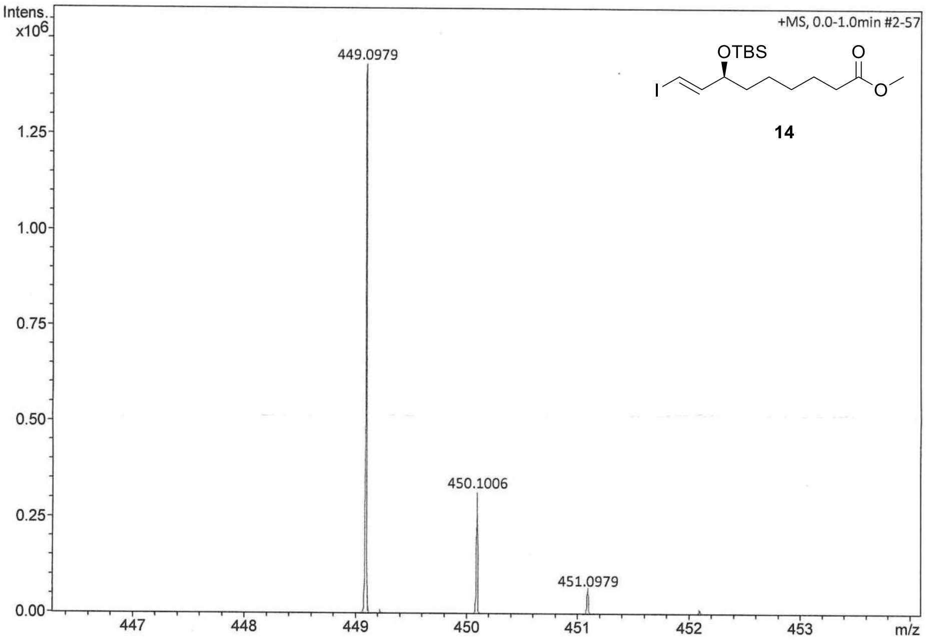

Meas. m/z	Ion Formula	m/z	err [ppm]
449.0979	C14H26IN6OSi	449.0977	-0.5
	C16H31INaO3Si	449.0979	0.1
	C22H26IO2	449.0972	-1.5
	C14H21N4O11Si	449.0971	-1.9
	C15H20N5NaO8Si	449.0973	-1.2
	C14H14N12NaO3Si	449.0973	-1.3
	C23H17N2O8	449.0979	0.1
	C15H17N8O7Si	449.0984	1.1
	C16H23NO12Si	449.0984	1.1
	C14H11N15O2Si	449.0984	1.1
	C17H22N2NaO9Si	449.0987	1.7
	C16H16N9NaO4Si	449.0987	1.7
	C22H11N9O3	449.0979	0.1
	C24H16N3NaO5	449.0982	0.7
	C23H10N10Na	449.0982	0.7
	C29H15N3OSi	449.0979	-0.0

Figure S-58. HRMS spectrum of compound **14**.

Elemental Analysis Report

Analysis Info

Sample Name	AFR-128	Acquisition Date	10/5/2020 1:59:21 PM
Method	ESI_pos_50_1500_os.m	Analysis Name	D:\Data\maxis2020\17099.d

Acquisition Parameter

Source Type	ESI	Ion Polarity	Positive	Set Nebulizer	0.4 Bar
Focus	Not active	Set Capillary	3500 V	Set Dry Heater	200 °C
Scan Begin	50 m/z	Set End Plate Offset	-500 V	Set Dry Gas	4.0 l/min
Scan End	1500 m/z	Set Charging Voltage	2000 V	Set Divert Valve	Waste
		Set Corona	0 nA	Set APCI Heater	0 °C

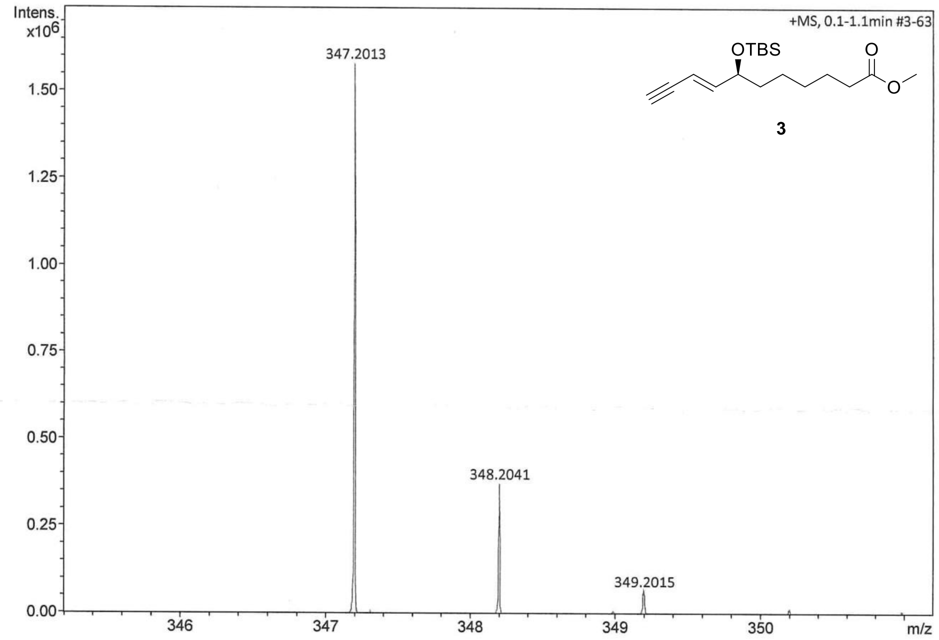

Meas. m/z	Ion Formula	m/z	err [ppm]
347.2013	C16H30N3NaO2Si	347.1999	-3.9
	C16H27N6OSi	347.2010	-0.8
	C15H31N2O5Si	347.1997	-4.7
	C18H32NaO3Si	347.2013	-0.0
	C24H27O2	347.2006	-2.1
	C18H29N3O2Si	347.2024	3.0

Figure S-59. HRMS spectrum of compound **3**.

Elemental Analysis Report

Analysis Info

Sample Name	AFR-136	Acquisition Date	10/19/2020 2:05:15 PM
Method	ESI_pos_50_1500_os.m	Analysis Name	D:\Data\maxis2020\17138.d

Acquisition Parameter

Source Type	ESI	Ion Polarity	Positive	Set Nebulizer	0.3 Bar
Focus	Not active	Set Capillary	3500 V	Set Dry Heater	200 °C
Scan Begin	50 m/z	Set End Plate Offset	-500 V	Set Dry Gas	4.0 l/min
Scan End	1500 m/z	Set Charging Voltage	2000 V	Set Divert Valve	Waste
		Set Corona	0 nA	Set APCI Heater	0 °C

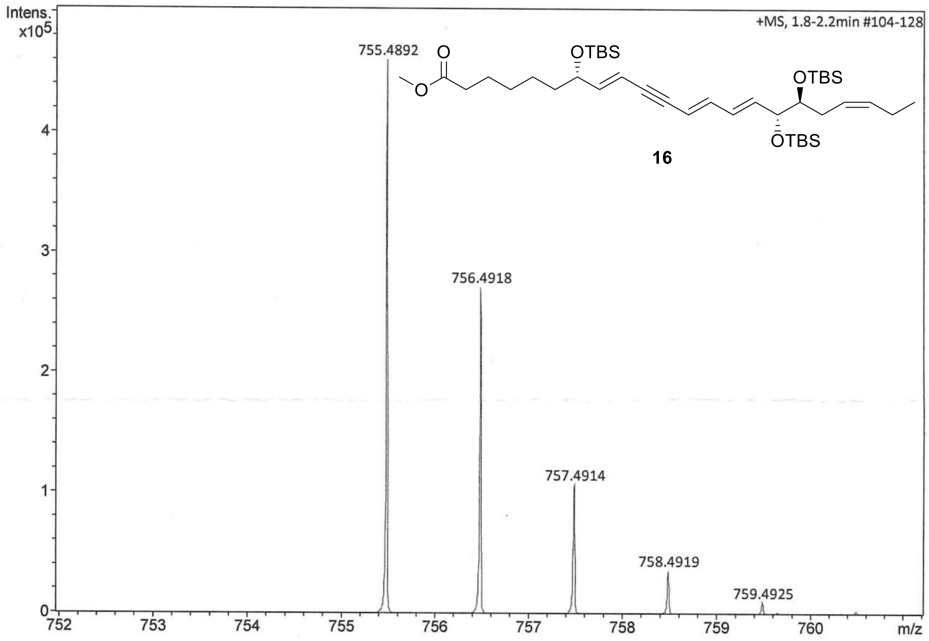

Meas. m/z	Ion Formula	m/z	err [ppm]
755.4892	C46H68N4NaSi2	755.4875	-2.3
	C47H71O4Si2	755.4885	-0.9
	C41H76NaO5Si3	755.4893	0.1
	C48H67N4Si2	755.4899	0.9
	C44H71O8Si	755.4913	2.7
	C42H72N4NaOSi3	755.4906	1.9
	C43H68N4NaO4Si	755.4902	1.3
	C41H63N10O2Si	755.4899	0.9
	C52H64N2NaO	755.4911	2.5
	C42H72NaO8Si	755.4889	-0.5
	C40H67N6O6Si	755.4886	-0.8
	C49H63N4O3	755.4895	0.3
	C48H67O7	755.4881	-1.4

Figure S-60. HRMS spectrum of compound **16**.

Elemental Analysis Report

Analysis Info

Sample Name	AFR-151	Acquisition Date	1/19/2021 1:11:00 PM
Method	ESI_pos_50_1500_os.m	Analysis Name	D:\Data\maxis2021\17426.d

Acquisition Parameter

Source Type	ESI	Ion Polarity	Positive	Set Nebulizer	0.3 Bar
Focus	Not active	Set Capillary	3500 V	Set Dry Heater	200 °C
Scan Begin	50 m/z	Set End Plate Offset	-500 V	Set Dry Gas	4.0 l/min
Scan End	1500 m/z	Set Charging Voltage	2000 V	Set Divert Valve	Waste
		Set Corona	0 nA	Set APCI Heater	0 °C

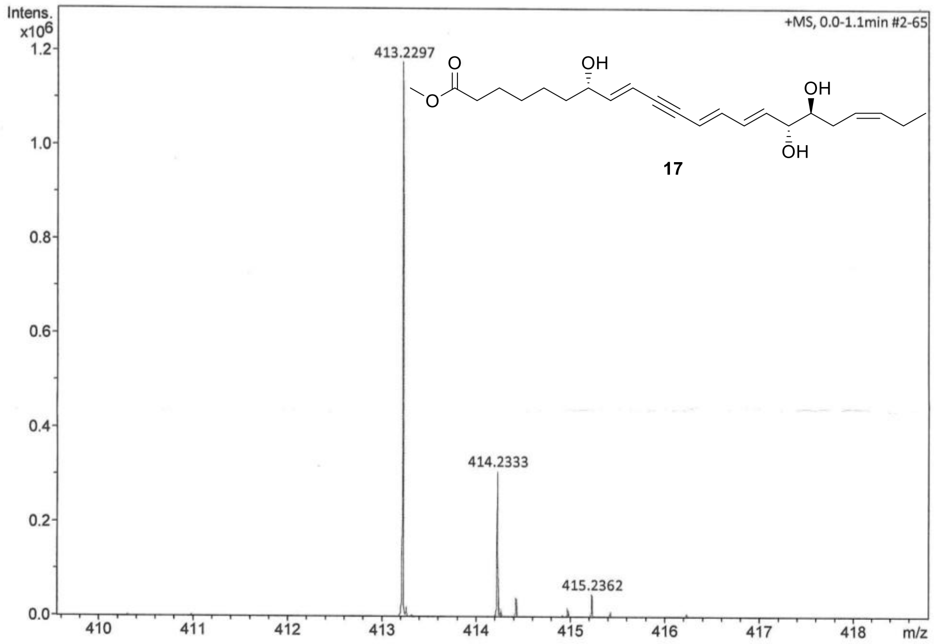

Meas. m/z	Ion Formula	m/z	err [ppm]
413.2297	C23H34NaO5	413.2298	0.2
	C21H29N6O3	413.2296	-0.4
	C24H30N4NaO	413.2312	3.5
	C20H33N2O7	413.2282	-3.7

Figure S-61. HRMS spectrum of compound **17**.

Elemental Analysis Report

Analysis Info

Sample Name	RvD2n-3 DPA metylester	Acquisition Date	11/12/2020 9:01:17 AM
Method	ESI_pos_50_1500_os.m	Analysis Name	D:\Data\maxis2020\17239.d

Acquisition Parameter

Source Type	ESI	Ion Polarity	Positive	Set Nebulizer	0.3 Bar
Focus	Not active	Set Capillary	3500 V	Set Dry Heater	200 °C
Scan Begin	50 m/z	Set End Plate Offset	-500 V	Set Dry Gas	4.0 l/min
Scan End	1500 m/z	Set Charging Voltage	2000 V	Set Divert Valve	Waste
		Set Corona	0 nA	Set APCI Heater	0 °C

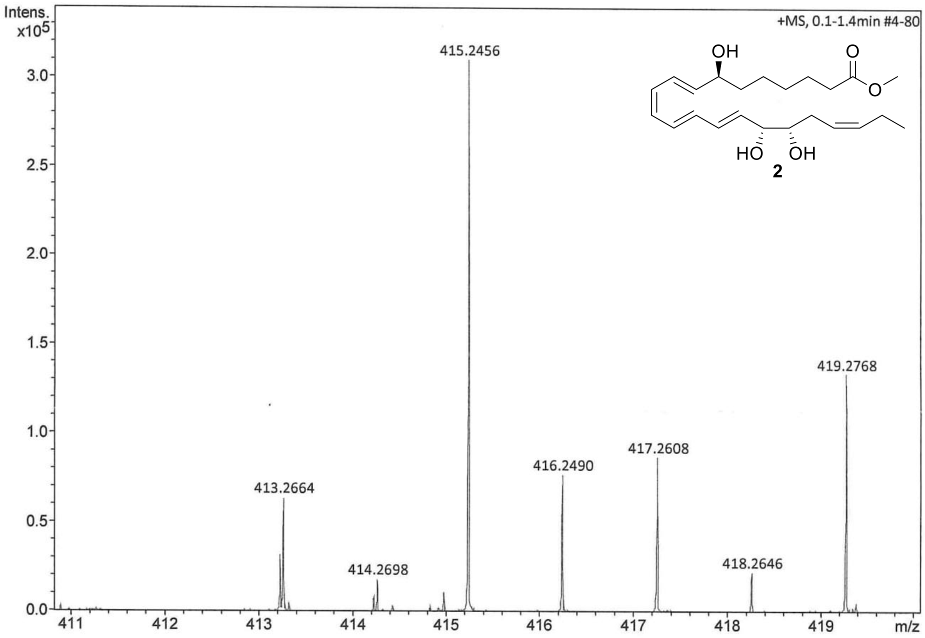

Meas. m/z	Ion Formula	m/z	err [ppm]
415.2456	C23H36NaO5	415.2455	-0.2
	C20H35N2O7	415.2439	-4.1
	C24H32N4NaO	415.2468	3.0
	C21H31N6O3	415.2452	-0.9

Figure S-62. HRMS spectrum of RvD2$_{n\text{-}3\ DPA}$ methyl ester **2**.

References

1. Tungen, J.; Gerstmann, L.; Vik, A.; De Matteis, R.; Colas, R.; Dalli, J.; Chiang, N.; Serhan, C.; Kalesse, M.; Hansen, T. Resolving Inflammation: Synthesis, Configurational Assignment, and Biological Evaluations of $RvD1_{n-3\ DPA}$. *Chemistry,* **2019**, *25*, 1476.
2. Tungen, J. E.; Primdahl, K. G.; Hansen, T. V. The First Total Synthesis of the Lipid Mediator $PD2_{n-3\ DPA}$. *J. Nat. Prod.,* **2020**, *83*, 2255.
3. Takai, K.; Nitta, K.; Utimoto, K. Simple and selective method for RCHO → (E)-RCH=CHX conversion by means of a CHX_3− $CrCl_2$ system. *J. Am. Chem. Soc.,* **1986**, *108*, 7408.
4. Niwayama, S. Highly Efficient Selective Monohydrolysis of Symmetric Diesters. *J. Org. Chem.,* **2000**, *65*, 5834.
5. Rodriguez, A. R.; Spur, B. W. First total syntheses of the pro-resolving lipid mediators 7(*S*),13(*R*),20(*S*)-Resolvin T1 and 7(*S*),13(*R*)-Resolvin T4. *Tetrahedron Lett.,* **2020**, *61*, 151473.
6. Boer, R. E.; Gimnez-Bastida, J. A.; Boutaud, O.; Jana, S.; Schneider, C.; Sulikowski, G. A. Total Synthesis and Biological Activity of the Arachidonic Acid Metabolite Hemiketal E 2. *Org. Lett.,* **2018**, *20*, 4020.
7. Näf, F.; Decorzant, R.; Thommen, W.; Willhalm, B.; Ohloff, G. The Four Isomeric 1,3,5-Undecatrienes. Synthesis and configurational assignment. *Helv. Chim. Acta.,* **1975**, *58*, 1016.
8. Li, J.; Leong, M. M.; Stewart A.; Rizzacasa, M. A. Total synthesis of the endogenous inflammation resolving lipid resolvin D2 using a common lynchpin. *Beilstein J. Org. Chem.*, **2013**, *9*, 2762.
9. Chang, M.; Rao, M. K.; Reddanna, P.; Li, C. H.; Tu, C.-P. D.; Corey, E. J.; Reddy, C. C. I. Specificity of the glutathione *S*-transferases in the conversion of leukotriene A4 to leukotriene C4. *Arch. Biochem. Biophys.,* **1987**, *259*, 536.
10. Palmas, F.; Clarke, J.; Colas, R. A.; Gomez, E. A.; Keogh, A.; Boylan, M.; McEvoy, N.; McElvaney, O. J.; McElvaney, O.; Alalqam, R.; McElvaney, N. G.; Curley, G. F.; Dalli, J. Dysregulated plasma lipid mediator profiles in critically ill COVID-19 patients. *PLoS ONE,* **2021**, *16(8)*, e0256226.
